# The Effect of Cannabidiol on UV-Induced Changes in Intracellular Signaling of 3D-Cultured Skin Keratinocytes

**DOI:** 10.3390/ijms22031501

**Published:** 2021-02-02

**Authors:** Agnieszka Gęgotek, Sinemyiz Atalay, Adelina Rogowska-Wrzesińska, Elżbieta Skrzydlewska

**Affiliations:** 1 Department of Analytical Chemistry, Medical University of Bialystok, Mickiewicza 2D, 15-222 Bialystok, Poland; sinemyiz.atalay@umb.edu.pl (S.A.); elzbieta.skrzydlewska@umb.edu.pl (E.S.); 2 Department of Biochemistry and Molecular Biology, University of Southern Danmark, Campusvej 55, DK-5230 Odense M, Denmark; adelinar@bmb.sdu.dk

**Keywords:** cannabidiol, UV irradiated skin, three-dimensional in vitro culture, keratinocytes, proteomics, lipid peroxidation products adducts formation

## Abstract

Human epidermal keratinocytes are constantly exposed to UV radiation. As a result, there is a significant need for safe and effective compounds to protect skin cells against this environmental damage. This study aimed to analyze the effect of phytocannabinoid-cannabinoid (CBD)-on the proteome of UVA/B irradiated keratinocytes. The keratinocytes were cultured in a three-dimensional (3D) system, designed to mimic epidermal conditions closely. The obtained results indicate that CBD protected against the harmful effects of UVA/B radiation. CBD decreased the expression of proinflammatory proteins, including TNFα/NFκB and IκBKB complex and decreased the expression of proteins involved in de novo protein biosynthesis, which are increased in UVA/B-irradiated cells. Additionally, CBD enhanced the UV-induced expression of 20S proteasome subunits. CBD also protected protein structures from 4-hydroxynonenal (HNE)-binding induced by UV radiation, which primarily affects antioxidant enzymes. CBD-through its antioxidant/anti-inflammatory activity and regulation of protein biosynthesis and degradation-protects skin cells against UVA/B-induced changes. In the future, its long-term use in epidermal cells should be investigated.

## 1. Introduction

Keratinocytes, which form the external layers of the human epidermis, are constantly exposed to harmful environmental factors that disrupt their metabolism. As a result of keratinocyte dysfunction, the synthesis of intercellular cement, keratinization, and epidermal continuity is disrupted [1]. UV radiation has been described as one of the main physical factors that affect skin cells daily [2]. The UV light which naturally reaches the Earth’s surface contains UVB (280–320 nm) and UVA (320–400 nm) radiation. Each has unique properties, including energy, depth of penetration, and biological effects.

Exposure of skin cells to UV radiation promotes the generation of reactive oxygen species (ROS) and disturbances in the antioxidant system, leading to oxidative stress [3]. As a result, proteins, lipids, and nucleic acids are altered by oxidative modifications. These modified biomolecules interact with other molecules and form complex adducts. For example, reactive products of lipid peroxidation often bind to proteins [4]. These modifications may affect protein function, especially of antioxidant, proinflammatory, and proapoptotic factors. Consequently, membrane permeability, cell signaling, enzymatic reactions, and gene expression are perturbed [5].

In particular, UV radiation affects intracellular signaling pathways involving enzymatic lipid metabolism products, including endocannabinoids [3]. It has been previously shown that UV radiation decreases the level of endocannabinoids (such as anandamide and 2-AG) in skin cells. UV radiation also enhances the expression of membrane endocannabinoid receptors CB1, CB2, and TRPV1, which significantly influences the cytoprotective and proinflammatory properties of these cells [3]. Therefore, the search for compounds that could protect skin cells against UV radiation has been directed at substances that target the endocannabinoid system. An example of such a compound is cannabidiol (CBD), derived from *Cannabis sativa* L.

**Figure 1:**
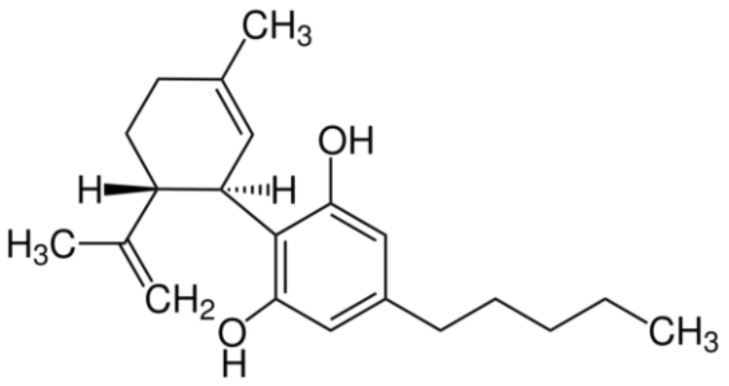
Chemical structure of cannabidiol (CBD).

CBD is one of the few phytocannabinoids without psychoactive effects. However, it has strong anti-inflammatory activity [6]. Moreover, its chemical structure (Figure 1) and interactions with both endocannabinoids and transmembrane receptors suggest that CBD has antioxidant properties [7]. Hence, far, CBD treatment of cells under oxidative stress has been shown to decrease the activity of pro-oxidative enzymes such as xanthine and NADPH oxygenase and prevents superoxide anion generation [8]. Moreover, in UV irradiated keratinocytes, CBD prevents dysfunction in the antioxidant system associated with glutathione peroxidase and thioredoxin reductase activity and activates the cytoprotective Nrf2/ARE pathway [9,10]. The antioxidant effect of CBD prevents changes in the structure and function of the cell membrane and supports cell viability under oxidative conditions [8,11,12]. Moreover, CBD by interacting with membrane cannabinoid receptor CB2 reduces inflammation significantly by decreasing TNFα level [7]. This, combined with CBD induced decrease in lymphocyte proliferation, also reduces the proinflammatory response in organisms [13,14,15]. For these reasons, CBD has been widely considered as a potential therapeutic compound for many disease states, including skin diseases such as psoriasis [16], as well as various types of cancer [17,18]. However, to use CBD safely and effectively, a comprehensive understanding of its effects on cell metabolism is necessary. Therefore, research into its influence on various aspects of cellular metabolism is still ongoing. One such approach is proteomic analysis, which profiles all proteins and their structures.

Previously published data also show that the cell culture model plays a major role in the metabolic changes that occur as a result of CBD treatment [19]. Commonly used in vitro models involving only two-dimensional (2D) cell cultures cannot model the metabolic changes in cells that create multilayer structures, such as the skin. Moreover, 2D models do not allow the assessment of cell responses to the effects of factors penetrating deep into multilayer structures [20]. Several studies have shown that different models (2D or 3D) produce different cellular responses [19,21,22,23]. Using the most common 2D cell culture model allowed to examine the mechanism of changes taking place inside the cell directly to the studied stimuli; however, the reactions of cells in a living organism are more constrained due to the interactions between cells forming more complex structures. Therefore, the application of the 3D culture model provides cells the possibility of interactions between them, which additionally, in the case of skin cells, brings the experimental model closer to the epidermal conditions [24]. This is particularly important when analyzing the metabolism of skin cells exposed to UV radiation at different wavelengths, as well as the effects of exogenous substances such as CBD [19]. Therefore, the aim of this study was to analyze the effect of CBD on proteomic changes in UVA and UVB-irradiated keratinocytes cultured in a three-dimensional (3D) system to see if CBD can stimulate antioxidant and inhibit proinflammatory proteins.

## 2. Results

This study highlights the effect of CBD on the proteome of UV irradiated 3D cultured keratinocytes. CBD has potent antioxidant and anti-inflammatory effects. Therefore, we expected the most significant changes among proteins with such functions. Our results partially support this hypothesis, with large changes in proteins involved in the inflammatory response. However, molecules involved in de novo protein biosynthesis and their proteolysis showed the second-largest changes in our dataset.

**Figure 2:**
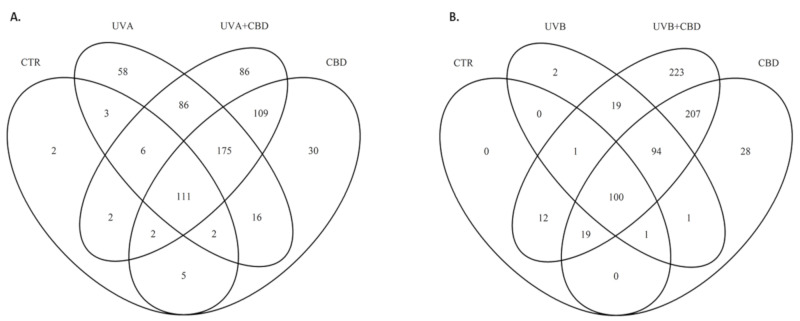
Venn diagram showing protein distribution in the keratinocytes cultured in a three-dimensional culture model and treated with cannabidiol (4 μM) following UVA (30 J/cm^2^) (**A**) or UVB (60 mJ/cm^2^) (**B**) radiation. Data analyzed using RStudio (R version 3.6.2). The names and abundance of proteins are shown in Supplementary Table S1. Abbreviations: Ctr, control; CBD, cannabidiol.

We identified and estimated the levels of 740 proteins across all samples. Because of the high-level of keratin, some proteins with the lowest levels of expression were only found in selected conditions (Supplementary Table S1). Figure 2 shows the distribution of the identified proteins between different treatment options for keratinocytes. In the control cells, only 132 proteins were identified according to established requirements (proteins with at least three identified peptides longer than six amino acid residues and at least two unique peptides). It is unclear whether these proteins were present but not detectable or not expressed at all. Therefore, for further analysis, we imputed the missing values using half of the minimum positive values estimation. In UV irradiated cells, the numbers of identified proteins were 456 and 217 proteins for UVA and UVB treatment, respectively; CBD treatment induced expression of at least 28 proteins not found in other conditions.

**Figure 3:**
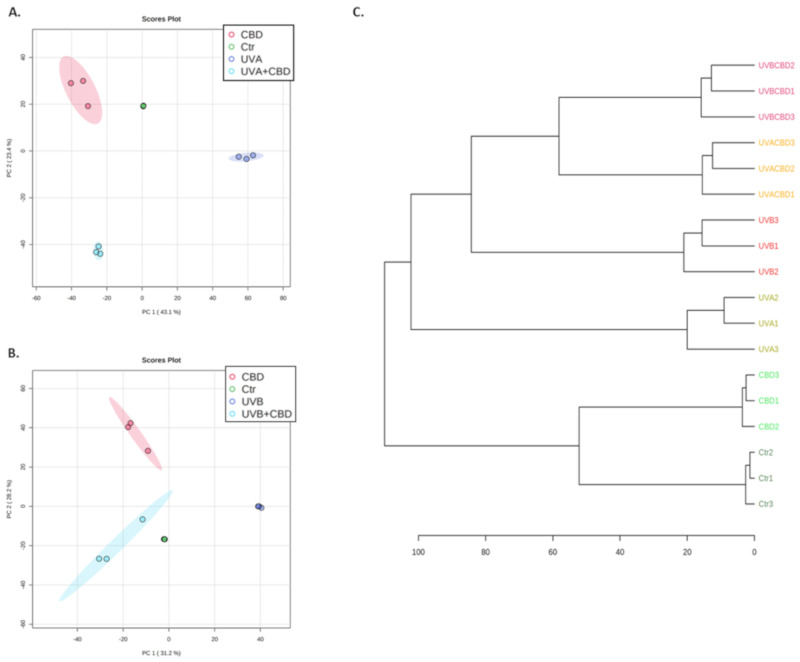
Principal component analysis (PCA) of keratinocytes cultured in a three-dimensional culture model and treated with cannabidiol (4 μM) following UVA (30 J/cm^2^) (**A**) or UVB (60 mJ/cm^2^) (**B**) radiation, as well as a hierarchical dendrogram (**C**) of these samples. Abbreviations: Ctr, control; CBD, cannabidiol; PC, principal component.

Principal component analysis (PCA) clearly shows clustered groups separating each treatment type: UVA (component 1–43.1%; component 2–23.4%; Figure 3A) and UVB treated cells (component 1–31.2%; component 2–28.2%; Figure 3B). UV irradiated samples were grouped on the right part of the diagram, while the rest of the samples cluster on the left side. Hierarchical clustering of samples showed that CBD-treated keratinocytes exhibited the proteomic profile closest to the control cell, despite UV-induced changes. Additionally, changes induced by CBD following UV irradiation led to the clustering of UVA + CBD and UVB + CBD samples on one hierarchical arm, with the UVA and UVB-irradiated samples forming part of a separate cluster (Figure 3C).

**Figure 4:**
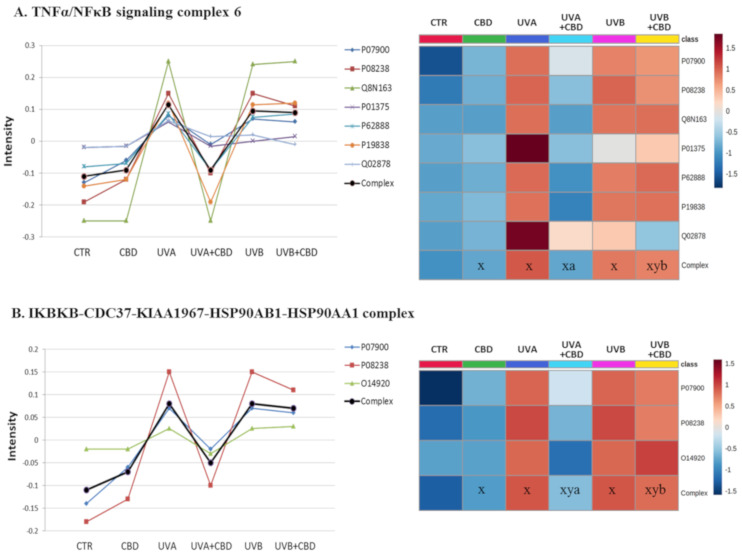
The point graph and heatmap showing the average intensity of proteins creating TNFα/NFκB (**A**) and IκBKB/HSP90 (**B**) complexes in keratinocytes following UVA (30 J/cm^2^) or UVB (60 mJ/cm^2^) radiation and treated with cannabidiol (CBD, 4 μM) in a three-dimensional culture model. Proteins IDs: O14920, inhibitor of nuclear factor κ-B kinase subunit B; P01375, tumor necrosis factor α; P07900, heat shock protein HSP 90A; P08238, heat shock protein HSP 90B; P19838, nuclear factor NFκB p105; P62888, 60S ribosomal protein L30; Q02878, 60S ribosomal protein L6; Q8N163, cell cycle and apoptosis regulator protein 2. Statistical significances showed only for the whole complexes. X: statistically significant differences vs. non-treated cells, *p* < 0.05; y: statistically significant differences vs. CBD-treated cells, *p* < 0.05; a: statistically significant differences vs. UVA-irradiated cells, *p* < 0.05; b: statistically significant differences vs. UVB-irradiated cells, *p* < 0.05.

**Figure 5:**
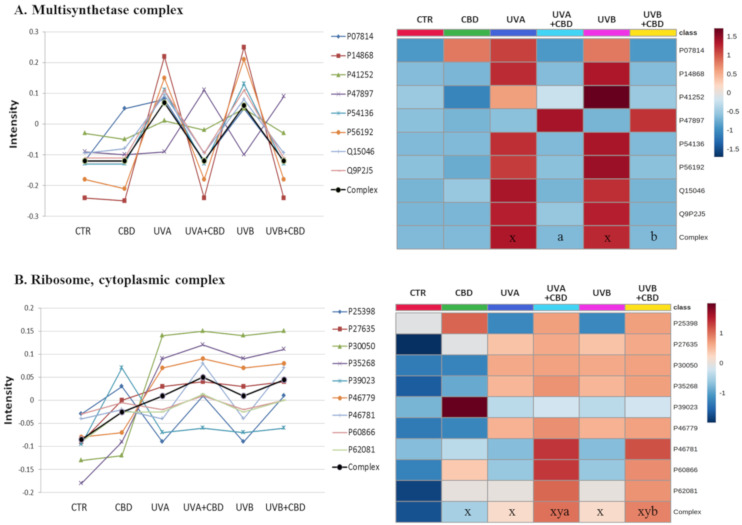
The point graph and heatmap showing the average intensity of proteins creating multisynthetase (**A**) and ribosome (**B**) complexes in keratinocytes following UVA (30 J/cm^2^) or UVB (60 mJ/cm^2^) radiation and treated with cannabidiol (CBD, 4 μM) in a three-dimensional culture model. Proteins IDs: P07814, glutamate/proline-tRNA ligase; P14868, aspartate-tRNA ligase; P41252, isoleucine-tRNA ligase; P47897, glutamine-tRNA ligase; P54136, arginine-tRNA ligase; P56192, methionine-tRNA ligase; Q15046, lysine-tRNA ligase; Q9P2J5, leucine-tRNA ligase; P62081, P46781, P25398, P60866, 40S ribosomal proteins S7, S9, S12, S20; P39023, P27635, P30050, P35268, P46779, 60S ribosomal protein L3, L10, L12, L22, L28. Statistical significances showed only for the whole complexes. x: statistically significant differences vs. non-treated cells, *p* < 0.05; y: statistically significant differences vs. CBD-treated cells, *p* < 0.05; a: statistically significant differences vs. UVA-irradiated cells, *p* < 0.05; b: statistically significant differences vs. UVB-irradiated cells, *p* < 0.05.

**Figure 6:**
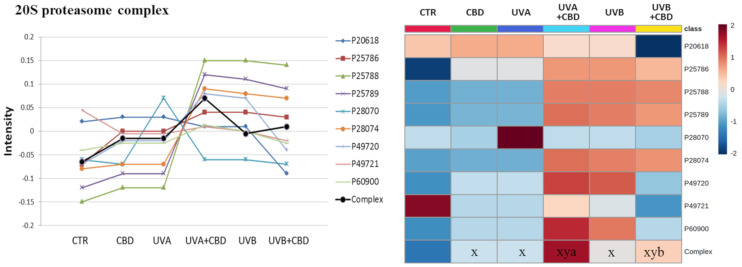
The point graph and heatmap showing the average intensity of proteins creating 20S proteasome complex in keratinocytes following UVA (30 J/cm^2^) or UVB (60 mJ/cm^2^) radiation and treated with cannabidiol (CBD, 4 μM) in a three-dimensional culture model. Proteins IDs: P25786, P25788, P25789, P60900, proteasome subunit α type-1, -3, -4, -6; P20618, P49721, P49720, P28070, P28074, proteasome subunit β type-1, -2, -3, -4, -5. Statistical significances showed only for the whole complexes. x: statistically significant differences vs. non-treated cells, *p* < 0.05; y: statistically significant differences vs. CBD-treated cells, *p* < 0.05; a: statistically significant differences vs. UVA-irradiated cells, *p* < 0.05; b: statistically significant differences vs. UVB-irradiated cells, *p* < 0.05.

To evaluate which proteins were highly modified in keratinocytes following UVA/UVB irradiation and CBD treatment, we analyzed the q-value of individual proteins. The proteins with modified expression were grouped according to the functional complexes of which they are part. This analysis allowed us to determine the biological functions of the modified proteins. Figure 4, Figure 5 and Figure 6 show complexes containing proteins with the most significantly changed expression patterns. Both UVA and UVB radiation enhanced the level of proteins involved in the inflammatory response (TNFα/NFκB and IκBKB complexes). CBD—to varying degrees—prevented these changes, with particular counteraction in the case of cells exposed to UVA radiation (Figure 4). The same pattern of changes was observed in the expression of proteins involved in de novo protein biosynthesis (multisynthetase and ribosome complexes). An exception was glutamine-tRNA ligase (P47897), which was enhanced by CBD even more than UV radiation (Figure 5). Moreover, CBD enhanced the UV-induced expression of 20S proteasome subunits (Figure 6).

**Figure 7:**
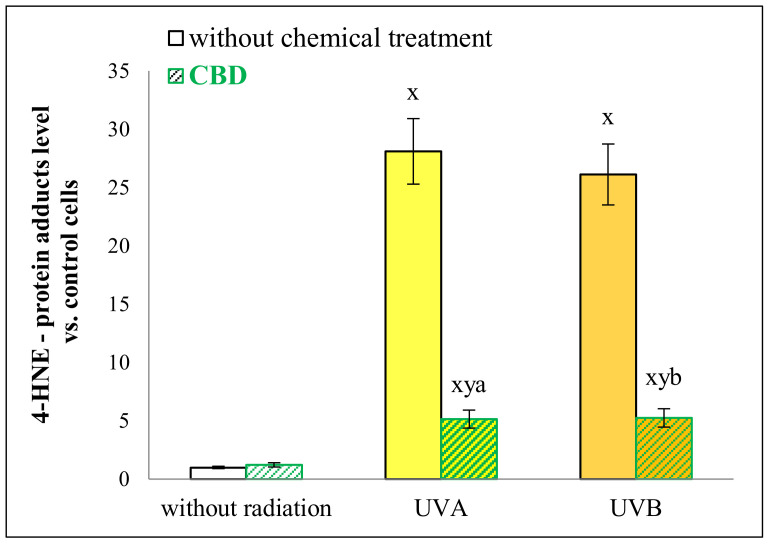
The level of 4-hydroxynonenal (4-HNE)-protein adducts in keratinocytes following UVA (30 J/cm^2^) or UVB (60 mJ/cm^2^) radiation and treated with cannabidiol (CBD, 4 μM) in a three-dimensional culture model. x: statistically significant differences vs. non-treated cells, *p* < 0.05; y: statistically significant differences vs. CBD-treated cells, *p* < 0.05; a: statistically significant differences vs. UVA-irradiated cells, *p* < 0.05; b: statistically significant differences vs. UVB-irradiated cells, *p* < 0.05.

**Figure 8:**
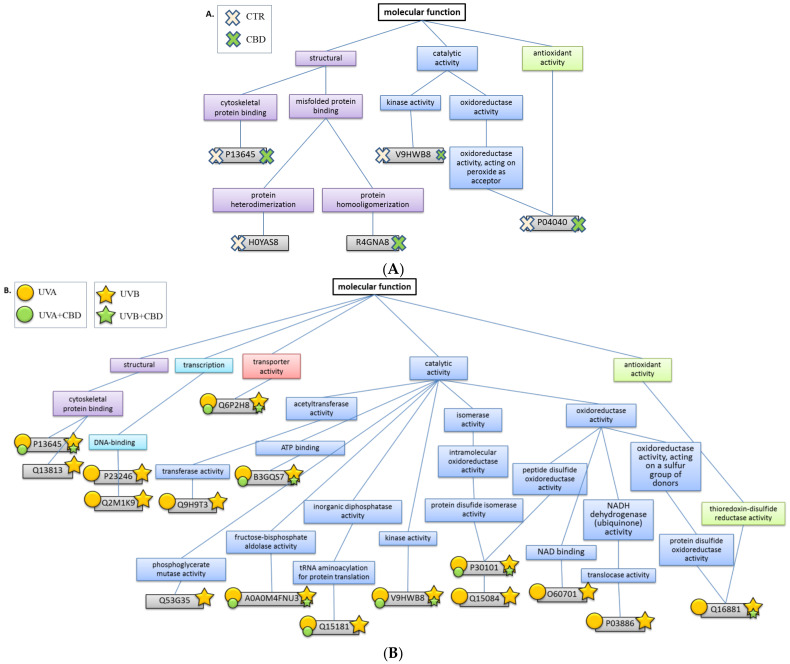
The biological function of proteins forming adducts with 4-hydroxynonenal (4-HNE) in keratinocytes treated in a three-dimensional culture model with cannabidiol (CBD, 4 μM) (**A**) and following UVA (30 J/cm^2^) or UVB (60 mJ/cm^2^) (**B**) radiation. The size of the stamps indicates the statistically significant differences between the estimated amount of the 4-HNE-protein adducts in the samples non-treated or treated with CBD.

Due to CBD’s antioxidant properties, we also expected that CBD treatment would significantly reduce lipid peroxidation levels, resulting in a decrease in lipid peroxidation products binding to proteins. Our findings demonstrate that CBD prevented the formation of adducts between proteins and one lipid peroxidation product-4-hydroxynonenal (4-HNE)-induced by UV radiation (Figure 7). Its protective action resulted in a five-fold reduction in the level of 4-HNE-protein adducts in keratinocytes following UVA or UVB irradiation. Furthermore, CBD not only led to a decrease in the levels of 4-HNE-protein adducts but, in the case of some proteins, completely prevented their formation following UVA and UVB exposure. These include proteins with oxidoreductase activity (UDP-glucose 6-dehydrogenase (O60701) and NADH-ubiquinone oxidoreductase (P03886)), acetyltransferase activity (elongator complex protein 3 (Q9H9T3)), and molecules involved in transcription processes (splicing factor, proline- and glutamine-rich (P23246) and zinc finger protein 423 (Q2M1K9)) (Figure 8).

## 3. Discussion

As a non-psychoactive component of cannabis, CBD is marketed as a treatment for various health conditions. Many individuals use CBD for a range of purposes, often without supervision or consultation with a specialist [25]. Moreover, the antioxidant and anti-inflammatory properties that can be beneficial for treating skin concerns like inflammation, dryness, and free radical damage has led to the growing trend of its use in skincare products [26]. To ensure CBD is used safely, it is necessary to identify its effects on the metabolism of the whole organism, tissues, and individual cells. Currently, the most comprehensive results are obtained by the omics approaches, including proteomics. Proteomics is the large-scale study of the structure and function of proteins in complex biological samples and shows a cross-section through the entire protein profile of the tested samples, as well as indicates changes in the structure of the molecules [27]. Such an approach allows to understand the complex nature of the organism; however, according to the abundance of high-weight proteins, including keratin, the detection and identification of the least abundant proteins may be impracticable [28].

To make our research more physiologically relevant, we analyzed the effect of CBD on the proteome of skin keratinocytes cultured in a 3D system. This 3D culture brings the experimental model closer to the epidermal conditions. As previously shown, the physiological metabolism of keratinocytes can only be simulated in vitro if the cells are provided with the appropriate nutrients and the structure for growth [29]. However, so far, the emphasis on stratified skin cellular structure necessary for proper skin cell functioning has been essential, mainly in the case of skin regeneration analysis [30]. Studies using multilayer/spheroid structures are crucial for understanding the cell-to-cell and cell-to-extracellular matrix interactions. Additionally, cell–cell and cell–environment communication also influence the cells’ response to xenobiotics appearing in their environment [31]. Dermatotoxicity tests using 3D cultures clearly show that the cell reaction in multilevel structures is significantly weaker than in the case of 2D cultures, and therefore, 3D systems may be more likely to reflect the physiological skin response [32,33]. In the case of CBD, previous data have shown that skin cells react differently depending on the culture model (2D or 3D) [19]. Importantly, CBD can penetrate deep into the skin layers [34], ensuring the treatment of all cells in a multilayer system.

Proteins IDs: A0A0M4FNU3, fructose-bisphosphate aldolase; B3GQS7, mitochondrial heat shock 60kD protein 1; H0YAS8, clusterin; O60701, UDP-glucose 6-dehydrogenase; P03886, NADH-ubiquinone oxidoreductase; P04040, catalase; P13645, keratin; P23246, splicing factor, proline- and glutamine-rich; P30101, protein disulfide-isomerase A3; Q13813, spectrin α chain; Q15084, protein disulfide-isomerase A6; Q15181, inorganic pyrophosphatase; Q16881, thioredoxin reductase; Q2M1K9, zinc finger protein 423; Q53G35, phosphoglycerate mutase; Q6P2H8, transmembrane protein 53; Q9H9T3, elongator complex protein 3; R4GNA8, protein p55; V9HWB8, pyruvate kinase.

Many studies show the various effects of CBD on cellular metabolism. Some indicate only the positive effects of CBD, such as increased proliferation and integrity of human brain endothelial cells through TRPV2 activation [35]. However, CBD may also induce autophagy in a TRPV2 and PI3K/AKT-dependent manner [36]. Another benefit of CBD is its cytoprotective effect, related to its interaction with specific receptors located on cell or nuclear membranes, such as CB2, GPR55, or PPARγ, and changes the expression of, for example, proteins involved in inflammatory processes [7]. As shown in this study, the anti-inflammatory action of CBD is also supported by the inhibition of the NFκB-dependent pathway (Figure 4). Other studies have shown that CBD can decrease NFκB levels under stress conditions [37]. The mechanism of this action is partially explained by the CBD-induced translocation of NFκB subunits to the nucleus [38], but also by IκBα degradation, as well as its decreased phosphorylation catalyzed by ERK1/2 and p38 MAPK [38,39]. Moreover, CBD is a potent inhibitor of the EGF/EGFR pathway in cancer cells [40], which also inhibits the activation of the proinflammatory NFκB pathway.

Previously published data showing CBD induces a decrease in NFκB, TNFα, and IκBKB levels in UV irradiated skin cells [9,41]. However, our results are also supported by the fact that CBD inhibits UV-induced expression of other proteins involved in NFκB activity, including heat shock proteins HSP90A and HSP90B, 60S ribosomal proteins, and cell cycle and apoptosis regulator protein 2 (Ccar2) (Figure 4). The fundamental roles of chaperones and ribosomes confirm the cytoprotective effect of CBD on UV irradiated skin cells based on its anti-inflammatory action and its influence on biosynthesis and folding of proinflammatory proteins. Moreover, one of the molecular functions of HSP90 is to provide the stability of DNA polymerase η and promote its nuclear accumulation in UV irradiated cells [42], ensuring DNA replication despite UV-induced damages. However, a high-level of UV-induced DNA damage leads to DNA mutations and their replication, which causes neoplastic processes. Therefore, the HSP90 inhibition results in a decrease in DNA polymerase η activity, which has been found to protect against cancer development [43,44]. Moreover, Ccar2 is also decreased by CBD, mainly in UVA-irradiated cells. Ccar2 is responsible for integrating transcript elongation with the regulation of alternative splicing, and when upregulated, it favors cancer development [45]. Therefore, our study provides evidence of additional anticarcinogenic properties of CBD in UVA-irradiated skin cells. However, the damages caused by UVB by an unknown mechanism do not allow this effect of CBD to be perceived in the cease of UVB-irradiated cells. That additionally indicates that UVB is more cancerogenic than UVA, and it is more difficult to prevent/reverse its effects [46].

The damage caused by UVA and UVB leads to the continuous synthesis of new proteins, which causes an uncontrolled accumulation of immature and dysfunctional molecules. A number of cytoprotective compounds (vitamin C, polyphenols, and melatonin) have been tested, and *de novo* protein biosynthesis inhibitors have been identified, which protect skin cells against such changes [47,48]. Our research also shows that CBD inhibits UV-induced de novo protein biosynthesis by decreasing the expression of proteins involved in the formation of multisynthetase complexes and complexes with ribosomal activity (Figure 5). Currently, there is no data on the effect of CBD on proteins involved in gene transcription and translation. However, considering the decrease in the level of CBD-induced microRNA [49], we suggest that this phytocannabinoid has a significant effect on gene expression and inhibiting the formation of active ribosomal complexes.

In UV irradiated skin cells, CBD not only regulates the level of protein synthesis and maturing, visible as the changes in multisynthetase and ribosome complex (Figure 5), but can also significantly modify their structure by, for example, influencing the formation of adducts with lipid peroxidation products (Figure 7). It has been previously shown that the model of cell culture has an impact on the Keap1/Nrf2/ARE pathway activation [31]. Moreover, earlier data indicate that CBD can directly bind proteins and influence their activity, as previously demonstrated for the Keap1 protein [9], a cytoplasmic inhibitor of the antioxidant factor Nrf2. The formation of the CBD-Keap1 adduct significantly contributes to Nrf2/ARE activation, which stimulates the antioxidant system. Proteomic analysis of its effect on cells also shows that CBD strongly activates the Nrf2/ARE pathway by interacting with the Nrf2 nuclear inhibitor Bach1 [10,50]. Changes in the structure of Bach1 reduce its possibility to compete with Nrf2 in DNA-binding, favoring the increased biosynthesis of cytoprotective proteins, including antioxidants [51]. The increased antioxidant capacity of cells induced by CBD is accompanied by a reduced ROS level, which reduces the opportunity for oxidative modifications of cell components, including phospholipids [9]. Earlier work indicated a reduction in the level of lipid peroxidation products and their interaction with proteins with adducts formation [19]. These studies confirm that CBD reduces the level of 4-HNE adducts with proteins (Figure 7). This finding is significant, as 4-HNE is one of the most reactive α,β-unsaturated aldehydes generated in the lipid peroxidation process and can inactivate most proteins [4]. As a result, such modifications change the properties of proteins, which is particularly important in the case of cytoprotective proteins induced under oxidative stress [5].

In the group of proteins modified by 4-HNE in the UVA/B irradiated keratinocytes, we identified NADH-ubiquinone oxidoreductase (complex I) as a major source of ROS in mitochondria (Figure 8) [52]. This enzyme is the first large protein complex of the respiratory chains and catalyzes the transfer of electrons from NADH to coenzyme Q10 with the simultaneous translocation of protons across the inner mitochondrial membrane. Any disturbances in NADH-ubiquinone oxidoreductase results in ROS generation and contributes to cellular oxidative stress, which is significantly induced in UV-irradiated cells [53]. To our knowledge, there is no evidence in the literature of an indirect impact of CBD or 4-HNE adduct formation on NADH-ubiquinone oxidoreductase activity. However, other cytoprotective compounds (metformin) that reduce 4-HNE levels inhibit these enzymes, a mechanism used in cancer immunotherapy [54].

Another protein protected by CBD against modification by 4-HNE is thioredoxin reductase (TrxR) (Figure 8), which is indirectly involved in antioxidant activity. TrxR expression is induced by Nrf2 under oxidative stress. However, 4-HNE adducts inhibit TrxR antioxidant activity [55,56]. This study found that, by preventing the formation of 4-HNE-TrxR adducts, CBD protects these enzymes against 4-HNE-induced inactivation. Additionally, other studies confirm that CBD increases TrxR activity in UV irradiated skin cells [9], indicating a robust cytoprotective effect of CBD. Moreover, the antioxidant activity of thioredoxin and the TrxR system is based on the availability and possible modification of cysteine thiol groups. The enzyme responsible for the formation and breakage of disulfide bonds between cysteine residues within proteins is protein disulfide-isomerase [57], the expression of which, similar to TrxR, is under the control of Nrf2. The level of this enzyme in cells following UV irradiation has been increased [58]. However, its structural modification under oxidative stress prevents protein disulfide-isomerase activation, contributing to endoplasmic reticulum stress and apoptosis [59]. CBD significantly reduces protein disulfide-isomerase modification by 4-HNE (Figure 8), thereby contributing to protein disulfide-isomerase activation under oxidative stress, ensuring proper cysteine residue conformation within proteins. However, this effect is observed in the case of protein disulfide-isomerase A6 more strongly in the case of A3 form. This may be related to the structure of these proteins; in the A6 form, there are about 40% fewer amino acid residues susceptible to adduct formation with 4-HNE (lysine, histidine, cysteine) than in A3 form [60], so their protection may be more effective. Disulfide isomerase A6 also exhibits a wider range of functions. Despite its role in rearranging S-S bonds, the A6 form may additionally act as a chaperone and inhibit aggregation of misfolded proteins [61], which has not been described for disulfide isomerase A3. Moreover, disulfide isomerase A6, unlike A3, also has been found in the membrane fraction [62], making it more susceptible to the protective effects of lipophilic CBD. On the other hand, a similar cytoprotective action of CBD against the formation of 4-HNE-protein disulfide-isomerase adducts was previously observed for adenosylmethionine [62].

CBD strongly prevents the formation of UV-induced 4-HNE adducts with other catalytic proteins (Figure 8). These primarily include proteins participating in signal transduction and regulation of cell function, such as kinases, transporters, and transcription regulators. CBD-induced reduction of 4-HNE adduct formation protects against structural changes. As a result, many of these proteins retain their endogenous activity, necessary for normal cell function and the prevention of neoplastic transformation [4].

Regardless of the effect on protein biosynthesis, structural modification, and biological functions through the formation of 4-HNE-protein adducts, exposure of skin cells to UVA, and UVB radiation undoubtedly leads to intracellular redox imbalance and protein oxidation [3]. The formation of oxidatively modified proteins and their impaired proteolytic degradation under the influence of UV radiation causes the proteins to accumulate. This buildup disrupts cellular metabolism, leading to cell aging and neoplastic transformation [63,64]. Thus, CBD-induced stimulation of the 20S proteasome (Figure 6) is particularly important, as it is primarily responsible for the degradation of modified proteins [65]. However, also, in this case, CBD action is more visible in cells irradiated with UVA, while UVB-irradiated cells have such high levels of damaged proteins [46] that impaired proteasomal system cannot be effectively stimulated by CBD. Other proteomic studies have previously shown that CBD can induce the expression of proteins involved in various metabolic pathways, including inhibition of proteolytic degradation [66]. Consequently, CBD plays a role in monitoring the breakdown of damaged proteins and preventing their accumulation.

## 4. Materials and Methods

### 4.1. Cell Culture and Treatment

Human keratinocytes (CDD 1102 KERTr) were obtained from American Type Culture Collection and cultured according to the protocol as described previously [67]. The 3D culture was carried out in AlgiMatrix plates (Life Technologies, California, USA) [68]. To observe the effect of CBD on UV irradiated cells, keratinocytes were first exposed to UVA or UVB radiation (Bio-Link Crosslinker BLX 312/365; Vilber Lourmat, Germany). Irradiation was carried out using the following doses: UVA—30 J/cm^2^ and UVB—60 mJ/cm^2^, which caused, in both cases, a reduction of cell viability to 70% measured by MTT in a 2D model [3]. It has also previously been shown that the doses used are sufficient to induce statistically significant changes in the protein profile [67]. After four days of 3D culture, cells intended for UV exposure were washed three times with warm PBS (37 °C) to remove the medium from the AlgiMatrix. Cells were exposed to UV radiation in cold PBS (4 °C) to avoid heat stress and oxidation of the medium components. The cells were irradiated at a distance of 15 cm from the 6 lamps (6 W each), which corresponds to 4.2 mW/cm^2^ and 4.08 mW/cm^2^, respectively, for UVA (365 nm) and UVB (312 nm). Following irradiation, cells were incubated for 24 h in media supplemented with 4 µM of CBD in 0.1% ethanol [16]. Control cells were cultured parallel in media containing 0.1% ethanol. Following incubation, cells were collected using AlgiMatrix^®^ Dissolving Buffer, dissolved in lysis buffer (10 mM Tris-HCl pH 7.4, 1 mM EDTA, 1% Triton X-100, 0.1% SDS) and lysed through sonification on ice. The total protein concentration in the lysates was measured using a Bradford assay [69].

### 4.2. Proteomic Analysis

**Figure 9:**
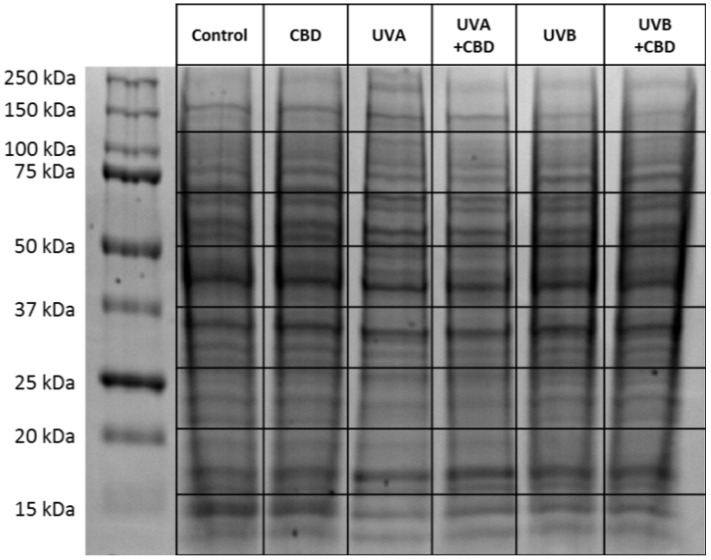
SDS–PAGE separation and staining with Coomassie brilliant blue R-250 of proteins from control keratinocytes and irradiated with UVA (30 J/cm^2^), UVB (60 mJ/cm^2^) or/and treated with cannabidiol (CBD, 4 μM) in a three-dimensional (3D) culture model. The grid indicates the borders of the protein migration zones. Original photo of the gel is added in Supplementary Figure S1.

Cell lysates were separated on 10% Tris-Glycine SDS–PAGE gels and stained overnight with Coomassie Brilliant Blue R-250. Entire lanes were cut out of the gel, sliced into eight sections (Figure 9), and in-gel digested overnight with sequencing grade trypsin (Promega, Madison, WI, USA). The obtained peptide mixture was extracted from the gel, dried, and dissolved in 50 µL ACN + 0.1% formic acid (FA) [70]. A total of 5 µL of this mixture were separated using an Ultimate 3000 (Dionex, Idstein, Germany) onto a 150 mm × 75 µm PepMap RSLC capillary analytical C18 column with 2 μm particle size (Dionex, LC Packings). The peptides eluted from the column were analyzed using a Q Exactive HF mass spectrometer in a positive mode (Thermo Fisher Scientific, Bremen, Germany). The details of the protein separation and peptide analysis by LC–MS/MS have been shown previously [71].

### 4.3. Protein Identification and Label-Free Quantification

The raw data generated from LC–MS/MS analysis were processed using Proteome Discoverer 2.0 (Thermo Fisher Scientific, Bremen, Germany). Input data were searched against the UniProtKB-SwissProt database (taxonomy: *Homo sapiens*, release 2019–08). Parameters of peptide mass tolerance set to 10 ppm, MS/MS mass tolerance set to 0.02 Da, and up to two allowed missed cleavages were used for protein identification. Cysteine carbamidomethylation and carboxymethylation, methionine oxidation, and 4-HNE—cysteine/lysine/histidine adduct formation were set as dynamic modifications [71,72,73]. Only proteins with at least three identified peptides longer than six amino acid residues and at least two unique peptides were selected for further analysis. The protein quantification was carried out based on the corresponding peak area analysis. The level of 4-HNE-protein adducts was estimated based on the peak intensity of peptides modified by 4-HNE. Results were validated by ELISA using specific anti-4-HNE-His murine monoclonal antibodies (clone 4-HNE 1g4) and goat anti-mouse antibody (Dako, Carpinteria, CA, USA) (data not shown).

### 4.4. Statistical Analysis

Analysis of keratinocytes under each experimental condition was performed in three independent experiments. All data were normalized by the total sample intensity available in the MetaboAnalyst 4.0 software (http://www.metaboanalyst.ca) [74]. Using the same software, the missing values were replaced by half of the minimum positive values detected in the original data.

Results from individual protein label-free quantification were a log and Z-score transformed using RStudio software (R version 3.6.2 (2019-12-12)) [75]. Principal component analysis (PCA) (carried out separately for UVA and UVB treated cell) and clustering of all samples was performed with the R built-in function in MetaboAnalyst 4.0 software (http://www.metaboanalyst.ca) [76]. PCA plots were presented with 95% confidence regions for groups. Hierarchical clustering dendrogram was created basing on measure squared Euclidean distance, using as an algorithm Ward linkage. Complex analyses were carried out using ComplexBrowser (http://computproteomics.bmb.sdu.dk/Apps/Complex Browser/) [77] with software default settings: (q-value threshold at 0.05; fold change threshold at 1.2; noise threshold for summary at 0.5, and CORUM as a database for analysis). The biological function of proteins was analyzed using generic gene ontology (GO) term finder (GO-TermFinder) version 0.86 (https://go.princeton.edu/cgi-bin/GOTermFinder) [78].

## 5. Conclusions

In conclusion, we have demonstrated that CBD shows a series of positive actions that stimulate skin cells to prevent the harmful effects of UVA and UVB radiation. This includes antioxidant and anti-inflammatory activity, regulation of protein biosynthesis and degradation, and control of enzyme activity by structural modification. However, further research is required to elucidate its long-term use in epidermal cells.

## Data Availability

The data presented in this study are available in Supplementary Table S1.

## Supplementary Materials

The names and abundance of proteins identified and analyzed in this study are available online at https://www.mdpi.com/1422-0067/22/3/1501/s1, Table S1: The names and abundance of proteins identified in the keratinocytes cultured in a three-dimensional culture model and treated with cannabidiol (CBD, 4 μM) following UVA (30 J/cm^2^) or UVB (60 mJ/cm^2^) radiation. Figure S1: SDS–PAGE separation and staining with Coomassie Brilliant Blue R-250 of proteins from control keratinocytes and irradiated with UVA (30 J/cm^2^), UVB (60 mJ/cm^2^) or/and treated with cannabidiol (CBD, 4 μM) in a three-dimensional (3D) culture model.
