# Supplementary material for: The Effect of Cannabidiol on UV-Induced Changes in Intracellular Signaling of 3D-Cultured Skin Keratinocytes
Source: Int J Mol Sci. 2021 Feb 2;22(3):1501. doi: 10.3390/ijms22031501 (PMC7867360; doi:10.3390/ijms22031501)

Supplementary Figure S1

SDS-PAGE separation and staining with Coomassie Brilliant Blue R-250 of proteins from control keratinocytes and irradiated with UVA (30 J/cm^2), UVB (60 mJ/cm^2) or/and treated with cannabidiol (CBD, 4 µM) in a three-dimensional (3D) culture model.

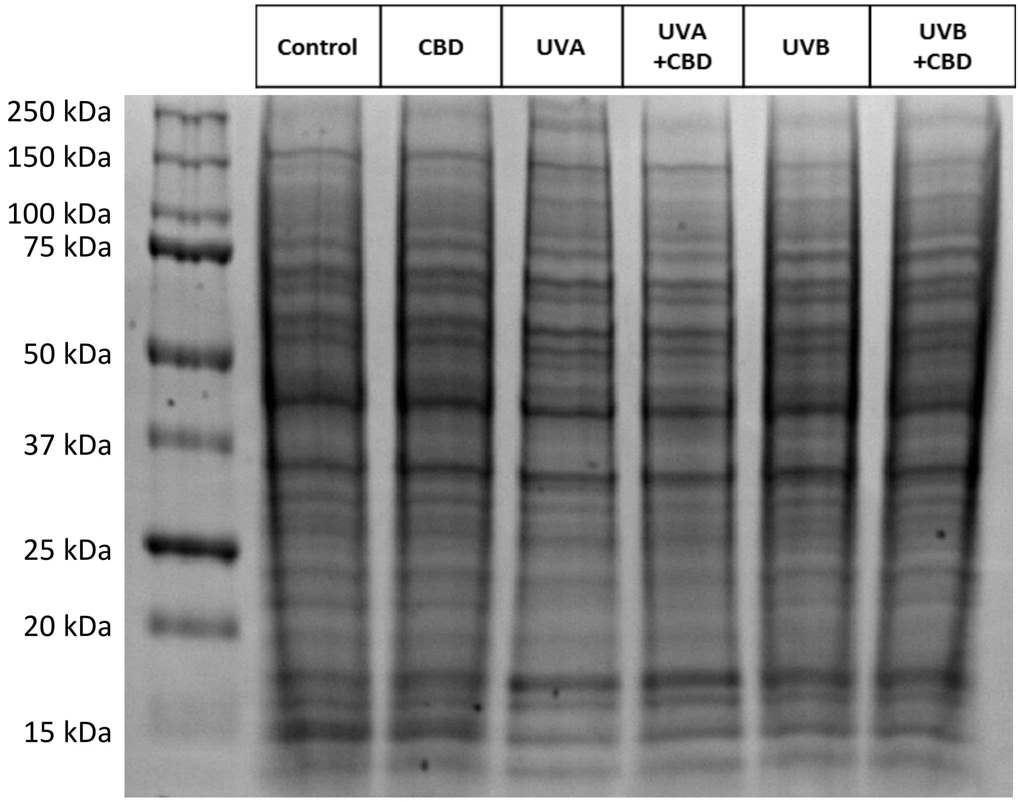

Supplement: Supplementary file 1 [file ijms-22-01501-s001.zip › Supplementary Figure S1.pdf]
