# Supplementary material for: The Effect of Cannabidiol on UV-Induced Changes in Intracellular Signaling of 3D-Cultured Skin Keratinocytes
Source: Int J Mol Sci. 2021 Feb 2;22(3):1501. doi: 10.3390/ijms22031501 (PMC7867360; doi:10.3390/ijms22031501)
Supplement: Supplementary file 1 [file ijms-22-01501-s001.zip › Supplementary Table S1.pdf]

Supplementary table S1. The names and abundance of proteins identified in the keratinocytes cultured in a three-dimensional culture model and treated with cannabidiol (CBD, 4 μM) following UVA (30 J/cm^2) or UVB (60 mJ/cm^2) (B) radiation.
Data analyzed using Metaboanalyst with R version 3.6.2. Abbreviations: Ctr, control; CBD, cannabidiol, b.t., below threshold.

ID	Protein	Ctr1	Ctr2	Ctr3	UVA1	UVA2	UVA3	UVB1	UVB2	UVB3	CBD1	CBD2	CBD3	UVA + CBD1	UVA + CBD2	UVA + CBD3	UVB + CBD1	UVB + CBD2	UVB + CBD3
A0A024QZN4	Vinculin	b.t.	b.t.	b.t.	4,30E+06	4,30E+06	5,59E+06	b.t.	b.t.	b.t.	1,40E+06	2,10E+06	1,40E+06	8,46E+06	9,40E+06	8,93E+06	3,90E+06	1,00E+07	6,70E+06
A0A024QZN9	Voltage-dependent anion channel 2	b.t.	b.t.	b.t.	b.t.	b.t.	b.t.	7,05E+05	5,88E+05	4,70E+05	b.t.	b.t.	b.t.	6,93E+06	7,70E+06	7,32E+06	1,50E+07	1,35E+07	1,08E+07
A0A024QZZ7	Histone H2B	b.t.	b.t.	b.t.	b.t.	b.t.	b.t.	b.t.	b.t.	b.t.	2,80E+06	4,20E+06	2,80E+06	1,08E+07	1,20E+07	1,14E+07	b.t.	b.t.	b.t.
A0A024R172	B4 12-hydroxydehydrogenase	6,60E+05	9,90E+05	6,60E+05	8,28E+06	7,59E+06	6,90E+06	3,90E+06	3,25E+06	2,60E+06	8,60E+06	1,29E+07	8,60E+06	1,98E+07	2,20E+07	2,09E+07	1,80E+07	1,62E+07	1,20E+06
A0A024R1A3	Ubiquitin-activating enzyme E1	2,10E+06	1,30E+06	2,90E+06	1,67E+07	1,80E+07	1,10E+07	4,50E+06	3,75E+06	3,00E+06	6,50E+06	5,60E+06	6,30E+06	4,86E+07	5,40E+07	5,13E+07	2,30E+07	4,00E+07	3,00E+07
A0A024R1K7	Tyrosine 3-monooxygenase/tryptophan 5-monooxygenase activation	b.t.	b.t.	b.t.	3,09E+06	4,90E+06	9,20E+05	b.t.	b.t.	b.t.	6,50E+06	9,75E+06	6,50E+06	b.t.	b.t.	b.t.	1,10E+07	9,90E+06	1,10E+06
A0A024R1N4	X-ray repair complementing defective	b.t.	b.t.	b.t.	7,78E+06	8,70E+06	4,90E+06	2,10E+06	1,75E+06	1,40E+06	1,20E+06	9,90E+05	1,40E+06	1,62E+07	1,80E+07	1,71E+07	2,60E+07	1,10E+07	1,80E+07
A0A024R231	Guanine deaminase	2,00E+06	2,00E+06	3,00E+06	1,42E+07	2,20E+07	4,60E+06	5,25E+06	4,38E+06	3,50E+06	2,40E+06	3,50E+06	1,70E+06	4,32E+07	4,80E+07	4,56E+07	2,40E+07	4,20E+07	3,40E+07
A0A024R2G7	0	b.t.	b.t.	b.t.	b.t.	b.t.	b.t.	b.t.	b.t.	b.t.	3,00E+06	4,50E+06	3,00E+06	5,67E+06	6,30E+06	5,99E+06	2,30E+06	2,07E+06	1,66E+06
A0A024R2P0	40S ribosomal protein S1	b.t.	b.t.	b.t.	7,34E+06	6,70E+06	5,70E+06	4,35E+06	3,63E+06	2,90E+06	6,70E+06	7,80E+06	7,20E+06	4,86E+07	5,40E+07	5,13E+07	3,00E+07	2,70E+07	3,80E+07
A0A024R2Q4	Ribosomal protein L15	b.t.	b.t.	b.t.	1,13E+06	1,03E+06	9,40E+05	b.t.	b.t.	b.t.	3,80E+06	5,70E+06	3,80E+06	1,62E+06	1,80E+06	1,71E+06	8,20E+06	7,38E+06	5,90E+06
A0A024R3W7	Eukaryotic translation elongation factor 1 beta 2	b.t.	b.t.	b.t.	b.t.	b.t.	b.t.	b.t.	b.t.	b.t.	2,60E+05	2,60E+05	3,90E+05	b.t.	b.t.	b.t.	9,50E+06	8,55E+06	6,84E+06
A0A024R454	Carbamoyl-phosphate synthetase 1	3,35E+06	5,00E+06	1,70E+06	4,64E+07	5,50E+07	2,70E+07	1,92E+06	8,40E+05	1,50E+06	5,00E+06	4,40E+06	1,90E+07	7,38E+07	8,20E+07	7,79E+07	2,70E+07	2,10E+07	4,90E+07
A0A024R4Q8	Ribosomal protein S5	b.t.	b.t.	b.t.	b.t.	b.t.	b.t.	b.t.	b.t.	b.t.	b.t.	b.t.	b.t.	3,06E+06	3,40E+06	3,23E+06	1,60E+07	1,44E+07	1,15E+07
A0A024R4U3	Tubulin tyrosine ligase-like family. member 12	b.t.	b.t.	b.t.	2,30E+06	2,30E+06	2,99E+06	b.t.	b.t.	b.t.	b.t.	b.t.	b.t.	b.t.	b.t.	b.t.	4,32E+06	3,89E+06	3,60E+06
A0A024R5Z8	RAB11A. member RAS oncogene family	b.t.	b.t.	b.t.	9,72E+06	8,91E+06	8,10E+06	b.t.	b.t.	b.t.	b.t.	b.t.	b.t.	b.t.	b.t.	b.t.	2,40E+05	2,40E+05	1,92E+05
A0A024R652	Methylenetetrahydrofolate dehydrogenase (NADP+ dependent) 1	b.t.	b.t.	b.t.	2,52E+06	2,31E+06	2,10E+06	b.t.	b.t.	b.t.	1,36E+06	7,10E+05	2,00E+06	1,17E+07	1,30E+07	1,24E+07	1,10E+07	1,10E+07	1,00E+07
A0A024R6C9	Dihydrolipoamide S-succinyltransferase	b.t.	b.t.	b.t.	b.t.	b.t.	b.t.	b.t.	b.t.	b.t.	1,60E+06	2,40E+06	1,60E+06	7,74E+06	8,60E+06	8,17E+06	9,60E+06	8,64E+06	6,91E+06
A0A024R6S1	DnaJ (Hsp40) homolog	b.t.	b.t.	b.t.	b.t.	b.t.	b.t.	b.t.	b.t.	b.t.	b.t.	b.t.	b.t.	1,89E+06	2,10E+06	2,00E+06	b.t.	b.t.	b.t.
A0A024R718	Pre-B-cell colony enhancing factor 1	1,10E+06	1,65E+06	1,10E+06	1,40E+06	2,90E+07	1,70E+07	6,05E+06	1,50E+06	5,30E+06	1,30E+07	2,00E+07	5,30E+07	1,80E+08	2,00E+08	1,90E+08	6,30E+07	8,50E+07	1,30E+08
A0A024R7T3	Heterogeneous nuclear ribonucleoprotein F	b.t.	b.t.	b.t.	6,84E+06	6,27E+06	5,70E+06	1,50E+06	1,25E+06	1,00E+06	1,30E+07	1,95E+07	1,30E+07	9,00E+06	1,00E+07	9,50E+06	b.t.	b.t.	b.t.
A0A024R814	Ribosomal protein L7	b.t.	b.t.	b.t.	4,32E+06	3,96E+06	3,60E+06	2,20E+06	1,20E+06	1,60E+06	7,30E+06	1,10E+07	7,30E+06	1,53E+07	1,70E+07	1,62E+07	2,20E+07	1,98E+07	1,58E+07
A0A024R872	Chromosome 9 open reading frame 88	b.t.	b.t.	b.t.	2,65E+06	3,20E+06	1,50E+06	b.t.	b.t.	b.t.	1,70E+06	1,50E+06	1,90E+06	7,47E+06	8,30E+06	7,89E+06	4,20E+06	3,78E+06	3,60E+06
A0A024R895	SET translocation	b.t.	b.t.	b.t.	7,32E+06	6,71E+06	6,10E+06	9,45E+05	7,88E+05	6,30E+05	b.t.	b.t.	b.t.	1,17E+07	1,30E+07	1,24E+07	1,20E+07	1,08E+07	8,64E+06
A0A024R8W0	DEAD (Asp-Glu-Ala-Asp) box polypeptide 48	b.t.	b.t.	b.t.	5,20E+06	5,20E+06	6,76E+06	b.t.	b.t.	b.t.	b.t.	b.t.	b.t.	5,13E+06	5,70E+06	5,42E+06	6,90E+06	1,10E+07	5,50E+06
A0A024R9E2	Poly(A) binding protein, cytoplasmic 1	b.t.	b.t.	b.t.	b.t.	b.t.	b.t.	b.t.	b.t.	b.t.	2,30E+06	2,30E+06	3,45E+06	1,44E+07	1,60E+07	1,52E+07	5,90E+06	7,70E+06	1,50E+07
A0A024RAE4	Cell division cycle 42	b.t.	b.t.	b.t.	8,76E+06	8,03E+06	7,30E+06	2,85E+06	2,38E+06	1,90E+06	b.t.	b.t.	b.t.	b.t.	b.t.	b.t.	b.t.	b.t.	b.t.
A0A024RAH8	DEAD (Asp-Glu-Ala-Asp) box polypeptide 18	b.t.	b.t.	b.t.	9,12E+05	8,36E+05	7,60E+05	b.t.	b.t.	b.t.	b.t.	b.t.	b.t.	b.t.	b.t.	b.t.	2,00E+06	1,80E+06	1,44E+06
A0A024RAM0	Transportin 1	3,00E+06	3,00E+06	4,50E+06	4,68E+06	4,29E+06	3,90E+06	b.t.	b.t.	b.t.	4,90E+06	7,35E+06	4,90E+06	1,80E+07	2,00E+07	1,90E+07	8,10E+06	7,29E+06	1,20E+07
A0A024RAM2	Glutaredoxin (Thioltransferase)	b.t.	b.t.	b.t.	3,48E+06	3,19E+06	2,90E+06	6,00E+06	5,00E+06	4,00E+06	8,30E+06	8,30E+06	1,25E+07	8,91E+06	9,90E+06	9,41E+06	2,10E+07	1,89E+07	1,51E+07
A0A024RB41	HCG2016482	b.t.	b.t.	b.t.	b.t.	b.t.	b.t.	b.t.	b.t.	b.t.	b.t.	b.t.	b.t.	1,17E+07	1,30E+07	1,24E+07	3,40E+06	3,06E+06	2,45E+06
A0A024RB85	Proliferation-associated 2G4	b.t.	b.t.	b.t.	b.t.	b.t.	b.t.	b.t.	b.t.	b.t.	1,50E+06	1,50E+06	2,25E+06	1,17E+07	1,30E+07	1,24E+07	1,00E+07	9,00E+06	1,10E+07
A0A024RB87	RAP1B	b.t.	b.t.	b.t.	b.t.	b.t.	b.t.	b.t.	b.t.	b.t.	b.t.	b.t.	b.t.	5,67E+06	6,30E+06	5,99E+06	3,00E+06	2,70E+06	2,16E+06
A0A024RB99	Serine hydroxymethyltransferase	b.t.	b.t.	b.t.	2,88E+06	2,64E+06	2,40E+06	1,98E+06	9,60E+05	1,50E+06	2,75E+06	1,40E+06	4,10E+06	2,16E+07	2,40E+07	2,28E+07	1,30E+07	4,10E+06	1,40E+07
A0A024RBH2	Cytoskeleton-associated protein 4	b.t.	b.t.	b.t.	b.t.	b.t.	b.t.	b.t.	b.t.	b.t.	b.t.	b.t.	b.t.	2,88E+06	3,20E+06	3,04E+06	4,40E+06	3,96E+06	1,00E+07
A0A024RBS1	GCN1 general control of amino-acid synthesis 1	b.t.	b.t.	b.t.	b.t.	b.t.	b.t.	b.t.	b.t.	b.t.	b.t.	b.t.	b.t.	b.t.	b.t.	b.t.	2,90E+06	2,61E+06	2,09E+06
A0A024RC46	HCG2020860	2,10E+06	2,10E+06	3,15E+06	7,80E+06	7,80E+06	1,01E+07	4,20E+06	3,50E+06	2,80E+06	1,40E+07	2,10E+07	1,40E+07	5,40E+07	6,00E+07	5,70E+07	2,60E+07	1,20E+06	1,10E+06
A0A024RC87	Ribonuclease/angiogenin inhibitor 1	b.t.	b.t.	b.t.	5,40E+06	4,95E+06	4,50E+06	b.t.	b.t.	b.t.	1,50E+06	1,50E+06	2,25E+06	6,93E+06	7,70E+06	7,32E+06	3,40E+06	3,06E+06	1,10E+07
A0A024RCN6	Valyl-tRNA synthetase	b.t.	b.t.	b.t.	3,91E+06	3,90E+06	2,80E+06	b.t.	b.t.	b.t.	b.t.	b.t.	b.t.	b.t.	b.t.	b.t.	4,20E+06	6,90E+06	2,30E+06
A0A024RD93	Phosphoribosylaminoimidazole carboxylase	b.t.	b.t.	b.t.	b.t.	b.t.	b.t.	1,50E+06	1,25E+06	1,00E+06	4,80E+05	4,80E+05	7,20E+05	6,84E+06	7,60E+06	7,22E+06	2,70E+06	2,43E+06	7,90E+06
A0A024RDF4	Heterogeneous nuclear ribonucleoprotein D	b.t.	b.t.	b.t.	6,96E+06	6,38E+06	5,80E+06	5,10E+06	4,25E+06	3,40E+06	3,45E+06	1,90E+06	5,00E+06	2,79E+07	3,10E+07	2,95E+07	1,70E+07	1,53E+07	1,80E+06
A0A024RDQ0	Heat shock 105kDa/110kDa protein 1	b.t.	b.t.	b.t.	6,10E+06	6,10E+06	7,93E+06	b.t.	b.t.	b.t.	1,30E+06	1,30E+06	1,95E+06	b.t.	b.t.	b.t.	4,40E+06	3,96E+06	6,00E+06
A0A024RDS1	Heat shock 105kDa/110kDa protein 2	b.t.	b.t.	b.t.	6,10E+06	6,10E+06	7,93E+06	b.t.	b.t.	b.t.	1,30E+06	1,30E+06	1,95E+06	b.t.	b.t.	b.t.	4,40E+06	3,96E+06	6,00E+06
A0A024RDY0	RAN binding protein 5	b.t.	b.t.	b.t.	6,54E+06	9,30E+06	2,70E+06	b.t.	b.t.	b.t.	b.t.	b.t.	b.t.	1,26E+07	1,40E+07	1,33E+07	3,87E+06	2,00E+06	4,10E+06
A0A075B716	40S ribosomal protein S17	b.t.	b.t.	b.t.	b.t.	b.t.	b.t.	b.t.	b.t.	b.t.	b.t.	b.t.	b.t.	7,38E+06	8,20E+06	7,79E+06	1,30E+07	1,17E+07	9,36E+06
A0A087WZV1	Heterogeneous nuclear ribonucleoprotein A/B	2,90E+06	2,90E+06	4,35E+06	7,16E+06	6,20E+06	5,80E+06	5,10E+06	4,25E+06	3,40E+06	1,80E+06	2,70E+06	1,80E+06	2,34E+07	2,60E+07	2,47E+07	1,80E+07	1,62E+07	2,90E+06
A0A087X1N8	Serpin B6	b.t.	b.t.	b.t.	b.t.	b.t.	b.t.	8,70E+05	7,25E+05	5,80E+05	b.t.	b.t.	b.t.	4,50E+06	5,00E+06	4,75E+06	2,30E+06	2,07E+06	3,20E+06
A0A087X1Z3	Proteasome activator complex subunit 2	b.t.	b.t.	b.t.	6,84E+05	6,27E+05	5,70E+05	b.t.	b.t.	b.t.	2,60E+06	1,70E+06	3,50E+06	5,22E+06	5,80E+06	5,51E+06	9,30E+06	8,37E+06	6,70E+06
A0A087X2D0	Serine/arginine-rich-splicing factor 3	b.t.	b.t.	b.t.	b.t.	b.t.	b.t.	b.t.	b.t.	b.t.	b.t.	b.t.	b.t.	1,62E+07	1,80E+07	1,71E+07	3,90E+07	3,51E+07	2,81E+07
A0A090N8G0	Glycyl-tRNA synthetase	1,50E+06	2,25E+06	1,50E+06	6,09E+06	9,80E+06	1,70E+06	3,30E+06	2,75E+06	2,20E+06	3,15E+06	1,90E+06	4,40E+06	1,35E+07	1,50E+07	1,43E+07	9,40E+06	1,10E+07	1,50E+07
A0A0A0MR02	Voltage-dependent anion-selective channel protein 2	b.t.	b.t.	b.t.	b.t.	b.t.	b.t.	7,05E+05	5,88E+05	4,70E+05	b.t.	b.t.	b.t.	6,93E+06	7,70E+06	7,32E+06	1,50E+07	1,35E+07	1,08E+07
A0A0A0MSS8	Aldo-keto reductase family 1 member C3	b.t.	b.t.	b.t.	6,22E+06	6,70E+06	4,10E+06	2,25E+06	1,30E+06	1,60E+06	1,79E+06	4,80E+05	3,10E+06	2,70E+07	3,00E+07	2,85E+07	1,60E+07	1,44E+07	1,50E+06
A0A0C4DGQ5	Calpain small subunit 1	b.t.	b.t.	b.t.	1,13E+06	1,03E+06	9,40E+05	b.t.	b.t.	b.t.	b.t.	b.t.	b.t.	1,35E+07	1,50E+07	1,43E+07	4,60E+06	4,14E+06	3,31E+06
A0A0D9SEM4	Serine/arginine-rich-splicing factor 4a	b.t.	b.t.	b.t.	b.t.	b.t.	b.t.	b.t.	b.t.	b.t.	b.t.	b.t.	b.t.	1,08E+07	1,20E+07	1,14E+07	5,40E+06	4,86E+06	3,89E+06
A0A0D9SF53	ATP-dependent RNA helicase DDX3X	b.t.	b.t.	b.t.	3,96E+06	3,63E+06	3,30E+06	b.t.	b.t.	b.t.	1,40E+06	1,40E+06	2,10E+06	1,71E+07	1,90E+07	1,81E+07	2,90E+06	2,61E+06	1,30E+05
A0A0K0K1K7	6-phosphogluconolactonase	b.t.	b.t.	b.t.	b.t.	b.t.	b.t.	b.t.	b.t.	b.t.	b.t.	b.t.	b.t.	1,44E+07	1,60E+07	1,52E+07	8,50E+06	7,65E+06	6,12E+06
A0A0M4FEM1	Alpha-1.4 glucan phosphorylase	b.t.	b.t.	b.t.	b.t.	b.t.	b.t.	b.t.	b.t.	b.t.	b.t.	b.t.	b.t.	b.t.	b.t.	b.t.	3,30E+06	2,97E+06	2,38E+06
A0A0M4FNU3	Fructose-bisphosphate aldolase A	1,45E+07	1,70E+07	1,20E+07	7,30E+05	8,30E+07	8,80E+07	1,42E+07	6,30E+06	1,10E+07	5,10E+07	1,80E+07	4,00E+07	3,60E+08	4,00E+08	3,80E+08	2,90E+08	1,20E+08	6,20E+07
A0A0S2Z3K9	V-crk sarcoma virus CT10 oncogene-like protein	b.t.	b.t.	b.t.	b.t.	b.t.	b.t.	b.t.	b.t.	b.t.	b.t.	b.t.	b.t.	b.t.	b.t.	b.t.	4,40E+06	3,96E+06	3,17E+06
A0A0S2Z410	Hydroxysteroid dehydrogenase 10 isoform 1	b.t.	b.t.	b.t.	b.t.	b.t.	b.t.	b.t.	b.t.	b.t.	1,00E+07	1,50E+07	1,00E+07	8,91E+06	9,90E+06	9,41E+06	2,20E+07	1,98E+07	1,58E+07
A0A0S2Z471	Creatine kinase brain isoform 2	b.t.	b.t.	b.t.	b.t.	b.t.	b.t.	b.t.	b.t.	b.t.	1,80E+06	2,70E+06	1,80E+06	7,92E+06	8,80E+06	8,36E+06	2,40E+06	2,16E+06	5,20E+06

A0A0S2Z4G4	Tropomyosin 3 isoform 1	b.t.	b.t.	b.t.	b.t.	b.t.	b.t.	b.t.	b.t.	b.t.	1,35E+06	5,90E+05	2,10E+06	9,00E+06	1,00E+07	9,50E+06	1,60E+07	1,44E+07	1,15E+07
A0A0S2Z4I4	Tropomyosin 3 isoform 3	b.t.	b.t.	b.t.	b.t.	b.t.	b.t.	b.t.	b.t.	b.t.	1,35E+06	5,90E+05	2,10E+06	9,00E+06	1,00E+07	9,50E+06	1,60E+07	1,44E+07	1,15E+07
A0A0S2Z4J1	Hydroxysteroid (17-beta) dehydrogenase 4	b.t.	b.t.	b.t.	4,20E+06	4,20E+06	5,46E+06	b.t.	b.t.	b.t.	b.t.	b.t.	b.t.	8,46E+06	9,40E+06	8,93E+06	1,60E+06	1,44E+06	1,15E+06
A0JLQ5	BXDC2 protein	b.t.	b.t.	b.t.	1,20E+06	1,20E+06	1,56E+06	b.t.	b.t.	b.t.	b.t.	b.t.	b.t.	6,66E+06	7,40E+06	7,03E+06	7,20E+06	6,48E+06	5,18E+06
A0PJ87	RSL1D1 protein	b.t.	b.t.	b.t.	2,26E+06	1,30E+06	2,30E+06	2,40E+06	2,00E+06	1,60E+06	b.t.	b.t.	b.t.	7,56E+06	8,40E+06	7,98E+06	4,10E+06	3,69E+06	4,00E+05
A3R0T7	Histone H1e	b.t.	b.t.	b.t.	b.t.	b.t.	b.t.	b.t.	b.t.	b.t.	1,40E+07	2,10E+07	1,40E+07	3,33E+07	3,70E+07	3,52E+07	3,10E+07	2,79E+07	2,23E+07
A3R0T8	Histone 1	b.t.	b.t.	b.t.	b.t.	b.t.	b.t.	b.t.	b.t.	b.t.	1,40E+07	2,10E+07	1,40E+07	3,33E+07	3,70E+07	3,52E+07	3,10E+07	2,79E+07	2,23E+07
A4D1M6	HCG19809	b.t.	b.t.	b.t.	b.t.	b.t.	b.t.	b.t.	b.t.	b.t.	2,60E+05	2,60E+05	3,90E+05	b.t.	b.t.	b.t.	9,50E+06	8,55E+06	6,84E+06
A4D2P2	Ras-related C3 botulinum toxin substrate 1	b.t.	b.t.	b.t.	b.t.	b.t.	b.t.	b.t.	b.t.	b.t.	5,60E+06	8,40E+06	5,60E+06	8,19E+06	9,10E+06	8,65E+06	9,10E+06	8,19E+06	6,55E+06
A4FU77	SNRNP200 protein	9,90E+04	1,49E+05	9,90E+04	3,48E+06	4,30E+06	1,90E+06	b.t.	b.t.	b.t.	b.t.	b.t.	b.t.	b.t.	b.t.	b.t.	1,30E+06	1,17E+06	9,36E+05
A6NJA2	Ubiquitin carboxyl-terminal hydrolase 14	b.t.	b.t.	b.t.	b.t.	b.t.	b.t.	b.t.	b.t.	b.t.	b.t.	b.t.	b.t.	5,40E+06	6,00E+06	5,70E+06	2,10E+06	1,89E+06	4,10E+06
A6NLN1	Polypyrimidine tract binding protein 1	2,40E+06	2,40E+06	3,60E+06	1,05E+07	1,40E+07	5,00E+06	3,95E+06	1,10E+06	3,40E+06	6,05E+06	5,60E+06	6,50E+06	3,96E+07	4,40E+07	4,18E+07	1,60E+07	3,10E+06	2,30E+06
A8K401	Prohibitin	b.t.	b.t.	b.t.	1,35E+06	4,50E+05	1,60E+06	1,07E+06	8,88E+05	7,10E+05	3,70E+06	5,55E+06	3,70E+06	1,44E+07	1,60E+07	1,52E+07	1,60E+07	1,44E+07	1,15E+07
A8K4Z4	60S acidic ribosomal protein P0	b.t.	b.t.	b.t.	1,19E+07	1,09E+07	9,90E+06	3,23E+06	6,60E+05	2,90E+06	3,20E+06	4,80E+06	3,20E+06	3,15E+07	3,50E+07	3,33E+07	1,00E+07	1,40E+06	5,40E+05
A8K644	Splicing factor, arginine/serine-rich 4	b.t.	b.t.	b.t.	b.t.	b.t.	b.t.	b.t.	b.t.	b.t.	b.t.	b.t.	b.t.	1,08E+07	1,20E+07	1,14E+07	5,40E+06	4,86E+06	3,89E+06
A8K8N7	Phosphoribosylformylglycinamidine synthase 1	b.t.	b.t.	b.t.	3,11E+06	3,00E+06	2,30E+06	b.t.	b.t.	b.t.	2,20E+06	3,30E+06	2,20E+06	7,92E+06	8,80E+06	8,36E+06	4,30E+06	3,87E+06	6,50E+06
A8MT02	Small nuclear ribonucleoprotein-associated proteins B	b.t.	b.t.	b.t.	6,60E+06	6,05E+06	5,50E+06	b.t.	b.t.	b.t.	7,90E+06	1,19E+07	7,90E+06	1,35E+07	1,50E+07	1,43E+07	2,50E+07	1,20E+06	1,20E+06
B0LPF3	Growth factor receptor-bound protein 2	b.t.	b.t.	b.t.	b.t.	b.t.	b.t.	b.t.	b.t.	b.t.	b.t.	b.t.	b.t.	9,90E+06	1,10E+07	1,05E+07	b.t.	b.t.	b.t.
B0ZBD0	40S ribosomal protein S19	b.t.	b.t.	b.t.	b.t.	b.t.	b.t.	b.t.	b.t.	b.t.	b.t.	b.t.	b.t.	1,17E+07	1,30E+07	1,24E+07	1,20E+07	1,08E+07	8,64E+06
B1AH77	Ras-related C3 botulinum toxin substrate 2	b.t.	b.t.	b.t.	b.t.	b.t.	b.t.	b.t.	b.t.	b.t.	5,60E+06	8,40E+06	5,60E+06	8,19E+06	9,10E+06	8,65E+06	9,10E+06	8,19E+06	6,55E+06
B2R4C0	60S ribosomal protein L18a	b.t.	b.t.	b.t.	b.t.	b.t.	b.t.	b.t.	b.t.	b.t.	5,90E+06	8,85E+06	5,90E+06	4,41E+06	4,90E+06	4,66E+06	8,30E+06	7,47E+06	5,98E+06
B2R4D8	60S ribosomal protein L27	b.t.	b.t.	b.t.	1,56E+06	1,43E+06	1,30E+06	b.t.	b.t.	b.t.	9,30E+06	1,40E+07	9,30E+06	1,80E+07	2,00E+07	1,90E+07	1,20E+07	1,08E+07	8,64E+06
B2R4R0	Histone H4	1,47E+06	2,00E+06	9,40E+05	4,60E+06	4,90E+07	3,50E+07	5,15E+07	1,70E+07	4,30E+07	8,60E+06	1,80E+06	8,80E+07	4,86E+08	5,40E+08	5,13E+08	6,70E+08	4,50E+07	6,80E+06
B2R5W3	Poly [ADP-ribose] polymerase 3	b.t.	b.t.	b.t.	3,36E+06	3,08E+06	2,80E+06	b.t.	b.t.	b.t.	b.t.	b.t.	b.t.	6,03E+06	6,70E+06	6,37E+06	4,10E+06	3,69E+06	5,10E+06
B2R6F3	Splicing factor arginine/serine-rich 3	b.t.	b.t.	b.t.	b.t.	b.t.	b.t.	b.t.	b.t.	b.t.	b.t.	b.t.	b.t.	1,62E+07	1,80E+07	1,71E+07	3,90E+07	3,51E+07	2,81E+07
B2RU06	EIF4G1 protein	b.t.	b.t.	b.t.	2,42E+06	2,60E+06	1,60E+06	b.t.	b.t.	b.t.	1,11E+06	3,10E+05	1,90E+06	7,74E+06	8,60E+06	8,17E+06	2,20E+06	1,98E+06	1,58E+06
B3GQS7	60 kDa heat shock protein, mitochondrial	b.t.	b.t.	b.t.	1,80E+06	1,65E+06	1,50E+06	1,65E+06	1,38E+06	1,10E+06	1,70E+07	1,30E+07	2,10E+07	8,64E+07	9,60E+07	9,12E+07	2,00E+08	7,30E+06	9,60E+06
B3KM80	Nucleolin 1	1,70E+06	1,70E+06	2,55E+06	1,13E+07	5,70E+06	1,20E+07	7,65E+06	6,38E+06	5,10E+06	1,90E+06	6,40E+06	1,90E+07	6,93E+07	7,70E+07	7,32E+07	4,10E+07	2,40E+07	1,30E+07
B3KS31	Tubulin	b.t.	b.t.	b.t.	b.t.	b.t.	b.t.	b.t.	b.t.	b.t.	b.t.	b.t.	b.t.	1,08E+07	1,20E+07	1,14E+07	2,30E+06	2,07E+06	1,66E+06
B4DG62	Hexokinase	b.t.	b.t.	b.t.	2,50E+06	2,50E+06	3,25E+06	b.t.	b.t.	b.t.	b.t.	b.t.	b.t.	b.t.	b.t.	b.t.	7,40E+05	4,80E+06	3,84E+06
B4DHQ3	Phosphoserine aminotransferase	b.t.	b.t.	b.t.	3,12E+06	2,86E+06	2,60E+06	b.t.	b.t.	b.t.	1,30E+06	1,30E+06	1,95E+06	7,20E+06	8,00E+06	7,60E+06	6,80E+06	1,70E+07	8,90E+06
B4DI38	Adenylyl cyclase-associated protein	b.t.	b.t.	b.t.	5,40E+06	5,40E+06	7,02E+06	b.t.	b.t.	b.t.	b.t.	b.t.	b.t.	9,90E+06	1,10E+07	1,05E+07	7,10E+06	6,39E+06	5,11E+06
B4DJV2	Citrate synthase, cytoplasmic	b.t.	b.t.	b.t.	7,22E+06	8,70E+06	4,10E+06	1,50E+06	1,25E+06	1,00E+06	2,30E+06	2,90E+06	4,05E+06	1,08E+07	1,20E+07	1,14E+07	1,70E+07	1,20E+07	2,40E+07
B4DMH3	Coronin-1A	b.t.	b.t.	b.t.	b.t.	b.t.	b.t.	b.t.	b.t.	b.t.	5,30E+05	5,30E+05	7,95E+05	b.t.	b.t.	b.t.	7,97E+06	5,30E+06	7,60E+06
B4DUC8	S-methyl-5'-thioadenosine phosphorylase	b.t.	b.t.	b.t.	8,50E+05	8,50E+05	1,11E+06	b.t.	b.t.	b.t.	b.t.	b.t.	b.t.	b.t.	b.t.	b.t.	b.t.	b.t.	b.t.
B4DV00	Protein arginine N-methyltransferase 5	b.t.	b.t.	b.t.	2,00E+06	2,00E+06	2,60E+06	b.t.	b.t.	b.t.	b.t.	b.t.	b.t.	b.t.	b.t.	b.t.	8,22E+06	5,80E+06	7,60E+06
B4DWC4	Chloride intracellular channel protein	b.t.	b.t.	b.t.	b.t.	b.t.	b.t.	b.t.	b.t.	b.t.	b.t.	b.t.	b.t.	1,17E+06	1,30E+06	1,24E+06	b.t.	b.t.	b.t.
B4DXW1	Actin-related protein 3	1,00E+06	1,00E+06	1,50E+06	3,24E+06	2,97E+06	2,70E+06	b.t.	b.t.	b.t.	3,85E+06	1,10E+06	6,60E+06	1,44E+07	1,60E+07	1,52E+07	6,10E+06	5,49E+06	2,90E+06
B4DY09	Interleukin enhancer-binding factor 2	5,50E+05	8,25E+05	5,50E+05	7,45E+06	3,00E+06	8,50E+06	5,40E+06	4,50E+06	3,60E+06	3,30E+06	1,90E+06	2,80E+06	1,62E+07	1,80E+07	1,71E+07	1,30E+07	2,70E+07	1,30E+07
B4DZ08	Aconitate hydratase, mitochondrial	b.t.	b.t.	b.t.	b.t.	b.t.	b.t.	b.t.	b.t.	b.t.	b.t.	b.t.	b.t.	5,04E+06	5,60E+06	5,32E+06	2,10E+06	1,89E+06	1,51E+06
B4E0E1	Poly [ADP-ribose] polymerase 2	b.t.	b.t.	b.t.	3,36E+06	3,08E+06	2,80E+06	b.t.	b.t.	b.t.	b.t.	b.t.	b.t.	6,03E+06	6,70E+06	6,37E+06	4,10E+06	3,69E+06	5,10E+06
B5BU25	U2 small nuclear RNA auxiliary factor 2	5,20E+06	5,20E+06	7,80E+06	2,16E+06	1,98E+06	1,80E+06	b.t.	b.t.	b.t.	b.t.	b.t.	b.t.	6,75E+06	7,50E+06	7,13E+06	1,90E+07	1,71E+07	2,20E+06
B5BUB5	Autoantigen La	b.t.	b.t.	b.t.	6,05E+06	3,70E+06	6,00E+06	2,85E+06	2,38E+06	1,90E+06	7,40E+05	7,40E+05	1,11E+06	7,02E+06	7,80E+06	7,41E+06	9,30E+06	8,37E+06	1,10E+06
B5BUE6	ATP-dependent RNA helicase DDX5	b.t.	b.t.	b.t.	b.t.	b.t.	b.t.	b.t.	b.t.	b.t.	b.t.	b.t.	b.t.	6,03E+06	6,70E+06	6,37E+06	3,70E+06	3,33E+06	2,66E+06
B5MCX3	Septin-2	b.t.	b.t.	b.t.	3,72E+06	3,41E+06	3,10E+06	b.t.	b.t.	b.t.	5,30E+06	7,95E+06	5,30E+06	7,02E+06	7,80E+06	7,41E+06	b.t.	b.t.	b.t.
B5MDF5	GTP-binding nuclear protein Ran	2,80E+06	4,20E+06	2,80E+06	1,03E+07	7,90E+06	9,00E+06	3,30E+06	2,75E+06	2,20E+06	2,80E+07	4,20E+07	2,80E+07	4,77E+07	5,30E+07	5,04E+07	2,90E+07	2,61E+07	1,80E+06
B7Z4E3	60S ribosomal protein L31	b.t.	b.t.	b.t.	b.t.	b.t.	b.t.	b.t.	b.t.	b.t.	7,30E+06	1,10E+07	7,30E+06	9,90E+06	1,10E+07	1,05E+07	5,00E+06	9,60E+05	7,68E+05
B8ZWD1	Acyl-CoA-binding protein	b.t.	b.t.	b.t.	b.t.	b.t.	b.t.	b.t.	b.t.	b.t.	b.t.	b.t.	b.t.	b.t.	b.t.	b.t.	1,20E+07	1,08E+07	8,64E+06
B9EKV4	Aldehyde dehydrogenase 9 family, member A1	b.t.	b.t.	b.t.	b.t.	b.t.	b.t.	1,17E+06	9,75E+05	7,80E+05	4,40E+05	4,40E+05	6,60E+05	b.t.	b.t.	b.t.	3,20E+06	8,30E+06	6,64E+06
B9VP24	60 kDa chaperonin	b.t.	b.t.	b.t.	b.t.	b.t.	b.t.	b.t.	b.t.	b.t.	6,60E+05	6,60E+05	9,90E+05	b.t.	b.t.	b.t.	b.t.	b.t.	b.t.
C9J8H1	V-type proton ATPase subunit E 1	b.t.	b.t.	b.t.	b.t.	b.t.	b.t.	b.t.	b.t.	b.t.	b.t.	b.t.	b.t.	6,57E+06	7,30E+06	6,94E+06	b.t.	b.t.	b.t.
C9JXB8	60S ribosomal protein L24	b.t.	b.t.	b.t.	b.t.	b.t.	b.t.	b.t.	b.t.	b.t.	b.t.	b.t.	b.t.	8,10E+06	9,00E+06	8,55E+06	b.t.	b.t.	b.t.
D3DP78	Aspartyl-tRNA synthetase	b.t.	b.t.	b.t.	b.t.	b.t.	b.t.	b.t.	b.t.	b.t.	b.t.	b.t.	b.t.	8,91E+06	9,90E+06	9,41E+06	3,90E+06	3,51E+06	1,10E+07
D3DUG9	Ubiquitin specific peptidase 14	b.t.	b.t.	b.t.	b.t.	b.t.	b.t.	b.t.	b.t.	b.t.	b.t.	b.t.	b.t.	5,40E+06	6,00E+06	5,70E+06	2,10E+06	1,89E+06	4,10E+06
D3DWK1	Eukaryotic translation elongation factor 1	b.t.	b.t.	b.t.	b.t.	b.t.	b.t.	b.t.	b.t.	b.t.	2,09E+06	2,80E+05	3,90E+06	b.t.	b.t.	b.t.	6,10E+06	5,49E+06	1,30E+06
D6RAN4	60S ribosomal protein L9	b.t.	b.t.	b.t.	b.t.	b.t.	b.t.	b.t.	b.t.	b.t.	7,50E+06	1,13E+07	7,50E+06	1,62E+07	1,80E+07	1,71E+07	1,50E+07	1,35E+07	1,20E+06
D6REX3	Protein transport protein Sec31A	b.t.	b.t.	b.t.	b.t.	b.t.	b.t.	b.t.	b.t.	b.t.	b.t.	b.t.	b.t.	b.t.	b.t.	b.t.	8,40E+06	7,56E+06	6,05E+06
E1NZA1	Peroxisome proliferator activated receptor interacting complex protei	b.t.	b.t.	b.t.	b.t.	b.t.	b.t.	b.t.	b.t.	b.t.	b.t.	b.t.	b.t.	b.t.	b.t.	b.t.	2,90E+06	2,61E+06	2,09E+06
E1P5S2	RNA-binding region (RNP1. RRM) containing 2	b.t.	b.t.	b.t.	b.t.	b.t.	b.t.	b.t.	b.t.	b.t.	b.t.	b.t.	b.t.	b.t.	b.t.	b.t.	6,50E+06	5,85E+06	4,68E+06
E4W6B6	RPL27/NME2 fusion protein	b.t.	b.t.	b.t.	1,56E+06	1,43E+06	1,30E+06	b.t.	b.t.	b.t.	9,30E+06	1,40E+07	9,30E+06	1,80E+07	2,00E+07	1,90E+07	1,20E+07	1,08E+07	8,64E+06
E5RK69	Annexin A2	b.t.	b.t.	b.t.	3,80E+06	3,80E+06	4,94E+06	b.t.	b.t.	b.t.	b.t.	b.t.	b.t.	b.t.	b.t.	b.t.	1,30E+06	1,17E+06	3,50E+06
E7ENZ3	T-complex protein 1	b.t.	b.t.	b.t.	4,68E+06	4,29E+06	3,90E+06	2,40E+06	2,00E+06	1,60E+06	b.t.	b.t.	b.t.	1,98E+07	2,20E+07	2,09E+07	2,20E+07	1,98E+07	3,50E+06
E7ER27	Peroxisomal multifunctional enzyme type 2	b.t.	b.t.	b.t.	4,20E+06	4,20E+06	5,46E+06	b.t.	b.t.	b.t.	b.t.	b.t.	b.t.	8,46E+06	9,40E+06	8,93E+06	1,60E+06	1,44E+06	1,15E+06
E7EUU4	Eukaryotic translation initiation factor 4 gamma 1	b.t.	b.t.	b.t.	2,42E+06	2,60E+06	1,60E+06	b.t.	b.t.	b.t.	1,11E+06	3,10E+05	1,90E+06	7,74E+06	8,60E+06	8,17E+06	2,20E+06	1,98E+06	1,58E+06

E9PCR7	2-oxoglutarate dehydrogenase, mitochondrial	b.t.	b.t.	b.t.	b.t.	b.t.	b.t.	b.t.	b.t.	b.t.	b.t.	b.t.	b.t.	3,33E+06	3,70E+06	3,52E+06	1,10E+06	4,90E+06	3,92E+06
E9PEB5	Far upstream element-binding protein 1	b.t.	b.t.	b.t.	b.t.	b.t.	b.t.	b.t.	b.t.	b.t.	b.t.	b.t.	b.t.	b.t.	b.t.	b.t.	1,70E+07	1,53E+07	1,22E+07
E9PEX6	Dihydrolipoyl dehydrogenase, cytoplasmic	b.t.	b.t.	b.t.	b.t.	b.t.	b.t.	b.t.	b.t.	b.t.	b.t.	b.t.	b.t.	b.t.	b.t.	b.t.	4,40E+06	3,96E+06	4,30E+06
E9PGT1	Translin	b.t.	b.t.	b.t.	1,68E+06	1,54E+06	1,40E+06	b.t.	b.t.	b.t.	b.t.	b.t.	b.t.	7,74E+06	8,60E+06	8,17E+06	b.t.	b.t.	b.t.
E9PKD5	26S protease regulatory subunit 6A	b.t.	b.t.	b.t.	b.t.	b.t.	b.t.	b.t.	b.t.	b.t.	1,10E+06	1,10E+06	1,65E+06	6,21E+06	6,90E+06	6,56E+06	3,00E+06	8,10E+06	2,80E+06
E9PNQ6	Thioredoxin reductase 1	1,70E+06	1,70E+06	2,55E+06	3,20E+07	5,30E+07	7,80E+06	1,65E+06	1,38E+06	1,10E+06	2,60E+06	3,70E+06	8,00E+06	7,29E+07	8,10E+07	7,70E+07	2,60E+07	3,00E+07	4,40E+07
E9PP21	Cysteine and glycine-rich protein 1	b.t.	b.t.	b.t.	b.t.	b.t.	b.t.	b.t.	b.t.	b.t.	b.t.	b.t.	b.t.	5,40E+06	6,00E+06	5,70E+06	b.t.	b.t.	b.t.
E9PP73	Coatomer subunit beta	b.t.	b.t.	b.t.	3,22E+06	4,90E+06	1,10E+06	b.t.	b.t.	b.t.	b.t.	b.t.	b.t.	7,02E+06	7,80E+06	7,41E+06	5,99E+06	2,60E+06	6,70E+06
F4ZW62	NF45	5,50E+05	8,25E+05	5,50E+05	7,45E+06	3,00E+06	8,50E+06	5,40E+06	4,50E+06	3,60E+06	3,30E+06	1,90E+06	2,80E+06	1,62E+07	1,80E+07	1,71E+07	1,30E+07	2,70E+07	1,30E+07
F4ZW64	NF90a	b.t.	b.t.	b.t.	1,32E+06	1,21E+06	1,10E+06	b.t.	b.t.	b.t.	6,80E+05	6,80E+05	1,02E+06	1,08E+07	1,20E+07	1,14E+07	6,40E+06	7,40E+06	6,30E+06
F4ZW65	NF90b	b.t.	b.t.	b.t.	1,32E+06	1,21E+06	1,10E+06	b.t.	b.t.	b.t.	6,80E+05	6,80E+05	1,02E+06	1,08E+07	1,20E+07	1,14E+07	6,40E+06	7,40E+06	6,30E+06
F4ZW66	NF110b	b.t.	b.t.	b.t.	1,32E+06	1,21E+06	1,10E+06	b.t.	b.t.	b.t.	6,80E+05	6,80E+05	1,02E+06	1,08E+07	1,20E+07	1,14E+07	6,40E+06	7,40E+06	6,30E+06
F5H2F4	C-1-tetrahydrofolate synthase	b.t.	b.t.	b.t.	2,52E+06	2,31E+06	2,10E+06	b.t.	b.t.	b.t.	1,36E+06	7,10E+05	2,00E+06	1,17E+07	1,30E+07	1,24E+07	1,10E+07	1,10E+07	1,00E+07
F5H365	Protein transport protein Sec23A	b.t.	b.t.	b.t.	8,28E+05	6,90E+05	6,90E+05	b.t.	b.t.	b.t.	b.t.	b.t.	b.t.	9,90E+06	1,10E+07	1,05E+07	4,00E+06	3,60E+06	9,40E+06
F6KPG5	Albumin	3,50E+05	5,25E+05	3,50E+05	1,20E+07	4,30E+07	3,10E+07	5,00E+04	1,28E+06	2,50E+06	1,10E+07	7,00E+06	2,40E+07	1,71E+08	1,90E+08	1,81E+08	8,60E+07	8,10E+07	1,60E+08
F6U211	40S ribosomal protein S10	b.t.	b.t.	b.t.	9,48E+06	8,69E+06	7,90E+06	8,25E+06	6,88E+06	5,50E+06	1,20E+06	1,80E+06	2,40E+06	4,50E+06	5,00E+06	4,75E+06	7,90E+07	7,11E+07	5,69E+07
F8VRV5	Dynein light chain 1	b.t.	b.t.	b.t.	b.t.	b.t.	b.t.	b.t.	b.t.	b.t.	b.t.	b.t.	b.t.	b.t.	b.t.	b.t.	1,40E+07	1,26E+07	1,01E+07
F8VZX2	Poly(rC)-binding protein 2c	b.t.	b.t.	b.t.	b.t.	b.t.	b.t.	2,10E+06	1,75E+06	1,40E+06	3,90E+05	3,90E+05	5,85E+05	6,48E+06	7,20E+06	6,84E+06	1,10E+06	1,10E+06	8,80E+05
F8W1A4	Adenylate kinase 2	b.t.	b.t.	b.t.	2,64E+06	2,42E+06	2,20E+06	b.t.	b.t.	b.t.	b.t.	b.t.	b.t.	4,50E+06	5,00E+06	4,75E+06	3,72E+05	3,35E+05	3,10E+05
F8W6I7	Heterogeneous nuclear ribonucleoprotein A1	2,10E+06	2,10E+06	3,15E+06	7,80E+06	7,80E+06	1,01E+07	4,20E+06	3,50E+06	2,80E+06	1,40E+07	2,10E+07	1,40E+07	5,40E+07	6,00E+07	5,70E+07	2,60E+07	1,20E+06	1,10E+06
F8W727	60S ribosomal protein L32	b.t.	b.t.	b.t.	b.t.	b.t.	b.t.	b.t.	b.t.	b.t.	3,00E+06	4,50E+06	3,00E+06	5,67E+06	6,30E+06	5,99E+06	2,30E+06	2,07E+06	1,66E+06
G3V1A4	Cofilin 1	5,20E+06	6,60E+06	3,80E+06	2,00E+07	1,90E+07	1,50E+07	7,95E+06	6,63E+06	5,30E+06	1,70E+06	1,80E+06	5,40E+06	8,82E+07	9,80E+07	9,31E+07	8,80E+07	7,92E+07	2,00E+06
G3V1B3	60S ribosomal protein L21	b.t.	b.t.	b.t.	b.t.	b.t.	b.t.	b.t.	b.t.	b.t.	3,70E+06	5,55E+06	3,70E+06	b.t.	b.t.	b.t.	b.t.	b.t.	b.t.
G3V1C3	Apoptosis inhibitor 5	b.t.	b.t.	b.t.	1,58E+06	1,80E+06	9,70E+05	b.t.	b.t.	b.t.	2,60E+06	3,90E+06	2,60E+06	4,77E+06	5,30E+06	5,04E+06	3,00E+06	2,70E+06	3,00E+06
G3V4W0	Heterogeneous nuclear ribonucleoproteins C1/C2	b.t.	b.t.	b.t.	2,28E+06	2,09E+06	1,90E+06	5,00E+06	3,40E+06	3,30E+06	1,30E+06	2,60E+06	2,60E+06	5,85E+07	6,50E+07	6,18E+07	6,10E+07	5,49E+07	4,39E+07
G8JLB6	Heterogeneous nuclear ribonucleoprotein H	b.t.	b.t.	b.t.	9,24E+06	8,47E+06	7,70E+06	6,45E+06	5,38E+06	4,30E+06	4,28E+06	3,50E+05	8,20E+06	1,17E+07	1,30E+07	1,24E+07	2,50E+07	2,25E+07	1,80E+07
H0Y8C6	Importin-5	b.t.	b.t.	b.t.	6,54E+06	9,30E+06	2,70E+06	b.t.	b.t.	b.t.	b.t.	b.t.	b.t.	1,26E+07	1,40E+07	1,33E+07	3,87E+06	2,00E+06	4,10E+06
H0YA96	Heterogeneous nuclear ribonucleoprotein D0	b.t.	b.t.	b.t.	6,96E+06	6,38E+06	5,80E+06	5,10E+06	4,25E+06	3,40E+06	3,45E+06	1,90E+06	5,00E+06	2,79E+07	3,10E+07	2,95E+07	1,70E+07	1,53E+07	1,80E+06
H0YAS8	Clusterin	b.t.	b.t.	b.t.	2,70E+06	2,60E+06	2,00E+06	b.t.	b.t.	b.t.	1,40E+06	3,10E+06	4,70E+06	1,98E+07	2,20E+07	2,09E+07	5,90E+06	1,10E+07	5,70E+06
H0YJC0	26S protease regulatory subunit 10B	b.t.	b.t.	b.t.	1,68E+06	1,54E+06	1,40E+06	b.t.	b.t.	b.t.	b.t.	b.t.	b.t.	4,50E+06	5,00E+06	4,75E+06	1,30E+06	1,17E+06	9,36E+05
H0YJG7	Activator of 90 kDa heat shock protein ATPase homolog 1	b.t.	b.t.	b.t.	3,96E+06	3,63E+06	3,30E+06	4,50E+06	3,75E+06	3,00E+06	b.t.	b.t.	b.t.	4,41E+07	4,90E+07	4,66E+07	4,70E+06	4,23E+06	3,38E+06
H0YKK0	Small nuclear ribonucleoprotein polypeptide A	b.t.	b.t.	b.t.	4,56E+06	4,18E+06	3,80E+06	b.t.	b.t.	b.t.	b.t.	b.t.	b.t.	2,52E+06	2,80E+06	2,66E+06	b.t.	b.t.	b.t.
H0YN26	Acidic leucine-rich nuclear phosphoprotein 32 family member A	b.t.	b.t.	b.t.	6,72E+06	6,16E+06	5,60E+06	2,85E+06	2,38E+06	1,90E+06	4,10E+06	6,15E+06	4,10E+06	4,50E+06	5,00E+06	4,75E+06	2,50E+07	2,25E+07	1,80E+07
H3BM89	60S ribosomal protein L4	b.t.	b.t.	b.t.	b.t.	b.t.	b.t.	b.t.	b.t.	b.t.	b.t.	b.t.	b.t.	4,59E+07	5,10E+07	4,85E+07	4,90E+07	4,41E+07	3,53E+07
H3BNI9	Casein kinase II	b.t.	b.t.	b.t.	9,48E+05	8,69E+05	7,90E+05	b.t.	b.t.	b.t.	2,30E+06	3,45E+06	2,30E+06	4,41E+06	4,90E+06	4,66E+06	b.t.	b.t.	b.t.
H3BRU6	Poly(rC)-binding protein 2a	b.t.	b.t.	b.t.	b.t.	b.t.	b.t.	2,10E+06	1,75E+06	1,40E+06	3,90E+05	3,90E+05	5,85E+05	6,48E+06	7,20E+06	6,84E+06	1,10E+06	1,10E+06	8,80E+05
H3BSC1	Ras-related protein Rab-11A	b.t.	b.t.	b.t.	9,72E+06	8,91E+06	8,10E+06	b.t.	b.t.	b.t.	b.t.	b.t.	b.t.	b.t.	b.t.	b.t.	2,40E+05	2,40E+05	1,92E+05
H6VRG2	Keratin 1	6,25E+06	1,00E+07	2,50E+06	4,40E+06	8,20E+07	2,60E+07	2,70E+06	1,20E+06	2,10E+06	5,90E+06	6,00E+06	1,10E+07	1,44E+08	1,60E+08	1,52E+08	2,30E+07	2,80E+07	5,50E+07
H9ZYJ2	Thioredoxin	b.t.	b.t.	b.t.	1,56E+07	1,43E+07	1,30E+07	1,10E+06	1,10E+06	1,32E+06	1,70E+06	7,50E+05	1,60E+06	4,77E+07	5,30E+07	5,04E+07	6,50E+07	5,85E+07	4,68E+07
I3L0A0	HCG2044781	b.t.	b.t.	b.t.	b.t.	b.t.	b.t.	b.t.	b.t.	b.t.	1,10E+07	1,65E+07	1,10E+07	1,80E+07	2,00E+07	1,90E+07	5,80E+06	5,22E+06	4,18E+06
I3L397	Eukaryotic translation initiation factor 5A	1,70E+06	1,70E+06	2,55E+06	4,24E+06	1,20E+06	5,20E+06	5,55E+06	4,63E+06	3,70E+06	2,16E+06	4,20E+05	3,90E+06	3,33E+07	3,70E+07	3,52E+07	2,70E+07	2,43E+07	2,30E+06
I6L965	KRT18 protein	b.t.	b.t.	b.t.	3,14E+06	3,20E+06	2,20E+06	b.t.	b.t.	b.t.	1,90E+06	2,70E+06	1,50E+06	8,01E+06	8,90E+06	8,46E+06	1,00E+07	1,70E+07	1,30E+07
J3K000	PEPD protein	b.t.	b.t.	b.t.	3,50E+06	3,50E+06	4,55E+06	b.t.	b.t.	b.t.	b.t.	b.t.	b.t.	b.t.	b.t.	b.t.	2,10E+06	2,70E+06	4,40E+06
J3KN67	Tropomyosin alpha-3	b.t.	b.t.	b.t.	b.t.	b.t.	b.t.	b.t.	b.t.	b.t.	5,90E+05	5,90E+05	8,85E+05	9,00E+06	1,00E+07	9,50E+06	6,80E+06	6,12E+06	4,90E+06
J3KNE3	Platelet-activating factor acetylhydrolase IB subunit beta	b.t.	b.t.	b.t.	6,36E+06	5,83E+06	5,30E+06	b.t.	b.t.	b.t.	b.t.	b.t.	b.t.	5,67E+06	6,30E+06	5,99E+06	2,20E+07	1,98E+07	1,58E+07
J3KQ18	D-dopachrome decarboxylase	b.t.	b.t.	b.t.	7,20E+06	6,60E+06	6,00E+06	3,00E+06	2,50E+06	2,00E+06	1,70E+06	2,55E+06	1,70E+06	b.t.	b.t.	b.t.	3,50E+06	3,15E+06	2,52E+06
J3KQ32	Obg-like ATPase 1a	6,90E+05	1,04E+06	6,90E+05	7,63E+06	7,70E+06	5,40E+06	b.t.	b.t.	b.t.	1,70E+06	1,70E+06	2,55E+06	1,80E+07	2,00E+07	1,90E+07	5,80E+06	5,80E+06	7,60E+06
J3QQV1	60S ribosomal protein L26	b.t.	b.t.	b.t.	b.t.	b.t.	b.t.	b.t.	b.t.	b.t.	3,80E+06	5,70E+06	3,80E+06	b.t.	b.t.	b.t.	7,60E+06	6,84E+06	5,47E+06
J3QQX2	Rho GDP-dissociation inhibitor 1	b.t.	b.t.	b.t.	2,16E+06	1,98E+06	1,80E+06	3,75E+06	3,13E+06	2,50E+06	7,00E+06	1,05E+07	7,00E+06	9,90E+06	1,10E+07	1,05E+07	1,30E+07	1,17E+07	1,70E+06
J3QR09	Ribosomal protein L19	b.t.	b.t.	b.t.	b.t.	b.t.	b.t.	b.t.	b.t.	b.t.	b.t.	b.t.	b.t.	7,11E+06	7,90E+06	7,51E+06	4,90E+06	4,41E+06	3,53E+06
J3QS39	Polyubiquitin-B	1,23E+06	7,60E+05	1,70E+06	2,80E+06	6,60E+06	2,80E+07	1,10E+07	9,13E+06	7,30E+06	3,00E+06	2,60E+06	5,90E+07	1,08E+08	1,20E+08	1,14E+08	1,30E+08	4,20E+07	1,80E+07
K7ELC2	40S ribosomal protein S15	b.t.	b.t.	b.t.	b.t.	b.t.	b.t.	b.t.	b.t.	b.t.	1,20E+06	1,80E+06	1,20E+06	6,12E+06	6,80E+06	6,46E+06	9,10E+06	8,19E+06	4,00E+05
K7ENG2	Splicing factor U2AF 65 kDa subunit	5,20E+06	5,20E+06	7,80E+06	2,16E+06	1,98E+06	1,80E+06	b.t.	b.t.	b.t.	b.t.	b.t.	b.t.	6,75E+06	7,50E+06	7,13E+06	1,90E+07	1,71E+07	2,20E+06
K7ER00	Phenylalanine-tRNA ligase alpha subunit	b.t.	b.t.	b.t.	b.t.	b.t.	b.t.	2,10E+06	1,75E+06	1,40E+06	b.t.	b.t.	b.t.	5,49E+06	6,10E+06	5,80E+06	3,90E+06	3,51E+06	2,81E+06
K7ERE3	Keratin, type I cytoskeletal 13	b.t.	b.t.	b.t.	6,24E+06	5,72E+06	5,20E+06	b.t.	b.t.	b.t.	b.t.	b.t.	b.t.	9,90E+06	1,10E+07	1,05E+07	3,40E+06	3,06E+06	1,90E+07
L8B4J3	Ubiquitin C	1,23E+06	7,60E+05	1,70E+06	2,80E+06	6,60E+06	2,80E+07	1,10E+07	9,13E+06	7,30E+06	3,00E+06	2,60E+06	5,90E+07	1,08E+08	1,20E+08	1,14E+08	1,30E+08	4,20E+07	1,80E+07
M0QYS1	60S ribosomal protein L13a	b.t.	b.t.	b.t.	b.t.	b.t.	b.t.	b.t.	b.t.	b.t.	1,80E+06	2,70E+06	1,80E+06	3,06E+06	3,40E+06	3,23E+06	4,00E+06	3,60E+06	2,88E+06
M0QZC5	40S ribosomal protein S11	b.t.	b.t.	b.t.	b.t.	b.t.	b.t.	b.t.	b.t.	b.t.	8,30E+06	1,25E+07	8,30E+06	5,49E+06	6,10E+06	5,80E+06	1,30E+07	1,17E+07	9,36E+06
M0R0F0	40S ribosomal protein S5	b.t.	b.t.	b.t.	b.t.	b.t.	b.t.	b.t.	b.t.	b.t.	b.t.	b.t.	b.t.	3,06E+06	3,40E+06	3,23E+06	1,60E+07	1,44E+07	1,15E+07
M0R0R2	40S ribosomal protein S5	b.t.	b.t.	b.t.	b.t.	b.t.	b.t.	b.t.	b.t.	b.t.	b.t.	b.t.	b.t.	3,06E+06	3,40E+06	3,23E+06	1,60E+07	1,44E+07	1,15E+07
M0R210	40S ribosomal protein S16	b.t.	b.t.	b.t.	2,76E+06	2,53E+06	2,30E+06	b.t.	b.t.	b.t.	1,30E+07	1,95E+07	1,30E+07	9,90E+06	1,10E+07	1,05E+07	2,20E+07	1,98E+07	1,20E+06
M1V481	Tyrosine-protein kinase receptor	b.t.	b.t.	b.t.	2,20E+06	2,20E+06	2,86E+06	b.t.	b.t.	b.t.	b.t.	b.t.	b.t.	b.t.	b.t.	b.t.	2,10E+06	1,89E+06	1,51E+06
M1VKJ2	Kinesin-like protein	b.t.	b.t.	b.t.	2,20E+06	2,20E+06	2,86E+06	b.t.	b.t.	b.t.	b.t.	b.t.	b.t.	b.t.	b.t.	b.t.	2,10E+06	1,89E+06	1,51E+06
O00231	26S proteasome non-ATPase regulatory subunit 11	b.t.	b.t.	b.t.	3,70E+06	3,90E+06	2,50E+06	b.t.	b.t.	b.t.	8,00E+05	8,00E+05	1,20E+06	6,12E+06	6,80E+06	6,46E+06	4,60E+06	9,60E+06	8,90E+06

O00303	Eukaryotic translation initiation factor 3 subunit F	b.t.	b.t.	b.t.	b.t.	b.t.	b.t.	b.t.	b.t.	b.t.	b.t.	b.t.	b.t.	1,08E+07	1,20E+07	1,14E+07	1,00E+07	8,70E+06	8,10E+06
O14920	Inhibitor of nuclear factor κ-B kinase subunit B	b.t.	b.t.	b.t.	2,31E+06	2,10E+06	1,80E+06	2,21E+06	2,15E+06	2,18E+06	b.t.	b.t.	b.t.	2,11E+06	1,80E+06	1,00E+06	2,31E+06	2,45E+06	2,18E+06
O15067	Phosphoribosylformylglycinamidine synthase 2	b.t.	b.t.	b.t.	3,11E+06	3,00E+06	2,30E+06	b.t.	b.t.	b.t.	2,20E+06	3,30E+06	2,20E+06	7,92E+06	8,80E+06	8,36E+06	4,30E+06	3,87E+06	6,50E+06
O15144	Actin-related protein 2/3 complex subunit 2	b.t.	b.t.	b.t.	2,76E+06	2,53E+06	2,30E+06	b.t.	b.t.	b.t.	b.t.	b.t.	b.t.	b.t.	b.t.	b.t.	8,60E+06	7,74E+06	9,50E+05
O43143	Pre-mRNA-splicing factor ATP-dependent RNA helicase DHX15	1,30E+06	1,30E+06	1,95E+06	4,62E+06	6,30E+06	2,10E+06	b.t.	b.t.	b.t.	b.t.	b.t.	b.t.	6,84E+06	7,60E+06	7,22E+06	5,60E+06	1,00E+07	9,30E+06
O43175	D-3-phosphoglycerate dehydrogenase	b.t.	b.t.	b.t.	3,72E+06	3,41E+06	3,10E+06	b.t.	b.t.	b.t.	2,70E+06	2,00E+06	3,40E+06	1,17E+07	1,30E+07	1,24E+07	1,30E+07	1,70E+06	1,00E+07
O43242	26S proteasome non-ATPase regulatory subunit 3	b.t.	b.t.	b.t.	7,44E+05	6,82E+05	6,20E+05	b.t.	b.t.	b.t.	b.t.	b.t.	b.t.	b.t.	b.t.	b.t.	2,30E+06	5,10E+06	5,20E+06
O43390	Heterogeneous nuclear ribonucleoprotein R	b.t.	b.t.	b.t.	4,92E+06	4,51E+06	4,10E+06	3,15E+06	2,63E+06	2,10E+06	2,00E+06	1,60E+06	2,40E+06	7,83E+06	8,70E+06	8,27E+06	4,60E+06	4,14E+06	6,20E+06
O43592	Exportin-T	b.t.	b.t.	b.t.	4,80E+06	4,80E+06	6,24E+06	b.t.	b.t.	b.t.	b.t.	b.t.	b.t.	b.t.	b.t.	b.t.	6,60E+06	5,94E+06	5,50E+06
O43707	Alpha-actinin-4	b.t.	b.t.	b.t.	7,09E+06	6,20E+06	5,70E+06	b.t.	b.t.	b.t.	1,60E+06	1,60E+06	1,60E+06	2,07E+07	2,30E+07	2,19E+07	1,10E+07	1,30E+07	1,40E+07
O43776	Asparagine-tRNA ligase	1,10E+06	1,10E+06	1,65E+06	2,70E+06	3,30E+06	1,50E+06	b.t.	b.t.	b.t.	b.t.	b.t.	b.t.	4,23E+06	4,70E+06	4,47E+06	2,70E+06	5,50E+06	1,70E+07
O60218	Aldo-keto reductase family 1 member B10	3,50E+06	2,50E+06	4,50E+06	2,04E+07	1,00E+07	2,20E+07	1,37E+07	5,40E+06	1,10E+07	1,50E+06	3,90E+07	7,50E+07	1,26E+08	1,40E+08	1,33E+08	1,10E+08	1,10E+07	1,00E+07
O60506	Heterogeneous nuclear ribonucleoprotein Q	b.t.	b.t.	b.t.	7,47E+06	5,00E+06	7,10E+06	6,60E+06	5,50E+06	4,40E+06	2,00E+06	2,00E+06	3,00E+06	2,16E+07	2,40E+07	2,28E+07	9,80E+06	3,50E+06	1,40E+07
O60701	UDP-glucose 6-dehydrogenase	2,70E+06	2,70E+06	4,05E+06	4,14E+07	5,90E+07	1,70E+07	1,70E+06	5,90E+05	1,40E+06	5,10E+06	5,90E+06	1,40E+07	1,26E+08	1,40E+08	1,33E+08	5,30E+07	2,20E+07	7,70E+07
O60814	Histone H2B type 1-K	b.t.	b.t.	b.t.	b.t.	b.t.	b.t.	b.t.	b.t.	b.t.	2,80E+06	4,20E+06	2,80E+06	1,08E+07	1,20E+07	1,14E+07	b.t.	b.t.	b.t.
O75083	WD repeat-containing protein 1	b.t.	b.t.	b.t.	b.t.	b.t.	b.t.	b.t.	b.t.	b.t.	b.t.	b.t.	b.t.	4,32E+06	4,80E+06	4,56E+06	3,30E+06	3,70E+06	4,60E+06
O75367	Core histone macro-H2A.1	b.t.	b.t.	b.t.	3,84E+06	3,52E+06	3,20E+06	1,34E+06	1,11E+06	8,90E+05	5,20E+06	7,80E+06	5,20E+06	6,84E+06	7,60E+06	7,22E+06	4,50E+06	4,05E+06	3,24E+06
O75369	Filamin-B	8,30E+05	8,30E+05	1,25E+06	1,00E+06	7,60E+06	4,50E+06	b.t.	b.t.	b.t.	3,20E+06	3,60E+06	9,40E+06	3,87E+07	4,30E+07	4,09E+07	1,30E+07	1,50E+07	3,10E+07
O75390	Citrate synthase, mitochondrial	b.t.	b.t.	b.t.	7,22E+06	8,70E+06	4,10E+06	1,50E+06	1,25E+06	1,00E+06	2,30E+06	2,90E+06	4,05E+06	1,08E+07	1,20E+07	1,14E+07	1,70E+07	1,20E+07	2,40E+07
O75533	Splicing factor 3B subunit 1	b.t.	b.t.	b.t.	2,24E+06	2,80E+06	1,20E+06	b.t.	b.t.	b.t.	b.t.	b.t.	b.t.	b.t.	b.t.	b.t.	1,30E+06	1,17E+06	9,36E+05
O75643	U5 small nuclear ribonucleoprotein 200 kDa helicase	9,90E+04	1,49E+05	9,90E+04	3,48E+06	4,30E+06	1,90E+06	b.t.	b.t.	b.t.	b.t.	b.t.	b.t.	b.t.	b.t.	b.t.	1,30E+06	1,17E+06	9,36E+05
O76003	Glutaredoxin-3	b.t.	b.t.	b.t.	b.t.	b.t.	b.t.	b.t.	b.t.	b.t.	b.t.	b.t.	b.t.	6,75E+06	7,50E+06	7,13E+06	8,90E+06	8,90E+06	7,12E+06
O76021	Ribosomal L1 domain-containing protein 1	b.t.	b.t.	b.t.	2,26E+06	1,30E+06	2,30E+06	2,40E+06	2,00E+06	1,60E+06	b.t.	b.t.	b.t.	7,56E+06	8,40E+06	7,98E+06	4,10E+06	3,69E+06	4,00E+05
O95373	Importin-7	b.t.	b.t.	b.t.	3,00E+06	3,00E+06	3,90E+06	b.t.	b.t.	b.t.	b.t.	b.t.	b.t.	3,06E+06	3,40E+06	3,23E+06	b.t.	b.t.	b.t.
O95861	3'(2'),5'-bisphosphate nucleotidase 1	b.t.	b.t.	b.t.	2,64E+06	2,42E+06	2,20E+06	b.t.	b.t.	b.t.	b.t.	b.t.	b.t.	b.t.	b.t.	b.t.	b.t.	b.t.	b.t.
O95994	Anterior gradient protein 2	b.t.	b.t.	b.t.	1,13E+07	1,03E+07	9,40E+06	2,31E+07	2,20E+06	2,20E+07	5,30E+06	9,00E+06	7,40E+06	8,10E+07	9,00E+07	8,55E+07	1,60E+08	3,30E+06	2,80E+06
P00338	L-lactate dehydrogenase A	2,00E+06	2,00E+06	3,00E+06	8,14E+06	9,70E+06	4,70E+06	6,23E+06	6,60E+05	5,90E+06	3,70E+06	1,10E+06	5,20E+07	1,17E+08	1,30E+08	1,24E+08	1,50E+08	8,90E+06	3,10E+06
P00492	Hypoxanthine-guanine phosphoribosyltransferase	b.t.	b.t.	b.t.	6,48E+06	5,94E+06	5,40E+06	2,10E+06	1,75E+06	1,40E+06	2,90E+06	4,35E+06	2,90E+06	b.t.	b.t.	b.t.	1,20E+07	1,08E+07	8,64E+06
P00505	Aspartate aminotransferase, mitochondrial	1,40E+06	2,10E+06	1,40E+06	6,30E+05	9,90E+06	1,40E+07	b.t.	b.t.	b.t.	3,10E+06	3,80E+06	5,70E+06	2,97E+07	3,30E+07	3,14E+07	2,40E+07	4,90E+07	2,40E+07
P00558	Phosphoglycerate kinase 1	5,15E+06	6,30E+06	4,00E+06	1,60E+06	1,40E+08	6,90E+07	1,96E+07	9,20E+06	1,50E+07	7,80E+07	7,30E+07	5,70E+07	4,86E+08	5,40E+08	5,13E+08	1,90E+08	3,30E+08	3,10E+08
P00966	Argininosuccinate synthase	b.t.	b.t.	b.t.	b.t.	b.t.	b.t.	b.t.	b.t.	b.t.	b.t.	b.t.	b.t.	2,88E+06	3,20E+06	3,04E+06	3,80E+06	5,00E+06	4,80E+06
P01375	Tumor necrosis factor α	b.t.	b.t.	b.t.	4,21E+06	5,20E+06	2,30E+06	7,85E+07	3,70E+07	1,20E+07	b.t.	b.t.	b.t.	6,66E+05	7,40E+05	7,03E+05	4,20E+06	3,78E+06	1,00E+07
P02533	Keratin, type I cytoskeletal 14	b.t.	b.t.	b.t.	3,95E+06	4,40E+06	2,50E+06	b.t.	b.t.	b.t.	b.t.	b.t.	b.t.	b.t.	b.t.	b.t.	6,84E+06	6,16E+06	5,70E+06
P02765	Alpha-2-HS-glycoprotein	b.t.	b.t.	b.t.	8,60E+05	8,60E+05	1,12E+06	b.t.	b.t.	b.t.	b.t.	b.t.	b.t.	4,77E+06	5,30E+06	5,04E+06	9,71E+06	7,10E+06	8,80E+06
P03886	NADH-ubiquinone oxidoreductase	4,90E+05	4,90E+05	7,35E+05	b.t.	b.t.	b.t.	b.t.	b.t.	b.t.	b.t.	b.t.	b.t.	8,91E+06	9,90E+06	9,41E+06	1,10E+06	2,30E+07	1,20E+07
P04040	Catalase	b.t.	b.t.	b.t.	1,13E+07	1,03E+07	9,40E+06	2,31E+07	2,20E+06	2,20E+07	5,30E+06	9,00E+06	7,40E+06	8,10E+07	9,00E+07	8,55E+07	1,60E+08	3,30E+06	2,80E+06
P04083	Annexin A1	2,73E+06	4,90E+06	5,60E+05	2,00E+07	1,20E+07	2,00E+07	4,80E+06	1,00E+06	4,30E+06	1,24E+07	6,80E+06	1,80E+07	6,21E+07	6,90E+07	6,56E+07	3,70E+07	3,33E+07	5,70E+06
P04179	Superoxide dismutase [Mn], mitochondrial	1,00E+06	1,00E+06	1,50E+06	4,40E+05	1,80E+07	1,00E+07	4,50E+06	3,75E+06	3,00E+06	2,40E+07	3,60E+07	2,40E+07	5,49E+07	6,10E+07	5,80E+07	3,50E+07	3,30E+06	2,80E+06
P04264	Keratin, type II cytoskeletal 1	6,25E+06	1,00E+07	2,50E+06	4,50E+06	1,20E+08	2,60E+07	3,90E+06	1,20E+06	3,30E+06	5,90E+06	6,00E+06	1,20E+07	1,80E+08	2,00E+08	1,90E+08	2,90E+07	2,80E+07	9,10E+07
P04406	Glyceraldehyde-3-phosphate dehydrogenase	5,80E+06	3,70E+06	7,90E+06	1,09E+08	2,10E+07	1,40E+08	3,17E+07	5,40E+06	2,90E+07	8,60E+06	6,00E+07	2,90E+08	4,05E+08	4,50E+08	4,28E+08	5,10E+08	1,10E+07	4,10E+07
P05120	Plasminogen activator inhibitor 2	2,20E+06	2,20E+06	3,30E+06	1,16E+07	1,30E+07	7,30E+06	6,30E+06	5,25E+06	4,20E+06	3,20E+06	3,40E+06	5,30E+06	3,33E+07	3,70E+07	3,52E+07	2,00E+07	1,20E+07	2,30E+07
P05198	Eukaryotic translation initiation factor 2 subunit 1	b.t.	b.t.	b.t.	1,16E+07	1,07E+07	9,70E+06	b.t.	b.t.	b.t.	3,60E+06	5,40E+06	3,60E+06	1,26E+07	1,40E+07	1,33E+07	1,60E+07	1,44E+07	1,15E+07
P05783	Keratin, type I cytoskeletal 18	b.t.	b.t.	b.t.	3,14E+06	3,20E+06	2,20E+06	b.t.	b.t.	b.t.	1,90E+06	2,70E+06	1,50E+06	8,01E+06	8,90E+06	8,46E+06	1,00E+07	1,70E+07	1,30E+07
P05787	Keratin, type II cytoskeletal 8	b.t.	b.t.	b.t.	4,92E+06	4,51E+06	4,10E+06	3,00E+06	2,50E+06	2,00E+06	6,10E+06	3,40E+06	8,80E+06	3,15E+07	3,50E+07	3,33E+07	2,20E+07	1,10E+07	1,20E+07
P06703	Protein S100-A6	b.t.	b.t.	b.t.	b.t.	b.t.	b.t.	b.t.	b.t.	b.t.	1,90E+06	2,85E+06	1,90E+06	6,30E+06	7,00E+06	6,65E+06	1,90E+07	1,71E+07	1,37E+07
P06733	Alpha-enolase	1,31E+07	2,10E+07	5,20E+06	8,20E+06	2,50E+08	1,40E+08	1,86E+07	5,20E+06	1,60E+07	6,50E+07	8,50E+07	1,70E+08	5,40E+08	6,00E+08	5,70E+08	2,90E+08	4,40E+08	4,10E+08
P06744	Glucose-6-phosphate isomerase	2,00E+06	2,00E+06	3,00E+06	3,28E+07	3,90E+07	1,90E+07	4,50E+06	1,40E+06	3,80E+06	3,90E+06	7,40E+06	1,90E+07	7,47E+07	8,30E+07	7,89E+07	4,00E+07	6,70E+07	7,20E+07
P06899	Histone H2B type 1-J	3,40E+06	5,10E+06	3,40E+06	b.t.	b.t.	b.t.	4,50E+06	3,75E+06	3,00E+06	1,30E+07	1,95E+07	1,30E+07	b.t.	b.t.	b.t.	1,90E+07	1,71E+07	1,37E+07
P07711	Cathepsin L1	b.t.	b.t.	b.t.	b.t.	b.t.	b.t.	b.t.	b.t.	b.t.	1,10E+06	1,65E+06	2,20E+06	2,07E+06	2,30E+06	2,19E+06	b.t.	b.t.	b.t.
P07741	Adenine phosphoribosyltransferase	b.t.	b.t.	b.t.	2,16E+06	1,98E+06	1,80E+06	b.t.	b.t.	b.t.	5,70E+06	8,55E+06	5,70E+06	7,20E+06	8,00E+06	7,60E+06	9,70E+06	2,60E+05	2,08E+05
P07814	Bifunctional glutamate/proline-tRNA ligase	b.t.	b.t.	b.t.	1,52E+06	5,20E+05	1,80E+06	b.t.	b.t.	b.t.	b.t.	b.t.	b.t.	4,32E+06	4,80E+06	4,56E+06	4,30E+06	6,50E+06	7,20E+06
P07900	Heat shock protein HSP 90-alpha	2,10E+05	1,30E+05	2,90E+05	1,08E+08	1,20E+08	1,14E+08	6,60E+07	5,60E+07	9,30E+07	4,60E+05	1,50E+05	2,90E+05	2,20E+06	1,20E+07	1,20E+07	3,30E+06	2,20E+06	2,20E+06
P07954	Fumarate hydratase, mitochondrial	b.t.	b.t.	b.t.	4,56E+06	4,18E+06	3,80E+06	2,10E+06	1,75E+06	1,40E+06	b.t.	b.t.	b.t.	2,07E+07	2,30E+07	2,19E+07	9,00E+06	5,70E+06	7,70E+06
P08174	Complement decay-accelerating factor	b.t.	b.t.	b.t.	b.t.	b.t.	b.t.	b.t.	b.t.	b.t.	2,50E+06	1,10E+06	3,90E+06	9,90E+06	1,10E+07	1,05E+07	1,20E+07	1,00E+07	1,60E+07
P08238	Heat shock protein HSP 90-beta	b.t.	b.t.	b.t.	7,11E+07	7,90E+07	7,51E+07	5,40E+07	2,70E+07	7,00E+07	1,30E+05	8,10E+05	1,90E+05	1,15E+07	1,10E+07	8,60E+06	3,05E+06	1,30E+06	2,40E+06
P08727	Keratin, type I cytoskeletal 19	b.t.	b.t.	b.t.	2,69E+06	3,70E+06	1,20E+06	b.t.	b.t.	b.t.	1,30E+06	2,40E+06	1,50E+06	1,44E+07	1,60E+07	1,52E+07	3,00E+07	9,30E+06	5,70E+06
P08779	Keratin, type I cytoskeletal 16	b.t.	b.t.	b.t.	4,50E+06	4,50E+06	5,85E+06	b.t.	b.t.	b.t.	b.t.	b.t.	b.t.	b.t.	b.t.	b.t.	b.t.	b.t.	b.t.
P09382	Galectin-1	b.t.	b.t.	b.t.	2,40E+06	2,20E+06	2,00E+06	b.t.	b.t.	b.t.	b.t.	b.t.	b.t.	1,26E+07	1,40E+07	1,33E+07	2,30E+07	2,07E+07	1,66E+07
P09622	Dihydrolipoyl dehydrogenase, mitochondrial	b.t.	b.t.	b.t.	b.t.	b.t.	b.t.	b.t.	b.t.	b.t.	b.t.	b.t.	b.t.	b.t.	b.t.	b.t.	4,40E+06	3,96E+06	4,30E+06
P09661	U2 small nuclear ribonucleoprotein A	b.t.	b.t.	b.t.	4,56E+06	4,18E+06	3,80E+06	b.t.	b.t.	b.t.	b.t.	b.t.	b.t.	2,52E+06	2,80E+06	2,66E+06	b.t.	b.t.	b.t.
P09874	Poly [ADP-ribose] polymerase 1	b.t.	b.t.	b.t.	3,36E+06	3,08E+06	2,80E+06	b.t.	b.t.	b.t.	b.t.	b.t.	b.t.	6,03E+06	6,70E+06	6,37E+06	4,10E+06	3,69E+06	5,10E+06
P09960	A-4 hydrolase	b.t.	b.t.	b.t.	3,10E+06	3,10E+06	4,03E+06	b.t.	b.t.	b.t.	b.t.	b.t.	b.t.	7,83E+06	8,70E+06	8,27E+06	5,00E+06	1,30E+07	1,10E+07
P0DMV9	Heat shock 70 kDa protein 1B	2,35E+06	3,00E+06	1,70E+06	1,50E+06	5,00E+07	2,70E+07	1,85E+06	1,10E+06	1,30E+06	1,30E+07	9,00E+06	2,00E+07	7,74E+07	8,60E+07	8,17E+07	3,30E+07	8,60E+07	7,40E+07
P10155	60 kDa SS-A/Ro ribonucleoprotein	b.t.	b.t.	b.t.	3,12E+06	3,30E+06	2,10E+06	b.t.	b.t.	b.t.	b.t.	b.t.	b.t.	4,50E+06	5,00E+06	4,75E+06	2,50E+06	2,25E+06	1,00E+07

P11169	Solute carrier family 2, facilitated glucose transporter member 3	b.t.	b.t.	b.t.	3,90E+06	3,90E+06	5,07E+06	b.t.	b.t.	b.t.	b.t.	b.t.	b.t.	2,97E+06	3,30E+06	3,14E+06	7,20E+06	6,48E+06	6,00E+06
P11279	Lysosome-associated membrane glycoprotein 1	b.t.	b.t.	b.t.	3,40E+06	3,40E+06	4,42E+06	b.t.	b.t.	b.t.	b.t.	b.t.	b.t.	b.t.	b.t.	b.t.	b.t.	b.t.	b.t.
P11413	Glucose-6-phosphate 1-dehydrogenase	2,25E+06	1,70E+06	2,80E+06	2,00E+06	1,90E+07	2,50E+07	2,39E+07	3,70E+06	2,20E+07	8,80E+06	1,70E+07	2,20E+07	1,44E+08	1,60E+08	1,52E+08	7,90E+07	1,00E+08	1,20E+08
P11940	Polyadenylate-binding protein 1	b.t.	b.t.	b.t.	b.t.	b.t.	b.t.	b.t.	b.t.	b.t.	2,30E+06	2,30E+06	3,45E+06	1,44E+07	1,60E+07	1,52E+07	5,90E+06	7,70E+06	1,50E+07
P12081	Histidine--tRNA ligase, cytoplasmic	b.t.	b.t.	b.t.	4,30E+06	4,30E+06	5,59E+06	b.t.	b.t.	b.t.	9,30E+05	9,30E+05	1,40E+06	1,17E+07	1,30E+07	1,24E+07	7,80E+06	8,10E+06	8,40E+06
P12268	Inosine-5'-monophosphate dehydrogenase 2	b.t.	b.t.	b.t.	5,28E+06	4,84E+06	4,40E+06	b.t.	b.t.	b.t.	2,95E+06	1,90E+06	4,00E+06	1,26E+07	1,40E+07	1,33E+07	9,30E+06	9,90E+06	1,20E+07
P12814	Alpha-actinin-1	6,50E+05	9,75E+05	6,50E+05	8,29E+06	4,40E+06	8,70E+06	b.t.	b.t.	b.t.	7,50E+05	7,50E+05	1,13E+06	1,80E+07	2,00E+07	1,90E+07	6,70E+06	3,90E+06	1,40E+07
P12931	Proto-oncogene tyrosine-protein kinase Src	b.t.	b.t.	b.t.	b.t.	b.t.	b.t.	b.t.	b.t.	b.t.	b.t.	b.t.	b.t.	b.t.	b.t.	b.t.	3,80E+06	3,30E+06	5,00E+06
P13010	X-ray repair cross-complementing protein 5	b.t.	b.t.	b.t.	1,26E+07	1,70E+07	5,80E+06	b.t.	b.t.	b.t.	3,25E+06	2,70E+06	3,80E+06	1,71E+07	1,90E+07	1,81E+07	1,80E+07	2,30E+07	2,30E+07
P13489	Ribonuclease inhibitor	b.t.	b.t.	b.t.	5,40E+06	4,95E+06	4,50E+06	b.t.	b.t.	b.t.	1,50E+06	1,50E+06	2,25E+06	6,93E+06	7,70E+06	7,32E+06	3,40E+06	3,06E+06	1,10E+07
P13639	Elongation factor 2	2,36E+06	3,80E+06	9,10E+05	2,53E+07	1,70E+07	2,40E+07	8,65E+06	1,30E+06	8,00E+06	4,90E+06	5,90E+06	2,40E+07	8,01E+07	8,90E+07	8,46E+07	3,50E+07	7,90E+07	3,10E+07
P13645	Keratin, type I cytoskeletal 10	2,73E+06	4,50E+06	9,60E+05	2,00E+06	7,30E+07	1,00E+07	1,11E+07	9,25E+06	7,40E+06	2,40E+06	2,40E+06	8,20E+06	6,84E+07	7,60E+07	7,22E+07	1,30E+07	1,80E+07	4,50E+07
P13647	Keratin, type II cytoskeletal 5	9,10E+05	9,10E+05	1,37E+06	b.t.	b.t.	b.t.	b.t.	b.t.	b.t.	b.t.	b.t.	b.t.	b.t.	b.t.	b.t.	9,00E+05	3,00E+06	1,10E+07
P13667	Protein disulfide-isomerase A4	b.t.	b.t.	b.t.	1,25E+07	1,30E+07	8,60E+06	b.t.	b.t.	b.t.	2,50E+06	1,40E+06	3,60E+06	2,43E+07	2,70E+07	2,57E+07	1,80E+07	2,40E+07	3,10E+07
P13674	Prolyl 4-hydroxylase subunit alpha-1	b.t.	b.t.	b.t.	b.t.	b.t.	b.t.	b.t.	b.t.	b.t.	b.t.	b.t.	b.t.	3,15E+07	3,50E+07	3,33E+07	1,10E+07	9,90E+06	7,92E+06
P13693	Translationally-controlled tumor protein	b.t.	b.t.	b.t.	b.t.	b.t.	b.t.	b.t.	b.t.	b.t.	4,30E+06	6,45E+06	4,30E+06	6,39E+06	7,10E+06	6,75E+06	8,30E+06	7,47E+06	5,98E+06
P13804	Electron transfer flavoprotein subunit alpha, mitochondrial	b.t.	b.t.	b.t.	3,60E+06	3,30E+06	3,00E+06	b.t.	b.t.	b.t.	b.t.	b.t.	b.t.	2,25E+07	2,50E+07	2,38E+07	5,04E+06	4,54E+06	4,20E+06
P14550	Alcohol dehydrogenase [NADP(+)]	b.t.	b.t.	b.t.	2,50E+06	2,50E+06	3,25E+06	b.t.	b.t.	b.t.	3,50E+06	5,25E+06	3,50E+06	3,69E+06	4,10E+06	3,90E+06	5,10E+06	4,59E+06	3,60E+05
P14625	Endoplasmin	2,40E+06	2,40E+06	3,60E+06	2,00E+06	9,40E+06	2,20E+07	3,15E+06	1,70E+06	2,30E+06	6,00E+06	1,30E+07	1,00E+07	1,17E+08	1,30E+08	1,24E+08	6,70E+07	9,60E+07	1,10E+08
P14866	Heterogeneous nuclear ribonucleoprotein L	b.t.	b.t.	b.t.	3,84E+06	3,52E+06	3,20E+06	b.t.	b.t.	b.t.	3,60E+06	5,40E+06	3,60E+06	1,08E+07	1,20E+07	1,14E+07	2,00E+07	1,80E+07	1,44E+07
P14868	Aspartate-tRNA ligase, cytoplasmic	b.t.	b.t.	b.t.	b.t.	b.t.	b.t.	b.t.	b.t.	b.t.	b.t.	b.t.	b.t.	8,91E+06	9,90E+06	9,41E+06	3,90E+06	3,51E+06	1,10E+07
P15121	Aldose reductase	b.t.	b.t.	b.t.	b.t.	b.t.	b.t.	b.t.	b.t.	b.t.	6,80E+06	1,02E+07	6,80E+06	1,44E+07	1,60E+07	1,52E+07	1,50E+07	1,35E+07	1,08E+07
P15170	Eukaryotic peptide chain release factor GTP-binding subunit ERF3A	b.t.	b.t.	b.t.	b.t.	b.t.	b.t.	b.t.	b.t.	b.t.	b.t.	b.t.	b.t.	b.t.	b.t.	b.t.	4,40E+06	1,20E+07	8,60E+06
P15559	NAD(P)H dehydrogenase [quinone] 1	6,65E+06	6,60E+06	6,70E+06	3,28E+06	3,20E+06	2,40E+06	4,40E+06	2,20E+06	3,30E+06	1,74E+07	7,50E+05	3,40E+07	4,50E+07	5,00E+07	4,75E+07	8,90E+07	6,90E+06	4,80E+06
P15880	40S ribosomal protein S2	b.t.	b.t.	b.t.	1,68E+06	1,54E+06	1,40E+06	b.t.	b.t.	b.t.	5,40E+06	8,10E+06	5,40E+06	1,17E+07	1,30E+07	1,24E+07	1,30E+07	1,17E+07	8,60E+05
P16083	Ribosyldihydronicotinamide dehydrogenase [quinone]	b.t.	b.t.	b.t.	b.t.	b.t.	b.t.	b.t.	b.t.	b.t.	2,70E+06	4,05E+06	2,70E+06	b.t.	b.t.	b.t.	5,50E+06	4,95E+06	3,96E+06
P16152	Carbonyl reductase [NADPH] 1	b.t.	b.t.	b.t.	6,72E+06	6,16E+06	5,60E+06	1,65E+06	1,38E+06	1,10E+06	7,70E+06	1,16E+07	7,70E+06	4,86E+07	5,40E+07	5,13E+07	2,20E+07	1,98E+07	1,40E+06
P16402	Histone H1.3	b.t.	b.t.	b.t.	b.t.	b.t.	b.t.	b.t.	b.t.	b.t.	1,40E+07	2,10E+07	1,40E+07	3,33E+07	3,70E+07	3,52E+07	3,10E+07	2,79E+07	2,23E+07
P16403	Histone H1.2	b.t.	b.t.	b.t.	b.t.	b.t.	b.t.	b.t.	b.t.	b.t.	1,40E+07	2,10E+07	1,40E+07	3,33E+07	3,70E+07	3,52E+07	3,10E+07	2,79E+07	2,23E+07
P16930	Fumarylacetoacetase	b.t.	b.t.	b.t.	3,00E+06	3,00E+06	3,90E+06	b.t.	b.t.	b.t.	b.t.	b.t.	b.t.	b.t.	b.t.	b.t.	1,20E+06	1,08E+06	8,64E+05
P17174	Aspartate aminotransferase, cytoplasmic	b.t.	b.t.	b.t.	3,69E+06	2,20E+06	3,70E+06	2,25E+06	1,88E+06	1,50E+06	6,40E+05	6,40E+05	9,60E+05	6,75E+06	7,50E+06	7,13E+06	3,10E+06	1,20E+07	3,60E+06
P17655	Calpain-2 catalytic subunit	b.t.	b.t.	b.t.	2,64E+06	2,42E+06	2,20E+06	b.t.	b.t.	b.t.	b.t.	b.t.	b.t.	5,13E+06	5,70E+06	5,42E+06	3,20E+06	2,88E+06	3,90E+06
P17987	T-complex protein 1 subunit alpha	b.t.	b.t.	b.t.	b.t.	b.t.	b.t.	b.t.	b.t.	b.t.	b.t.	b.t.	b.t.	1,26E+07	1,40E+07	1,33E+07	4,80E+06	4,32E+06	6,50E+06
P18124	60S ribosomal protein L7	b.t.	b.t.	b.t.	4,32E+06	3,96E+06	3,60E+06	2,20E+06	1,20E+06	1,60E+06	7,30E+06	1,10E+07	7,30E+06	1,53E+07	1,70E+07	1,62E+07	2,20E+07	1,98E+07	1,58E+07
P19367	Hexokinase-1	b.t.	b.t.	b.t.	2,50E+06	2,50E+06	3,25E+06	b.t.	b.t.	b.t.	b.t.	b.t.	b.t.	b.t.	b.t.	b.t.	7,40E+05	4,80E+06	3,84E+06
P19623	Spermidine synthase	b.t.	b.t.	b.t.	b.t.	b.t.	b.t.	b.t.	b.t.	b.t.	b.t.	b.t.	b.t.	b.t.	b.t.	b.t.	1,20E+07	1,08E+07	8,64E+06
P19838	Nuclear factor NFκB p105	1,70E+05	1,70E+05	2,55E+05	1,13E+07	5,70E+07	1,20E+07	7,65E+07	6,38E+07	5,10E+07	1,90E+05	6,40E+05	1,90E+05	6,93E+07	7,70E+06	7,32E+06	4,10E+06	2,40E+06	1,30E+06
P20073	Annexin A7	b.t.	b.t.	b.t.	b.t.	b.t.	b.t.	b.t.	b.t.	b.t.	b.t.	b.t.	b.t.	7,11E+06	7,90E+06	7,51E+06	1,21E+07	1,50E+07	6,50E+06
P20337	Ras-related protein Rab-3B	b.t.	b.t.	b.t.	2,88E+06	2,64E+06	2,40E+06	4,05E+06	3,38E+06	2,70E+06	3,70E+06	5,55E+06	3,70E+06	b.t.	b.t.	b.t.	1,40E+07	1,26E+07	1,01E+07
P20618	Proteasome subunit beta type-1	3,30E+05	3,30E+05	4,95E+05	2,04E+06	1,87E+06	1,70E+06	b.t.	b.t.	b.t.	3,60E+06	5,40E+06	3,60E+06	8,46E+06	9,40E+06	8,93E+06	9,00E+06	8,10E+06	5,10E+05
P20700	Lamin-B1	b.t.	b.t.	b.t.	b.t.	b.t.	b.t.	b.t.	b.t.	b.t.	b.t.	b.t.	b.t.	b.t.	b.t.	b.t.	7,70E+06	6,93E+06	5,54E+06
P21333	Filamin-A	1,70E+06	1,70E+06	2,55E+06	4,78E+06	5,50E+06	2,90E+06	b.t.	b.t.	b.t.	1,90E+06	2,30E+06	1,50E+06	1,26E+07	1,40E+07	1,33E+07	7,00E+06	6,00E+06	1,70E+07
P21796	Voltage-dependent anion-selective channel protein 1	b.t.	b.t.	b.t.	b.t.	b.t.	b.t.	b.t.	b.t.	b.t.	1,70E+06	2,55E+06	1,70E+06	2,07E+07	2,30E+07	2,19E+07	2,20E+07	1,98E+07	1,58E+07
P22087	rRNA 2'-O-methyltransferase fibrillarin	b.t.	b.t.	b.t.	b.t.	b.t.	b.t.	b.t.	b.t.	b.t.	b.t.	b.t.	b.t.	9,90E+06	1,10E+07	1,05E+07	1,20E+07	1,08E+07	8,64E+06
P22102	Trifunctional purine biosynthetic protein adenosine-3	b.t.	b.t.	b.t.	2,80E+06	2,80E+06	3,64E+06	b.t.	b.t.	b.t.	5,90E+05	8,85E+05	5,90E+05	2,79E+06	3,10E+06	2,95E+06	1,30E+06	5,60E+06	4,40E+06
P22626	Heterogeneous nuclear ribonucleoproteins A2/B1	b.t.	b.t.	b.t.	8,40E+06	7,70E+06	7,00E+06	7,80E+06	6,50E+06	5,20E+06	6,50E+06	2,70E+06	1,20E+07	6,93E+07	7,70E+07	7,32E+07	2,50E+07	4,20E+06	1,30E+06
P23246	Splicing factor, proline- and glutamine-rich	b.t.	b.t.	b.t.	2,52E+06	2,31E+06	2,10E+06	2,40E+06	2,00E+06	1,60E+06	1,33E+06	1,70E+06	9,60E+05	4,86E+06	5,40E+06	5,13E+06	7,90E+06	7,11E+06	5,69E+06
P23284	Peptidyl-prolyl cis-trans isomerase B	b.t.	b.t.	b.t.	9,70E+05	3,20E+06	1,90E+07	6,55E+06	6,10E+06	3,50E+06	1,70E+07	2,55E+07	1,70E+07	7,11E+07	7,90E+07	7,51E+07	4,90E+07	4,41E+07	1,10E+06
P23368	NAD-dependent malic enzyme, mitochondrial	b.t.	b.t.	b.t.	2,60E+06	2,60E+06	3,38E+06	b.t.	b.t.	b.t.	4,50E+05	4,50E+05	6,75E+05	b.t.	b.t.	b.t.	b.t.	b.t.	b.t.
P23381	Tryptophan-tRNA ligase, cytoplasmic	1,50E+06	2,25E+06	1,50E+06	2,02E+07	1,80E+07	1,60E+07	5,85E+06	4,88E+06	3,90E+06	5,50E+06	7,70E+06	1,30E+07	7,92E+07	8,80E+07	8,36E+07	2,40E+07	5,20E+07	5,60E+07
P23396	40S ribosomal protein S3	b.t.	b.t.	b.t.	1,68E+06	1,54E+06	1,40E+06	b.t.	b.t.	b.t.	3,34E+06	4,70E+05	6,20E+06	1,53E+07	1,70E+07	1,62E+07	4,90E+07	4,41E+07	3,53E+07
P23526	Adenosylhomocysteinase	1,50E+06	1,50E+06	2,25E+06	2,40E+07	2,70E+07	1,50E+07	b.t.	b.t.	b.t.	2,20E+06	2,00E+06	2,40E+06	2,88E+07	3,20E+07	3,04E+07	1,70E+07	5,90E+07	4,20E+07
P23527	Histone H2B type 1-O	3,40E+06	5,10E+06	3,40E+06	b.t.	b.t.	b.t.	4,50E+06	3,75E+06	3,00E+06	1,30E+07	1,95E+07	1,30E+07	b.t.	b.t.	b.t.	1,90E+07	1,71E+07	1,37E+07
P24752	Acetyl-CoA acetyltransferase, mitochondrial	b.t.	b.t.	b.t.	3,12E+06	2,86E+06	2,60E+06	b.t.	b.t.	b.t.	1,80E+06	1,80E+06	2,70E+06	3,06E+06	3,40E+06	3,23E+06	2,20E+06	2,20E+06	1,76E+06
P25398	40S ribosomal protein S12	b.t.	b.t.	b.t.	b.t.	b.t.	b.t.	b.t.	b.t.	b.t.	1,60E+07	2,40E+07	1,60E+07	1,62E+07	1,80E+07	1,71E+07	3,10E+07	2,79E+07	2,23E+07
P25786	Proteasome subunit alpha type-1	b.t.	b.t.	b.t.	5,61E+06	8,70E+06	1,80E+06	b.t.	b.t.	b.t.	1,10E+06	1,65E+06	1,10E+06	1,62E+07	1,80E+07	1,71E+07	2,60E+07	2,34E+07	1,87E+07
P25788	Proteasome subunit alpha type-3	b.t.	b.t.	b.t.	b.t.	b.t.	b.t.	b.t.	b.t.	b.t.	5,30E+06	7,95E+06	5,30E+06	7,74E+06	8,60E+06	8,17E+06	1,60E+07	1,44E+07	1,15E+07
P25789	Proteasome subunit alpha type-4	b.t.	b.t.	b.t.	7,56E+06	6,93E+06	6,30E+06	3,75E+06	3,13E+06	2,50E+06	1,10E+07	1,65E+07	1,10E+07	2,52E+07	2,80E+07	2,66E+07	2,40E+07	2,16E+07	1,73E+07
P26038	Moesin	1,20E+07	1,20E+07	1,80E+07	8,46E+06	7,40E+06	6,80E+06	7,95E+06	6,63E+06	5,30E+06	5,00E+06	2,80E+06	7,20E+06	4,32E+07	4,80E+07	4,56E+07	2,20E+07	1,98E+07	1,10E+07
P26447	Protein S100-A4	b.t.	b.t.	b.t.	9,96E+06	9,13E+06	8,30E+06	4,95E+05	4,13E+05	3,30E+05	2,60E+06	1,30E+06	3,90E+06	1,98E+07	2,20E+07	2,09E+07	5,10E+07	4,59E+07	3,67E+07
P26639	Threonine-tRNA ligase, cytoplasmic	b.t.	b.t.	b.t.	4,52E+06	5,40E+06	2,60E+06	b.t.	b.t.	b.t.	4,70E+05	4,70E+05	7,05E+05	7,74E+06	8,60E+06	8,17E+06	4,10E+06	6,70E+06	6,30E+06
P26641	Elongation factor 1-gamma	1,47E+06	9,30E+05	2,00E+06	2,22E+07	2,90E+07	1,10E+07	2,98E+06	9,60E+05	2,50E+06	1,10E+06	6,60E+05	1,00E+07	3,69E+07	4,10E+07	3,90E+07	4,50E+07	4,05E+07	4,80E+06
P27348	14-3-3 protein theta	b.t.	b.t.	b.t.	4,87E+06	4,00E+06	4,10E+06	9,95E+06	3,70E+06	8,10E+06	6,20E+06	9,20E+06	6,00E+06	7,02E+06	7,80E+06	7,41E+06	5,29E+06	1,90E+06	6,20E+06
P27635	60S ribosomal protein L10	b.t.	b.t.	b.t.	b.t.	b.t.	b.t.	b.t.	b.t.	b.t.	5,10E+06	7,65E+06	5,10E+06	4,68E+06	5,20E+06	4,94E+06	6,10E+06	5,49E+06	4,39E+06

P27695	DNA-(apurinic or apyrimidinic site) lyase	4,20E+05	4,20E+05	6,30E+05	8,28E+06	7,59E+06	6,90E+06	7,35E+06	6,13E+06	4,90E+06	2,20E+06	2,20E+06	3,30E+06	9,90E+06	1,10E+07	1,05E+07	6,80E+06	6,12E+06	4,90E+06
P27708	CAD protein	b.t.	b.t.	b.t.	3,30E+06	3,30E+06	4,29E+06	b.t.	b.t.	b.t.	b.t.	b.t.	b.t.	b.t.	b.t.	b.t.	2,10E+06	6,50E+06	4,00E+06
P28070	Proteasome subunit beta type-4	7,50E+05	7,50E+05	1,13E+06	1,10E+07	1,01E+07	9,20E+06	b.t.	b.t.	b.t.	1,00E+07	1,50E+07	1,00E+07	8,55E+06	9,50E+06	9,03E+06	1,00E+07	9,00E+06	7,20E+06
P28074	Proteasome subunit beta type-5	b.t.	b.t.	b.t.	b.t.	b.t.	b.t.	9,90E+05	8,25E+05	6,60E+05	4,10E+06	6,15E+06	4,10E+06	b.t.	b.t.	b.t.	1,10E+07	9,90E+06	7,92E+06
P28838	Cytosol aminopeptidase	b.t.	b.t.	b.t.	2,90E+06	4,40E+06	1,00E+06	b.t.	b.t.	b.t.	b.t.	b.t.	b.t.	b.t.	b.t.	b.t.	8,50E+06	7,65E+06	3,50E+06
P29401	Transketolase	3,05E+06	4,80E+06	1,30E+06	6,35E+07	6,40E+07	4,50E+07	9,30E+06	7,75E+06	6,20E+06	4,20E+06	1,30E+07	1,90E+07	9,90E+07	1,10E+08	1,05E+08	8,40E+07	5,30E+07	9,70E+07
P29692	Elongation factor 1-delta	b.t.	b.t.	b.t.	b.t.	b.t.	b.t.	b.t.	b.t.	b.t.	2,09E+06	2,80E+05	3,90E+06	b.t.	b.t.	b.t.	6,10E+06	5,49E+06	1,30E+06
P30040	Endoplasmic reticulum resident protein 29	b.t.	b.t.	b.t.	2,68E+06	2,70E+06	1,90E+06	b.t.	b.t.	b.t.	b.t.	b.t.	b.t.	1,89E+07	2,10E+07	2,00E+07	b.t.	b.t.	b.t.
P30043	Flavin reductase (NADPH)	b.t.	b.t.	b.t.	5,04E+06	4,62E+06	4,20E+06	b.t.	b.t.	b.t.	4,10E+06	6,15E+06	4,10E+06	8,82E+06	9,80E+06	9,31E+06	7,80E+06	7,02E+06	5,62E+06
P30044	Peroxiredoxin-5, mitochondrial	b.t.	b.t.	b.t.	2,40E+06	2,20E+06	2,00E+06	b.t.	b.t.	b.t.	b.t.	b.t.	b.t.	9,00E+06	1,00E+07	9,50E+06	1,50E+07	1,35E+07	1,08E+07
P30048	Thioredoxin-dependent peroxide reductase, mitochondrial	3,50E+06	5,25E+06	3,50E+06	1,00E+07	6,00E+06	1,00E+07	4,35E+06	3,63E+06	2,90E+06	3,90E+07	5,85E+07	3,90E+07	3,96E+07	4,40E+07	4,18E+07	3,70E+07	3,33E+07	5,70E+05
P30050	60S ribosomal protein L12	b.t.	b.t.	b.t.	b.t.	b.t.	b.t.	1,29E+06	1,08E+06	8,60E+05	2,60E+07	3,90E+07	2,60E+07	3,60E+07	4,00E+07	3,80E+07	5,10E+07	4,59E+07	3,67E+07
P30084	Enoyl-CoA hydratase, mitochondrial	b.t.	b.t.	b.t.	5,28E+06	4,84E+06	4,40E+06	1,29E+06	1,08E+06	8,60E+05	2,70E+06	2,70E+06	4,05E+06	8,91E+06	9,90E+06	9,41E+06	2,10E+07	1,89E+07	4,20E+05
P30101	Protein disulfide-isomerase A3	5,00E+06	7,50E+06	5,00E+06	2,50E+06	4,50E+07	1,50E+07	9,60E+06	8,00E+06	6,40E+06	1,90E+07	1,90E+07	1,40E+07	1,08E+08	1,20E+08	1,14E+08	4,90E+07	2,70E+07	4,70E+07
P30153	Serine/threonine-protein phosphatase 2A 65 kDa regulatory subunit A	b.t.	b.t.	b.t.	5,78E+06	8,20E+06	2,40E+06	b.t.	b.t.	b.t.	5,60E+06	1,40E+06	4,20E+06	7,02E+06	7,80E+06	7,41E+06	3,10E+06	7,20E+06	1,20E+07
P30838	Aldehyde dehydrogenase. dimeric NADP-preferring	2,15E+06	2,30E+06	2,00E+06	4,27E+07	6,30E+07	1,60E+07	3,90E+06	3,25E+06	2,60E+06	1,20E+07	5,80E+06	1,10E+07	1,53E+08	1,70E+08	1,62E+08	4,50E+07	3,40E+07	1,10E+08
P31150	Rab GDP dissociation inhibitor alpha	b.t.	b.t.	b.t.	6,30E+06	6,30E+06	8,19E+06	b.t.	b.t.	b.t.	b.t.	b.t.	b.t.	7,47E+06	8,30E+06	7,89E+06	1,20E+06	1,08E+06	6,60E+06
P31930	Cytochrome b-c1 complex subunit 1, mitochondrial	b.t.	b.t.	b.t.	b.t.	b.t.	b.t.	b.t.	b.t.	b.t.	b.t.	b.t.	b.t.	1,17E+07	1,30E+07	1,24E+07	8,88E+06	7,99E+06	7,40E+06
P31939	Bifunctional purine biosynthesis protein PURH	b.t.	b.t.	b.t.	3,36E+06	2,10E+06	3,30E+06	b.t.	b.t.	b.t.	2,81E+07	1,10E+06	5,50E+07	2,79E+07	3,10E+07	2,95E+07	5,70E+07	1,70E+07	1,00E+07
P31947	14-3-3 protein sigma	1,60E+06	1,60E+06	2,40E+06	b.t.	b.t.	b.t.	2,10E+06	1,75E+06	1,40E+06	4,10E+06	6,15E+06	4,10E+06	1,26E+07	1,40E+07	1,33E+07	2,10E+07	1,89E+07	2,80E+06
P32929	Cystathionine gamma-lyase	b.t.	b.t.	b.t.	2,80E+06	2,80E+06	3,64E+06	b.t.	b.t.	b.t.	b.t.	b.t.	b.t.	4,86E+06	5,40E+06	5,13E+06	2,52E+06	2,27E+06	2,10E+06
P33778	Histone H2B type 1-B	3,40E+06	5,10E+06	3,40E+06	b.t.	b.t.	b.t.	4,50E+06	3,75E+06	3,00E+06	1,30E+07	1,95E+07	1,30E+07	b.t.	b.t.	b.t.	1,90E+07	1,71E+07	1,37E+07
P34897	Serine hydroxymethyltransferase, mitochondrial	b.t.	b.t.	b.t.	2,88E+06	2,64E+06	2,40E+06	1,98E+06	9,60E+05	1,50E+06	2,75E+06	1,40E+06	4,10E+06	2,16E+07	2,40E+07	2,28E+07	1,30E+07	4,10E+06	1,40E+07
P34931	Heat shock 70 kDa protein 1-like	2,25E+06	3,10E+06	1,40E+06	2,09E+07	3,40E+07	5,50E+06	2,10E+06	1,40E+06	1,40E+06	7,30E+06	6,50E+06	3,90E+06	6,12E+07	6,80E+07	6,46E+07	1,90E+07	2,50E+07	3,80E+07
P34932	Heat shock 70 kDa protein 4	b.t.	b.t.	b.t.	2,95E+06	2,40E+06	2,50E+06	b.t.	b.t.	b.t.	1,40E+06	1,40E+06	1,40E+06	1,35E+07	1,50E+07	1,43E+07	1,10E+07	9,90E+06	2,00E+07
P35268	60S ribosomal protein L22	b.t.	b.t.	b.t.	b.t.	b.t.	b.t.	b.t.	b.t.	b.t.	b.t.	b.t.	b.t.	1,62E+07	1,80E+07	1,71E+07	2,60E+07	2,34E+07	1,87E+07
P35270	Sepiapterin reductase	b.t.	b.t.	b.t.	2,40E+06	2,20E+06	2,00E+06	b.t.	b.t.	b.t.	1,80E+06	2,70E+06	1,80E+06	b.t.	b.t.	b.t.	b.t.	b.t.	b.t.
P35527	Keratin, type I cytoskeletal 9	3,80E+06	6,20E+06	1,40E+06	2,00E+06	8,60E+07	7,10E+06	1,10E+06	9,13E+05	7,30E+05	6,30E+06	2,20E+06	6,20E+06	1,35E+08	1,50E+08	1,43E+08	2,80E+07	5,60E+06	5,30E+07
P35908	Keratin, type II cytoskeletal 2 epidermal	1,80E+06	1,80E+06	2,70E+06	9,60E+05	1,40E+07	3,80E+06	9,30E+05	7,75E+05	6,20E+05	2,45E+06	1,40E+06	3,50E+06	2,43E+07	2,70E+07	2,57E+07	8,30E+06	5,70E+06	1,40E+07
P35998	26S protease regulatory subunit 7	b.t.	b.t.	b.t.	1,44E+06	1,32E+06	1,20E+06	b.t.	b.t.	b.t.	b.t.	b.t.	b.t.	b.t.	b.t.	b.t.	2,30E+06	2,07E+06	1,66E+06
P36952	Serpin B5	b.t.	b.t.	b.t.	6,48E+06	5,94E+06	5,40E+06	b.t.	b.t.	b.t.	2,40E+06	3,60E+06	2,40E+06	b.t.	b.t.	b.t.	b.t.	b.t.	b.t.
P37802	Transgelin-2	b.t.	b.t.	b.t.	3,96E+06	3,63E+06	3,30E+06	3,30E+06	2,75E+06	2,20E+06	b.t.	b.t.	b.t.	7,83E+06	8,70E+06	8,27E+06	b.t.	b.t.	b.t.
P37837	Transaldolase	3,60E+06	5,40E+06	3,60E+06	3,48E+06	3,19E+06	2,90E+06	1,95E+06	1,63E+06	1,30E+06	1,60E+07	2,40E+07	1,60E+07	1,71E+07	1,90E+07	1,81E+07	3,20E+07	2,88E+07	2,30E+07
P38117	Electron transfer flavoprotein subunit beta	b.t.	b.t.	b.t.	b.t.	b.t.	b.t.	b.t.	b.t.	b.t.	4,20E+06	6,30E+06	4,20E+06	b.t.	b.t.	b.t.	1,00E+07	9,00E+06	7,20E+06
P38646	Stress-70 protein, mitochondrial	b.t.	b.t.	b.t.	2,72E+07	3,90E+07	1,10E+07	2,21E+06	8,20E+05	1,80E+06	5,10E+06	9,90E+06	2,00E+07	7,65E+07	8,50E+07	8,08E+07	4,50E+07	1,40E+07	3,90E+07
P39023	60S ribosomal protein L3	b.t.	b.t.	b.t.	b.t.	b.t.	b.t.	b.t.	b.t.	b.t.	7,00E+06	1,05E+07	7,00E+06	7,11E+06	7,90E+06	7,51E+06	2,10E+07	1,89E+07	1,51E+07
P39656	Dolichyl-diphosphooligosaccharide-protein glycosyltransferase 48 kDa	b.t.	b.t.	b.t.	3,90E+06	3,90E+06	5,07E+06	b.t.	b.t.	b.t.	b.t.	b.t.	b.t.	b.t.	b.t.	b.t.	3,20E+06	2,88E+06	5,10E+06
P40227	T-complex protein 1 subunit zeta	b.t.	b.t.	b.t.	2,89E+06	4,10E+06	1,20E+06	2,10E+06	1,75E+06	1,40E+06	1,00E+06	1,00E+06	1,50E+06	9,90E+06	1,10E+07	1,05E+07	8,70E+06	5,10E+06	5,30E+06
P40925	Malate dehydrogenase, cytoplasmic	b.t.	b.t.	b.t.	b.t.	b.t.	b.t.	1,80E+06	1,50E+06	1,20E+06	b.t.	b.t.	b.t.	9,90E+06	1,10E+07	1,05E+07	7,80E+06	7,02E+06	5,62E+06
P40926	Malate dehydrogenase, mitochondrial	b.t.	b.t.	b.t.	1,27E+07	1,50E+06	1,70E+07	4,95E+06	4,13E+06	3,30E+06	2,90E+06	5,30E+05	2,70E+07	3,24E+07	3,60E+07	3,42E+07	2,20E+07	3,70E+06	1,50E+06
P41091	Eukaryotic translation initiation factor 2 subunit 3	b.t.	b.t.	b.t.	5,04E+06	3,50E+06	4,70E+06	b.t.	b.t.	b.t.	b.t.	b.t.	b.t.	7,74E+06	8,60E+06	8,17E+06	4,50E+06	1,30E+07	1,30E+07
P41250	Glycine-tRNA ligase	1,50E+06	2,25E+06	1,50E+06	6,09E+06	9,80E+06	1,70E+06	3,30E+06	2,75E+06	2,20E+06	3,15E+06	1,90E+06	4,40E+06	1,35E+07	1,50E+07	1,43E+07	9,40E+06	1,10E+07	1,50E+07
P41252	Isoleucine-tRNA ligase, cytoplasmic	b.t.	b.t.	b.t.	5,85E+06	6,10E+06	4,00E+06	b.t.	b.t.	b.t.	2,40E+06	3,70E+06	2,60E+06	1,08E+07	1,20E+07	1,14E+07	4,10E+06	9,30E+06	1,30E+07
P43686	26S protease regulatory subunit 6B	b.t.	b.t.	b.t.	b.t.	b.t.	b.t.	b.t.	b.t.	b.t.	b.t.	b.t.	b.t.	7,11E+06	7,90E+06	7,51E+06	3,70E+05	6,10E+06	4,88E+06
P46108	Adapter molecule crk	b.t.	b.t.	b.t.	b.t.	b.t.	b.t.	b.t.	b.t.	b.t.	b.t.	b.t.	b.t.	b.t.	b.t.	b.t.	4,40E+06	3,96E+06	3,17E+06
P46439	Glutathione S-transferase Mu 5	b.t.	b.t.	b.t.	b.t.	b.t.	b.t.	b.t.	b.t.	b.t.	b.t.	b.t.	b.t.	b.t.	b.t.	b.t.	1,80E+07	1,62E+07	1,30E+07
P46779	60S ribosomal protein L28	b.t.	b.t.	b.t.	b.t.	b.t.	b.t.	b.t.	b.t.	b.t.	3,10E+06	4,65E+06	3,10E+06	4,05E+06	4,50E+06	4,28E+06	3,30E+06	2,97E+06	2,38E+06
P46781	40S ribosomal protein S9	1,20E+06	1,80E+06	1,20E+06	8,28E+06	7,59E+06	6,90E+06	1,52E+06	1,30E+06	8,70E+05	1,70E+07	2,55E+07	1,70E+07	2,25E+07	2,50E+07	2,38E+07	3,00E+07	7,90E+06	1,80E+06
P46940	Ras GTPase-activating-like protein IQGAP1	2,10E+06	2,10E+06	3,15E+06	1,56E+07	2,60E+07	3,70E+06	1,95E+06	1,63E+06	1,30E+06	3,40E+06	1,60E+06	1,00E+07	2,07E+07	2,30E+07	2,19E+07	1,20E+07	9,20E+06	2,80E+07
P47756	F-actin-capping protein subunit beta	b.t.	b.t.	b.t.	b.t.	b.t.	b.t.	b.t.	b.t.	b.t.	b.t.	b.t.	b.t.	b.t.	b.t.	b.t.	2,50E+07	2,25E+07	3,20E+05
P47897	Glutamine-tRNA ligase	b.t.	b.t.	b.t.	2,25E+06	2,40E+06	1,50E+06	b.t.	b.t.	b.t.	2,30E+06	3,45E+06	2,30E+06	b.t.	b.t.	b.t.	2,30E+06	2,07E+06	1,66E+06
P49189	4-trimethylaminobutyraldehyde dehydrogenase	b.t.	b.t.	b.t.	b.t.	b.t.	b.t.	1,17E+06	9,75E+05	7,80E+05	4,40E+05	4,40E+05	6,60E+05	b.t.	b.t.	b.t.	3,20E+06	8,30E+06	6,64E+06
P49327	Fatty acid synthase	b.t.	b.t.	b.t.	1,40E+06	2,50E+07	6,00E+06	4,61E+06	6,10E+05	4,30E+06	4,20E+06	3,80E+06	4,60E+06	2,97E+07	3,30E+07	3,14E+07	2,20E+07	2,30E+07	2,40E+07
P49411	Elongation factor Tu, mitochondrial	b.t.	b.t.	b.t.	5,76E+05	5,28E+05	4,80E+05	b.t.	b.t.	b.t.	5,55E+06	1,90E+06	9,20E+06	1,35E+07	1,50E+07	1,43E+07	4,50E+06	4,05E+06	3,30E+06
P49419	Alpha-aminoadipic semialdehyde dehydrogenase	b.t.	b.t.	b.t.	4,73E+06	4,70E+06	3,40E+06	b.t.	b.t.	b.t.	b.t.	b.t.	b.t.	4,32E+06	4,80E+06	4,56E+06	b.t.	b.t.	b.t.
P49588	Alanine--tRNA ligase. cytoplasmic	b.t.	b.t.	b.t.	b.t.	b.t.	b.t.	b.t.	b.t.	b.t.	b.t.	b.t.	b.t.	1,17E+07	1,30E+07	1,24E+07	3,40E+06	3,06E+06	3,30E+06
P49720	Proteasome subunit beta type-3	1,40E+06	1,40E+06	2,10E+06	1,02E+07	1,50E+07	3,80E+06	2,85E+06	2,38E+06	1,90E+06	1,10E+06	1,65E+06	1,10E+06	1,44E+07	1,60E+07	1,52E+07	2,00E+07	1,80E+07	7,00E+05
P49721	Proteasome subunit beta type-2	5,40E+06	5,40E+06	8,10E+06	1,51E+07	1,20E+07	1,30E+07	3,90E+06	3,25E+06	2,60E+06	7,50E+05	2,60E+06	9,30E+06	7,20E+06	8,00E+06	7,60E+06	9,30E+06	8,37E+06	6,70E+06
P49748	Very long-chain specific acyl-CoA dehydrogenase, mitochondrial	b.t.	b.t.	b.t.	2,70E+06	2,70E+06	3,51E+06	b.t.	b.t.	b.t.	b.t.	b.t.	b.t.	6,03E+06	6,70E+06	6,37E+06	4,80E+06	4,32E+06	4,80E+06
P49773	Histidine triad nucleotide-binding protein 1	b.t.	b.t.	b.t.	b.t.	b.t.	b.t.	b.t.	b.t.	b.t.	6,00E+06	9,00E+06	6,00E+06	1,44E+07	1,60E+07	1,52E+07	1,50E+07	1,35E+07	1,08E+07
P50213	Isocitrate dehydrogenase [NAD] subunit alpha, mitochondrial	b.t.	b.t.	b.t.	b.t.	b.t.	b.t.	b.t.	b.t.	b.t.	b.t.	b.t.	b.t.	1,44E+07	1,60E+07	1,52E+07	3,00E+06	2,70E+06	2,16E+06
P50502	Hsc70-interacting protein	b.t.	b.t.	b.t.	b.t.	b.t.	b.t.	b.t.	b.t.	b.t.	1,20E+06	1,20E+06	1,80E+06	5,49E+06	6,10E+06	5,80E+06	4,60E+06	4,14E+06	3,31E+06
P50990	T-complex protein 1 subunit theta	b.t.	b.t.	b.t.	1,32E+06	1,21E+06	1,10E+06	b.t.	b.t.	b.t.	9,20E+05	9,20E+05	1,38E+06	3,60E+06	4,00E+06	3,80E+06	8,70E+06	7,83E+06	5,30E+06
P50991	T-complex protein 1 subunit delta	3,10E+06	3,10E+06	4,65E+06	1,31E+07	1,70E+07	6,60E+06	b.t.	b.t.	b.t.	2,45E+06	9,90E+05	3,90E+06	9,90E+06	1,10E+07	1,05E+07	8,10E+06	5,40E+06	1,00E+07

P51148	Ras-related protein Rab-5C	b.t.	b.t.	b.t.	8,40E+05	7,70E+05	7,00E+05	b.t.	b.t.	b.t.	b.t.	b.t.	b.t.	9,90E+06	1,10E+07	1,05E+07	6,80E+06	6,12E+06	4,90E+06
P51149	Ras-related protein Rab-7a	b.t.	b.t.	b.t.	b.t.	b.t.	b.t.	b.t.	b.t.	b.t.	b.t.	b.t.	b.t.	b.t.	b.t.	b.t.	7,30E+06	6,57E+06	5,26E+06
P51572	B-cell receptor-associated protein 31	b.t.	b.t.	b.t.	b.t.	b.t.	b.t.	b.t.	b.t.	b.t.	b.t.	b.t.	b.t.	b.t.	b.t.	b.t.	3,90E+06	3,51E+06	2,81E+06
P51665	26S proteasome non-ATPase regulatory subunit 7	b.t.	b.t.	b.t.	b.t.	b.t.	b.t.	b.t.	b.t.	b.t.	b.t.	b.t.	b.t.	b.t.	b.t.	b.t.	4,60E+06	4,14E+06	3,31E+06
P51991	Heterogeneous nuclear ribonucleoprotein A3	b.t.	b.t.	b.t.	7,20E+06	6,60E+06	6,00E+06	b.t.	b.t.	b.t.	2,40E+06	3,60E+06	2,40E+06	1,35E+07	1,50E+07	1,43E+07	1,20E+07	1,08E+07	8,64E+06
P52209	6-phosphogluconate dehydrogenase, decarboxylating	2,29E+06	3,60E+06	9,80E+05	2,03E+07	2,10E+07	1,40E+07	4,75E+06	2,30E+06	3,60E+06	4,70E+06	1,50E+07	1,60E+07	6,57E+07	7,30E+07	6,94E+07	5,70E+07	4,90E+07	5,90E+07
P52272	Heterogeneous nuclear ribonucleoprotein M	b.t.	b.t.	b.t.	b.t.	b.t.	b.t.	b.t.	b.t.	b.t.	2,40E+06	3,60E+06	2,40E+06	b.t.	b.t.	b.t.	1,00E+07	9,00E+06	7,20E+06
P52789	Hexokinase-2	b.t.	b.t.	b.t.	2,92E+06	2,90E+06	2,10E+06	b.t.	b.t.	b.t.	b.t.	b.t.	b.t.	4,14E+06	4,60E+06	4,37E+06	7,52E+06	6,50E+06	6,10E+06
P52895	Aldo-keto reductase family 1 member C2	b.t.	b.t.	b.t.	b.t.	b.t.	b.t.	b.t.	b.t.	b.t.	1,20E+06	1,80E+06	1,20E+06	2,88E+07	3,20E+07	3,04E+07	2,20E+07	1,98E+07	1,58E+07
P53396	ATP-citrate synthase	1,18E+06	1,90E+06	4,60E+05	5,56E+06	5,80E+06	3,80E+06	1,46E+06	1,21E+06	9,70E+05	2,08E+06	8,60E+05	3,30E+06	1,26E+07	1,40E+07	1,33E+07	7,50E+06	1,50E+07	1,60E+07
P53621	Coatomer subunit alpha	b.t.	b.t.	b.t.	4,30E+06	4,30E+06	5,59E+06	b.t.	b.t.	b.t.	1,61E+06	9,20E+05	2,30E+06	3,96E+06	4,40E+06	4,18E+06	3,40E+06	7,20E+06	5,50E+06
P53992	Protein transport protein Sec24C	b.t.	b.t.	b.t.	1,32E+06	1,21E+06	1,10E+06	b.t.	b.t.	b.t.	b.t.	b.t.	b.t.	3,69E+06	4,10E+06	3,90E+06	2,52E+06	2,27E+06	2,10E+06
P54136	Arginine-tRNA ligase, cytoplasmic	b.t.	b.t.	b.t.	3,30E+06	3,30E+06	4,29E+06	b.t.	b.t.	b.t.	b.t.	b.t.	b.t.	8,19E+06	9,10E+06	8,65E+06	4,90E+06	6,90E+06	7,40E+06
P54727	UV excision repair protein RAD23 homolog B	b.t.	b.t.	b.t.	b.t.	b.t.	b.t.	b.t.	b.t.	b.t.	b.t.	b.t.	b.t.	6,57E+06	7,30E+06	6,94E+06	b.t.	b.t.	b.t.
P54886	Delta-1-pyrroline-5-carboxylate synthase	b.t.	b.t.	b.t.	b.t.	b.t.	b.t.	b.t.	b.t.	b.t.	b.t.	b.t.	b.t.	1,35E+07	1,50E+07	1,43E+07	7,60E+06	6,84E+06	1,10E+07
P55010	Eukaryotic translation initiation factor 5	b.t.	b.t.	b.t.	2,33E+06	2,70E+06	1,40E+06	b.t.	b.t.	b.t.	b.t.	b.t.	b.t.	1,26E+07	1,40E+07	1,33E+07	7,90E+06	7,11E+06	5,69E+06
P55060	Exportin-2	b.t.	b.t.	b.t.	4,20E+06	4,20E+06	5,46E+06	b.t.	b.t.	b.t.	b.t.	b.t.	b.t.	9,90E+06	1,10E+07	1,05E+07	2,90E+06	8,00E+06	6,30E+06
P55210	Caspase-7	b.t.	b.t.	b.t.	b.t.	b.t.	b.t.	b.t.	b.t.	b.t.	b.t.	b.t.	b.t.	b.t.	b.t.	b.t.	2,40E+06	2,16E+06	1,73E+06
P55786	Puromycin-sensitive aminopeptidase	b.t.	b.t.	b.t.	6,49E+06	9,20E+06	2,70E+06	b.t.	b.t.	b.t.	1,46E+06	8,10E+05	2,10E+06	1,17E+07	1,30E+07	1,24E+07	5,70E+06	9,70E+06	1,10E+07
P56192	Methionine-tRNA ligase, cytoplasmic	b.t.	b.t.	b.t.	b.t.	b.t.	b.t.	b.t.	b.t.	b.t.	b.t.	b.t.	b.t.	1,26E+06	1,40E+06	1,33E+06	1,40E+06	1,26E+06	5,30E+06
P56537	Eukaryotic translation initiation factor 6	b.t.	b.t.	b.t.	3,99E+06	4,20E+06	2,70E+06	b.t.	b.t.	b.t.	b.t.	b.t.	b.t.	3,60E+06	4,00E+06	3,80E+06	1,60E+07	1,44E+07	1,15E+07
P57053	Histone H2B type F-S	b.t.	b.t.	b.t.	b.t.	b.t.	b.t.	b.t.	b.t.	b.t.	2,80E+06	4,20E+06	2,80E+06	1,08E+07	1,20E+07	1,14E+07	b.t.	b.t.	b.t.
P59998	Actin-related protein 2/3 complex subunit 4	b.t.	b.t.	b.t.	b.t.	b.t.	b.t.	b.t.	b.t.	b.t.	6,20E+06	9,30E+06	6,20E+06	b.t.	b.t.	b.t.	1,60E+07	1,44E+07	1,15E+07
P60842	Eukaryotic initiation factor 4A-I	3,20E+05	3,20E+05	4,80E+05	5,26E+06	5,90E+06	3,30E+06	1,95E+06	1,63E+06	1,30E+06	5,05E+06	3,10E+06	7,00E+06	2,61E+07	2,90E+07	2,76E+07	1,90E+07	1,70E+07	3,80E+07
P60866	40S ribosomal protein S20	b.t.	b.t.	b.t.	b.t.	b.t.	b.t.	b.t.	b.t.	b.t.	6,10E+06	9,15E+06	6,10E+06	1,08E+07	1,20E+07	1,14E+07	b.t.	b.t.	b.t.
P60891	Ribose-phosphate pyrophosphokinase 1	b.t.	b.t.	b.t.	b.t.	b.t.	b.t.	1,65E+06	1,38E+06	1,10E+06	3,11E+06	7,20E+05	5,50E+06	1,62E+07	1,80E+07	1,71E+07	1,10E+07	9,90E+06	7,92E+06
P60900	Proteasome subunit alpha type-6	b.t.	b.t.	b.t.	1,56E+07	1,43E+07	1,30E+07	1,50E+06	1,25E+06	1,00E+06	1,60E+07	2,40E+07	1,60E+07	2,25E+07	2,50E+07	2,38E+07	1,30E+07	5,30E+05	4,24E+05
P61077	Ubiquitin-conjugating enzyme E2 D3	b.t.	b.t.	b.t.	2,04E+06	1,87E+06	1,70E+06	b.t.	b.t.	b.t.	1,70E+06	2,55E+06	1,70E+06	9,90E+06	1,10E+07	1,05E+07	9,30E+06	8,37E+06	6,70E+06
P61204	ADP-ribosylation factor 3	b.t.	b.t.	b.t.	6,60E+06	6,05E+06	5,50E+06	1,80E+06	1,50E+06	1,20E+06	2,10E+07	3,15E+07	2,10E+07	2,52E+07	2,80E+07	2,66E+07	4,70E+06	4,23E+06	3,38E+06
P61604	10 kDa heat shock protein, mitochondrial	5,60E+06	8,40E+06	5,60E+06	b.t.	b.t.	b.t.	6,30E+06	5,25E+06	4,20E+06	1,67E+07	4,70E+05	3,30E+07	1,08E+08	1,20E+08	1,14E+08	1,50E+08	1,35E+08	2,00E+06
P61970	Nuclear transport factor 2	b.t.	b.t.	b.t.	b.t.	b.t.	b.t.	b.t.	b.t.	b.t.	1,30E+07	1,95E+07	1,30E+07	1,71E+07	1,90E+07	1,81E+07	2,10E+07	1,10E+06	8,80E+05
P61978	Heterogeneous nuclear ribonucleoprotein K	1,70E+06	1,70E+06	2,55E+06	2,16E+07	1,98E+07	1,80E+07	4,85E+06	1,10E+06	4,30E+06	1,00E+06	1,70E+07	9,30E+06	4,05E+07	4,50E+07	4,28E+07	7,90E+07	1,40E+06	1,30E+06
P61981	14-3-3 protein gamma	b.t.	b.t.	b.t.	7,86E+06	8,30E+06	5,30E+06	1,40E+06	1,40E+06	1,68E+06	2,10E+06	2,40E+06	1,80E+06	2,25E+07	2,50E+07	2,38E+07	3,20E+07	2,30E+06	5,10E+06
P62081	40S ribosomal protein S7	b.t.	b.t.	b.t.	b.t.	b.t.	b.t.	b.t.	b.t.	b.t.	5,90E+06	8,85E+06	5,90E+06	b.t.	b.t.	b.t.	8,20E+06	7,38E+06	1,20E+06
P62136	Serine/threonine-protein phosphatase PP1-alpha catalytic subunit	b.t.	b.t.	b.t.	4,83E+06	2,80E+06	4,90E+06	b.t.	b.t.	b.t.	1,50E+06	1,50E+06	2,25E+06	1,80E+07	2,00E+07	1,90E+07	5,60E+06	5,04E+06	4,03E+06
P62195	26S protease regulatory subunit 8	b.t.	b.t.	b.t.	1,32E+06	1,21E+06	1,10E+06	b.t.	b.t.	b.t.	b.t.	b.t.	b.t.	7,20E+06	8,00E+06	7,60E+06	1,90E+06	5,30E+06	5,40E+06
P62244	40S ribosomal protein S15a	b.t.	b.t.	b.t.	b.t.	b.t.	b.t.	b.t.	b.t.	b.t.	3,10E+07	4,65E+07	3,10E+07	2,25E+07	2,50E+07	2,38E+07	2,00E+07	6,50E+06	5,20E+06
P62258	14-3-3 protein epsilon	3,65E+06	5,10E+06	2,20E+06	1,07E+07	9,90E+06	8,20E+06	1,06E+07	6,10E+06	7,50E+06	6,00E+06	4,90E+06	7,10E+06	3,96E+07	4,40E+07	4,18E+07	8,50E+07	2,70E+06	2,20E+06
P62263	40S ribosomal protein S14	b.t.	b.t.	b.t.	b.t.	b.t.	b.t.	b.t.	b.t.	b.t.	b.t.	b.t.	b.t.	4,77E+06	5,30E+06	5,04E+06	b.t.	b.t.	b.t.
P62269	40S ribosomal protein S18	b.t.	b.t.	b.t.	b.t.	b.t.	b.t.	b.t.	b.t.	b.t.	8,60E+06	1,29E+07	8,60E+06	2,34E+07	2,60E+07	2,47E+07	2,80E+07	2,52E+07	2,40E+06
P62277	40S ribosomal protein S13	3,40E+05	3,40E+05	5,10E+05	2,74E+07	2,40E+07	2,20E+07	3,15E+06	2,63E+06	2,10E+06	2,70E+07	4,05E+07	2,70E+07	5,58E+07	6,20E+07	5,89E+07	3,60E+07	7,40E+06	5,92E+06
P62314	Small nuclear ribonucleoprotein Sm D1	2,90E+06	2,90E+06	4,35E+06	1,14E+07	8,70E+06	1,00E+07	7,55E+06	1,10E+06	7,00E+06	6,10E+06	9,15E+06	6,10E+06	1,44E+07	1,60E+07	1,52E+07	3,66E+06	2,70E+06	3,30E+06
P62424	60S ribosomal protein L7a	b.t.	b.t.	b.t.	b.t.	b.t.	b.t.	b.t.	b.t.	b.t.	9,80E+06	1,47E+07	9,80E+06	1,08E+07	1,20E+07	1,14E+07	2,00E+07	1,80E+07	1,44E+07
P62495	Eukaryotic peptide chain release factor subunit 1	b.t.	b.t.	b.t.	b.t.	b.t.	b.t.	b.t.	b.t.	b.t.	b.t.	b.t.	b.t.	5,22E+06	5,80E+06	5,51E+06	8,10E+06	7,29E+06	5,83E+06
P62701	40S ribosomal protein S4	b.t.	b.t.	b.t.	3,12E+06	2,86E+06	2,60E+06	b.t.	b.t.	b.t.	2,95E+06	2,50E+06	3,40E+06	2,52E+07	2,80E+07	2,66E+07	3,90E+07	3,51E+07	2,81E+07
P62807	Histone H2B type 1-C/E/F/G/I	b.t.	b.t.	b.t.	b.t.	b.t.	b.t.	b.t.	b.t.	b.t.	2,80E+06	4,20E+06	2,80E+06	1,08E+07	1,20E+07	1,14E+07	b.t.	b.t.	b.t.
P62834	Ras-related protein Rap-1A	b.t.	b.t.	b.t.	b.t.	b.t.	b.t.	b.t.	b.t.	b.t.	b.t.	b.t.	b.t.	5,67E+06	6,30E+06	5,99E+06	3,00E+06	2,70E+06	2,16E+06
P62847	40S ribosomal protein S24	b.t.	b.t.	b.t.	b.t.	b.t.	b.t.	1,95E+06	1,63E+06	1,30E+06	b.t.	b.t.	b.t.	1,44E+07	1,60E+07	1,52E+07	7,80E+06	7,02E+06	1,20E+06
P62851	40S ribosomal protein S25	b.t.	b.t.	b.t.	b.t.	b.t.	b.t.	b.t.	b.t.	b.t.	2,20E+07	3,30E+07	2,20E+07	1,89E+07	2,10E+07	2,00E+07	3,10E+07	2,79E+07	2,23E+07
P62854	40S ribosomal protein S26	b.t.	b.t.	b.t.	b.t.	b.t.	b.t.	b.t.	b.t.	b.t.	6,50E+06	9,75E+06	6,50E+06	b.t.	b.t.	b.t.	8,00E+06	7,20E+06	5,76E+06
P62888	60S ribosomal protein L30	b.t.	b.t.	b.t.	8,52E+07	7,81E+07	7,10E+07	1,60E+07	2,40E+07	1,60E+07	b.t.	b.t.	b.t.	1,08E+06	1,20E+06	1,14E+06	1,20E+06	1,08E+06	8,64E+05
P62906	60S ribosomal protein L10a	b.t.	b.t.	b.t.	b.t.	b.t.	b.t.	b.t.	b.t.	b.t.	6,10E+06	9,15E+06	6,10E+06	8,55E+06	9,50E+06	9,03E+06	1,70E+07	1,53E+07	1,22E+07
P62913	60S ribosomal protein L11	b.t.	b.t.	b.t.	b.t.	b.t.	b.t.	b.t.	b.t.	b.t.	2,20E+06	3,30E+06	2,20E+06	3,60E+06	4,00E+06	3,80E+06	2,10E+06	1,89E+06	1,51E+06
P62917	60S ribosomal protein L8	b.t.	b.t.	b.t.	b.t.	b.t.	b.t.	b.t.	b.t.	b.t.	2,00E+06	3,00E+06	2,00E+06	4,77E+06	5,30E+06	5,04E+06	1,00E+07	9,00E+06	7,20E+06
P62937	Peptidyl-prolyl cis-trans isomerase A	1,30E+06	1,30E+06	1,95E+06	2,22E+07	1,50E+07	2,10E+07	3,02E+07	8,30E+06	2,60E+07	7,20E+06	3,90E+06	1,20E+07	1,98E+08	2,20E+08	2,09E+08	3,40E+08	3,06E+08	6,00E+06
P62979	Ubiquitin-40S ribosomal protein S27a	1,23E+06	7,60E+05	1,70E+06	2,80E+06	6,60E+06	2,80E+07	1,10E+07	9,13E+06	7,30E+06	3,00E+06	2,60E+06	5,90E+07	1,08E+08	1,20E+08	1,14E+08	1,30E+08	4,20E+07	1,80E+07
P62995	Transformer-2 protein homolog beta	b.t.	b.t.	b.t.	b.t.	b.t.	b.t.	b.t.	b.t.	b.t.	b.t.	b.t.	b.t.	b.t.	b.t.	b.t.	9,00E+06	8,10E+06	6,48E+06
P63104	14-3-3 protein zeta/delta	2,60E+06	2,60E+06	3,90E+06	1,73E+07	2,40E+07	7,60E+06	5,80E+06	1,60E+06	5,00E+06	8,90E+06	1,40E+07	3,80E+06	4,77E+07	5,30E+07	5,04E+07	5,70E+07	5,13E+07	1,60E+06
P63162	Small nuclear ribonucleoprotein-associated protein N	b.t.	b.t.	b.t.	6,60E+06	6,05E+06	5,50E+06	b.t.	b.t.	b.t.	7,90E+06	1,19E+07	7,90E+06	1,35E+07	1,50E+07	1,43E+07	2,50E+07	1,20E+06	1,20E+06
P63261	Actin, cytoplasmic 2	6,20E+06	4,80E+06	7,60E+06	8,20E+06	2,60E+08	3,40E+07	1,60E+07	1,20E+07	1,00E+07	5,80E+07	1,30E+08	2,20E+07	7,20E+08	8,00E+08	7,60E+08	3,80E+08	6,20E+06	6,00E+08
P68104	Elongation factor 1-alpha 1	b.t.	b.t.	b.t.	1,15E+07	1,20E+07	7,90E+06	7,35E+05	6,13E+05	4,90E+05	3,90E+06	1,40E+06	1,10E+07	6,75E+07	7,50E+07	7,13E+07	6,50E+07	2,00E+07	2,50E+07
P68363	Tubulin alpha-1B	b.t.	b.t.	b.t.	3,33E+06	2,60E+06	2,90E+06	4,50E+06	3,75E+06	3,00E+06	7,70E+06	4,40E+06	1,10E+07	5,31E+07	5,90E+07	5,61E+07	1,10E+07	1,90E+05	1,40E+07
P68366	Tubulin alpha-4A	b.t.	b.t.	b.t.	b.t.	b.t.	b.t.	b.t.	b.t.	b.t.	3,10E+06	3,10E+06	4,65E+06	b.t.	b.t.	b.t.	5,20E+06	4,68E+06	3,74E+06
P68371	Tubulin beta-4B	b.t.	b.t.	b.t.	7,80E+05	7,15E+05	6,50E+05	b.t.	b.t.	b.t.	2,20E+06	1,10E+06	3,30E+06	2,88E+07	3,20E+07	3,04E+07	2,88E+06	2,59E+06	2,40E+06

P78371	T-complex protein 1 subunit beta	b.t.	b.t.	b.t.	5,14E+06	2,30E+06	5,70E+06	1,32E+06	1,10E+06	8,80E+05	8,50E+06	1,28E+07	8,50E+06	2,25E+07	2,50E+07	2,38E+07	3,20E+07	2,88E+07	2,30E+07
P78527	DNA-dependent protein kinase catalytic subunit	b.t.	b.t.	b.t.	3,82E+06	6,10E+06	1,10E+06	b.t.	b.t.	b.t.	b.t.	b.t.	b.t.	1,35E+07	1,50E+07	1,43E+07	2,30E+06	6,70E+06	6,20E+06
P84077	ADP-ribosylation factor 1	b.t.	b.t.	b.t.	6,60E+06	6,05E+06	5,50E+06	1,80E+06	1,50E+06	1,20E+06	2,10E+07	3,15E+07	2,10E+07	2,52E+07	2,80E+07	2,66E+07	4,70E+06	4,23E+06	3,38E+06
P84098	60S ribosomal protein L19	b.t.	b.t.	b.t.	b.t.	b.t.	b.t.	b.t.	b.t.	b.t.	b.t.	b.t.	b.t.	7,11E+06	7,90E+06	7,51E+06	4,90E+06	4,41E+06	3,53E+06
P99999	Cytochrome c	b.t.	b.t.	b.t.	2,24E+06	2,80E+06	1,20E+06	1,20E+06	1,20E+06	1,44E+06	7,25E+05	2,50E+05	1,20E+06	2,34E+07	2,60E+07	2,47E+07	2,80E+07	2,52E+07	6,20E+05
Q00610	Clathrin heavy chain 1	1,16E+06	1,50E+06	8,20E+05	1,59E+07	2,60E+07	4,20E+06	3,15E+06	2,63E+06	2,10E+06	7,40E+05	7,40E+05	1,11E+06	1,35E+07	1,50E+07	1,43E+07	8,50E+06	1,40E+07	2,50E+07
Q00839	Heterogeneous nuclear ribonucleoprotein U	b.t.	b.t.	b.t.	8,70E+06	2,00E+06	1,10E+07	8,70E+06	7,25E+06	5,80E+06	3,40E+06	8,90E+06	1,10E+07	4,32E+07	4,80E+07	4,56E+07	2,70E+07	4,10E+06	6,30E+06
Q02878	60S ribosomal protein L6	3,60E+05	3,60E+05	5,40E+05	3,98E+08	3,20E+08	3,40E+08	6,15E+08	5,13E+08	4,10E+08	6,80E+05	1,02E+05	6,80E+05	1,53E+07	1,70E+07	1,62E+07	3,00E+07	2,10E+06	9,90E+06
Q04446	1.4-alpha-glucan-branching enzyme	b.t.	b.t.	b.t.	b.t.	b.t.	b.t.	b.t.	b.t.	b.t.	b.t.	b.t.	b.t.	b.t.	b.t.	b.t.	3,40E+06	3,40E+06	2,72E+06
Q04695	Keratin. type I cytoskeletal 17	b.t.	b.t.	b.t.	4,30E+06	5,10E+06	2,50E+06	b.t.	b.t.	b.t.	b.t.	b.t.	b.t.	b.t.	b.t.	b.t.	3,00E+06	2,70E+06	5,70E+06
Q04837	Single-stranded DNA-binding protein, mitochondrial	b.t.	b.t.	b.t.	6,60E+06	6,05E+06	5,50E+06	b.t.	b.t.	b.t.	6,30E+06	9,45E+06	6,30E+06	1,80E+07	2,00E+07	1,90E+07	1,70E+07	1,53E+07	1,22E+07
Q05639	Elongation factor 1-alpha 2	6,30E+06	6,30E+06	9,45E+06	2,76E+07	4,90E+07	4,40E+06	b.t.	b.t.	b.t.	2,60E+06	2,60E+06	3,90E+06	1,71E+07	1,90E+07	1,81E+07	1,80E+07	1,00E+07	2,00E+07
Q05CW6	PSMD1 protein	b.t.	b.t.	b.t.	5,50E+06	5,50E+06	7,15E+06	b.t.	b.t.	b.t.	b.t.	b.t.	b.t.	b.t.	b.t.	b.t.	4,60E+06	9,30E+06	7,00E+06
Q05D08	PA2G4 protein A	b.t.	b.t.	b.t.	b.t.	b.t.	b.t.	b.t.	b.t.	b.t.	1,50E+06	1,50E+06	2,25E+06	1,17E+07	1,30E+07	1,24E+07	1,00E+07	9,00E+06	1,10E+07
Q05D43	YBX1 protein	b.t.	b.t.	b.t.	b.t.	b.t.	b.t.	b.t.	b.t.	b.t.	5,60E+06	8,40E+06	5,60E+06	b.t.	b.t.	b.t.	1,20E+07	1,08E+07	8,64E+06
Q06210	Glutamine-fructose-6-phosphate aminotransferase [isomerizing] 1	b.t.	b.t.	b.t.	b.t.	b.t.	b.t.	b.t.	b.t.	b.t.	b.t.	b.t.	b.t.	5,67E+06	6,30E+06	5,99E+06	8,10E+06	7,29E+06	5,83E+06
Q06830	Peroxiredoxin-1	b.t.	b.t.	b.t.	6,47E+06	3,00E+06	7,10E+06	4,40E+06	1,40E+06	3,70E+06	2,60E+06	6,80E+05	4,90E+07	1,53E+08	1,70E+08	1,62E+08	1,20E+08	1,80E+07	6,20E+06
Q07020	60S ribosomal protein L18	b.t.	b.t.	b.t.	b.t.	b.t.	b.t.	b.t.	b.t.	b.t.	1,10E+07	1,65E+07	1,10E+07	1,08E+07	1,20E+07	1,14E+07	1,30E+07	1,17E+07	9,36E+06
Q07021	Complement component 1 Q subcomponent-binding protein, mitocho	b.t.	b.t.	b.t.	3,96E+06	3,63E+06	3,30E+06	b.t.	b.t.	b.t.	b.t.	b.t.	b.t.	2,97E+07	3,30E+07	3,14E+07	7,70E+06	6,93E+06	5,54E+06
Q08170	Serine/arginine-rich splicing factor 4	b.t.	b.t.	b.t.	b.t.	b.t.	b.t.	b.t.	b.t.	b.t.	b.t.	b.t.	b.t.	1,08E+07	1,20E+07	1,14E+07	5,40E+06	4,86E+06	3,89E+06
Q08211	ATP-dependent RNA helicase A	1,40E+06	1,40E+06	2,10E+06	6,87E+06	8,70E+06	3,60E+06	2,40E+06	2,00E+06	1,60E+06	3,00E+06	3,00E+06	4,50E+06	1,17E+07	1,30E+07	1,24E+07	1,00E+07	1,50E+07	3,00E+07
Q08AJ6	ANP32A protein	b.t.	b.t.	b.t.	6,72E+06	6,16E+06	5,60E+06	2,85E+06	2,38E+06	1,90E+06	4,10E+06	6,15E+06	4,10E+06	4,50E+06	5,00E+06	4,75E+06	2,50E+07	2,25E+07	1,80E+07
Q08AJ9	Histone H2A	b.t.	b.t.	b.t.	b.t.	b.t.	b.t.	b.t.	b.t.	b.t.	5,50E+05	5,50E+05	8,25E+05	b.t.	b.t.	b.t.	2,80E+07	2,52E+07	2,02E+07
Q08ES8	Cell growth-inhibiting protein 34	b.t.	b.t.	b.t.	b.t.	b.t.	b.t.	b.t.	b.t.	b.t.	2,20E+06	3,30E+06	2,20E+06	3,60E+06	4,00E+06	3,80E+06	2,10E+06	1,89E+06	1,51E+06
Q08J23	tRNA (cytosine(34)-C(5))-methyltransferase	b.t.	b.t.	b.t.	4,68E+06	4,29E+06	3,90E+06	b.t.	b.t.	b.t.	b.t.	b.t.	b.t.	b.t.	b.t.	b.t.	5,52E+06	4,97E+06	4,60E+06
Q0D2M2	HIST1H2BC protein	b.t.	b.t.	b.t.	b.t.	b.t.	b.t.	b.t.	b.t.	b.t.	2,80E+06	4,20E+06	2,80E+06	1,08E+07	1,20E+07	1,14E+07	b.t.	b.t.	b.t.
Q0IJ56	ST13 protein	b.t.	b.t.	b.t.	b.t.	b.t.	b.t.	b.t.	b.t.	b.t.	1,20E+06	1,20E+06	1,80E+06	5,49E+06	6,10E+06	5,80E+06	4,60E+06	4,14E+06	3,31E+06
Q0VGD6	HNRPR protein	b.t.	b.t.	b.t.	4,92E+06	4,51E+06	4,10E+06	3,15E+06	2,63E+06	2,10E+06	2,00E+06	1,60E+06	2,40E+06	7,83E+06	8,70E+06	8,27E+06	4,60E+06	4,14E+06	6,20E+06
Q12771	P37 AUF1	b.t.	b.t.	b.t.	6,96E+06	6,38E+06	5,80E+06	5,10E+06	4,25E+06	3,40E+06	3,45E+06	1,90E+06	5,00E+06	2,79E+07	3,10E+07	2,95E+07	1,70E+07	1,53E+07	1,80E+06
Q12797	Aspartyl/asparaginyl beta-hydroxylase	b.t.	b.t.	b.t.	1,06E+06	9,68E+05	8,80E+05	b.t.	b.t.	b.t.	b.t.	b.t.	b.t.	4,23E+06	4,70E+06	4,47E+06	4,71E+06	5,50E+06	2,80E+06
Q12906	Interleukin enhancer-binding factor 3	b.t.	b.t.	b.t.	1,32E+06	1,21E+06	1,10E+06	b.t.	b.t.	b.t.	6,80E+05	6,80E+05	1,02E+06	1,08E+07	1,20E+07	1,14E+07	6,40E+06	7,40E+06	6,30E+06
Q12931	Heat shock protein 75 kDa, mitochondrial	b.t.	b.t.	b.t.	3,60E+06	3,20E+06	1,70E+07	4,15E+06	3,10E+06	2,60E+06	7,00E+06	9,80E+06	6,10E+06	1,62E+07	1,80E+07	1,71E+07	5,80E+07	6,80E+06	2,60E+07
Q13011	Delta(3,5)-Delta(2,4)-dienoyl-CoA isomerase, mitochondrial	3,70E+05	3,70E+05	5,55E+05	b.t.	b.t.	b.t.	2,70E+06	2,25E+06	1,80E+06	b.t.	b.t.	b.t.	2,97E+06	3,30E+06	3,14E+06	2,00E+07	1,80E+07	6,40E+05
Q13200	26S proteasome non-ATPase regulatory subunit 2	b.t.	b.t.	b.t.	2,77E+06	3,30E+06	1,60E+06	b.t.	b.t.	b.t.	b.t.	b.t.	b.t.	4,95E+06	5,50E+06	5,23E+06	4,10E+06	3,69E+06	6,30E+06
Q13228	Selenium-binding protein 1	b.t.	b.t.	b.t.	3,20E+06	3,20E+06	4,16E+06	b.t.	b.t.	b.t.	b.t.	b.t.	b.t.	b.t.	b.t.	b.t.	b.t.	b.t.	b.t.
Q13247	Serine/arginine-rich splicing factor 6	b.t.	b.t.	b.t.	b.t.	b.t.	b.t.	b.t.	b.t.	b.t.	b.t.	b.t.	b.t.	1,08E+07	1,20E+07	1,14E+07	5,40E+06	4,86E+06	3,89E+06
Q13310	Polyadenylate-binding protein 4	b.t.	b.t.	b.t.	2,40E+06	2,20E+06	2,00E+06	b.t.	b.t.	b.t.	5,20E+05	5,20E+05	7,80E+05	b.t.	b.t.	b.t.	1,70E+06	1,53E+06	1,22E+06
Q13509	Tubulin beta-3	b.t.	b.t.	b.t.	b.t.	b.t.	b.t.	b.t.	b.t.	b.t.	5,50E+05	5,50E+05	8,25E+05	9,90E+06	1,10E+07	1,05E+07	1,60E+06	1,44E+06	1,15E+06
Q13813	Spectrin α chain	6,20E+06	4,80E+06	7,60E+06	8,20E+06	2,60E+08	3,40E+07	1,60E+07	1,20E+07	1,00E+07	5,80E+07	1,30E+08	2,20E+07	7,20E+08	8,00E+08	7,60E+08	3,80E+08	6,20E+06	6,00E+08
Q13838	Spliceosome RNA helicase DDX39B	b.t.	b.t.	b.t.	1,92E+06	1,76E+06	1,60E+06	3,30E+06	2,75E+06	2,20E+06	b.t.	b.t.	b.t.	b.t.	b.t.	b.t.	3,90E+06	3,51E+06	7,50E+06
Q13885	Tubulin beta-2A	b.t.	b.t.	b.t.	7,80E+05	7,15E+05	6,50E+05	b.t.	b.t.	b.t.	2,20E+06	1,10E+06	3,30E+06	2,88E+07	3,20E+07	3,04E+07	2,88E+06	2,59E+06	2,40E+06
Q14315	Filamin-C	b.t.	b.t.	b.t.	1,18E+06	1,08E+06	9,80E+05	b.t.	b.t.	b.t.	b.t.	b.t.	b.t.	b.t.	b.t.	b.t.	1,70E+07	3,20E+06	2,56E+06
Q14498	RNA-binding protein 39	b.t.	b.t.	b.t.	b.t.	b.t.	b.t.	b.t.	b.t.	b.t.	b.t.	b.t.	b.t.	b.t.	b.t.	b.t.	6,50E+06	5,85E+06	4,68E+06
Q14914	Prostaglandin reductase 1	6,60E+05	9,90E+05	6,60E+05	8,28E+06	7,59E+06	6,90E+06	3,90E+06	3,25E+06	2,60E+06	8,60E+06	1,29E+07	8,60E+06	1,98E+07	2,20E+07	2,09E+07	1,80E+07	1,62E+07	1,20E+06
Q14974	Importin subunit beta-1	2,20E+06	2,20E+06	3,30E+06	1,34E+07	2,00E+07	4,80E+06	b.t.	b.t.	b.t.	1,90E+06	1,70E+06	2,10E+06	2,97E+07	3,30E+07	3,14E+07	1,00E+07	1,90E+07	2,30E+07
Q15008	26S proteasome non-ATPase regulatory subunit 6	b.t.	b.t.	b.t.	1,30E+07	1,30E+07	1,69E+07	b.t.	b.t.	b.t.	b.t.	b.t.	b.t.	4,86E+06	5,40E+06	5,13E+06	3,10E+06	5,80E+07	3,60E+06
Q15029	116 kDa U5 small nuclear ribonucleoprotein component	b.t.	b.t.	b.t.	2,30E+06	2,30E+06	2,99E+06	b.t.	b.t.	b.t.	b.t.	b.t.	b.t.	b.t.	b.t.	b.t.	2,20E+06	1,98E+06	1,58E+06
Q15046	Lysine-tRNA ligase	b.t.	b.t.	b.t.	b.t.	b.t.	b.t.	b.t.	b.t.	b.t.	b.t.	b.t.	b.t.	b.t.	b.t.	b.t.	1,70E+06	1,70E+06	1,36E+06
Q15056	Eukaryotic translation initiation factor 4H	1,80E+06	1,80E+06	2,70E+06	3,60E+06	3,30E+06	3,00E+06	b.t.	b.t.	b.t.	b.t.	b.t.	b.t.	b.t.	b.t.	b.t.	1,20E+07	1,08E+07	1,50E+06
Q15084	Protein disulfide-isomerase A6	7,25E+05	3,50E+05	1,10E+06	1,44E+07	9,10E+06	1,40E+07	9,15E+06	1,90E+06	8,20E+06	2,80E+06	2,80E+06	3,90E+06	4,14E+07	4,60E+07	4,37E+07	4,60E+07	3,20E+06	7,70E+06
Q15181	Inorganic pyrophosphatase 2, mitochondrial	b.t.	b.t.	b.t.	b.t.	b.t.	b.t.	b.t.	b.t.	b.t.	b.t.	b.t.	b.t.	3,15E+06	3,50E+06	3,33E+06	b.t.	b.t.	b.t.
Q15182	Small nuclear ribonucleoprotein-associated protein A	b.t.	b.t.	b.t.	6,60E+06	6,05E+06	5,50E+06	b.t.	b.t.	b.t.	7,90E+06	1,19E+07	7,90E+06	1,35E+07	1,50E+07	1,43E+07	2,50E+07	1,20E+06	1,20E+06
Q15366	Poly(rC)-binding protein 2b	b.t.	b.t.	b.t.	b.t.	b.t.	b.t.	2,10E+06	1,75E+06	1,40E+06	3,90E+05	3,90E+05	5,85E+05	6,48E+06	7,20E+06	6,84E+06	1,10E+06	1,10E+06	8,80E+05
Q15393	Splicing factor 3B subunit 3	b.t.	b.t.	b.t.	3,50E+06	3,50E+06	4,55E+06	b.t.	b.t.	b.t.	b.t.	b.t.	b.t.	b.t.	b.t.	b.t.	8,03E+06	9,90E+06	4,40E+06
Q15717	ELAV-like protein 1	b.t.	b.t.	b.t.	1,80E+06	1,65E+06	1,50E+06	b.t.	b.t.	b.t.	b.t.	b.t.	b.t.	b.t.	b.t.	b.t.	b.t.	b.t.	b.t.
Q15907	Ras-related protein Rab-11B	b.t.	b.t.	b.t.	9,72E+06	8,91E+06	8,10E+06	b.t.	b.t.	b.t.	b.t.	b.t.	b.t.	b.t.	b.t.	b.t.	2,40E+05	2,40E+05	1,92E+05
Q16401	26S proteasome non-ATPase regulatory subunit 5	b.t.	b.t.	b.t.	2,76E+06	2,53E+06	2,30E+06	b.t.	b.t.	b.t.	b.t.	b.t.	b.t.	7,20E+06	8,00E+06	7,60E+06	7,93E+06	8,30E+06	5,40E+06
Q16531	DNA damage-binding protein 1	b.t.	b.t.	b.t.	3,20E+06	3,20E+06	4,16E+06	b.t.	b.t.	b.t.	b.t.	b.t.	b.t.	b.t.	b.t.	b.t.	1,80E+06	1,62E+06	1,50E+06
Q16555	Dihydropyrimidinase-related protein 2	b.t.	b.t.	b.t.	b.t.	b.t.	b.t.	b.t.	b.t.	b.t.	2,35E+06	1,10E+06	3,60E+06	1,17E+07	1,30E+07	1,24E+07	5,30E+06	4,77E+06	7,70E+06
Q16629	Serine/arginine-rich splicing factor 7	b.t.	b.t.	b.t.	b.t.	b.t.	b.t.	b.t.	b.t.	b.t.	b.t.	b.t.	b.t.	b.t.	b.t.	b.t.	7,30E+06	6,57E+06	5,26E+06
Q16719	Kynureninase	b.t.	b.t.	b.t.	2,04E+06	1,87E+06	1,70E+06	b.t.	b.t.	b.t.	b.t.	b.t.	b.t.	4,32E+06	4,80E+06	4,56E+06	3,90E+06	3,51E+06	2,81E+06
Q16778	Histone H2B type 2-E	3,40E+06	5,10E+06	3,40E+06	b.t.	b.t.	b.t.	4,50E+06	3,75E+06	3,00E+06	1,30E+07	1,95E+07	1,30E+07	b.t.	b.t.	b.t.	1,90E+07	1,71E+07	1,37E+07
Q16836	Hydroxyacyl-coenzyme A dehydrogenase, mitochondrial	b.t.	b.t.	b.t.	3,54E+06	6,40E+05	4,60E+06	b.t.	b.t.	b.t.	b.t.	b.t.	b.t.	b.t.	b.t.	b.t.	3,00E+07	2,70E+07	2,16E+07
Q16851	UTP-glucose-1-phosphate uridylyltransferase	b.t.	b.t.	b.t.	4,00E+06	4,00E+06	5,20E+06	b.t.	b.t.	b.t.	b.t.	b.t.	b.t.	8,73E+06	9,70E+06	9,22E+06	4,20E+06	3,78E+06	5,10E+06

Q16881	Thioredoxin reductase 1a	1,70E+06	1,70E+06	2,55E+06	3,20E+07	5,30E+07	7,80E+06	1,65E+06	1,38E+06	1,10E+06	2,60E+06	3,70E+06	8,00E+06	7,29E+07	8,10E+07	7,70E+07	2,60E+07	3,00E+07	4,40E+07
Q1XBU6	Aging-associated protein 14b	b.t.	b.t.	b.t.	b.t.	b.t.	b.t.	b.t.	b.t.	b.t.	1,20E+06	1,20E+06	1,80E+06	5,49E+06	6,10E+06	5,80E+06	4,60E+06	4,14E+06	3,31E+06
Q24JU4	Eukaryotic translation initiation factor 3 subunit A	b.t.	b.t.	b.t.	1,10E+06	1,01E+06	9,20E+05	b.t.	b.t.	b.t.	b.t.	b.t.	b.t.	b.t.	b.t.	b.t.	4,30E+06	4,90E+06	3,92E+06
Q2M1K9	Zinc finger protein 423	1,18E+06	1,90E+06	4,60E+05	5,56E+06	5,80E+06	3,80E+06	1,46E+06	1,21E+06	9,70E+05	2,08E+06	8,60E+05	3,30E+06	1,26E+07	1,40E+07	1,33E+07	7,50E+06	1,50E+07	1,60E+07
Q2NKY5	TUBB6 protein	b.t.	b.t.	b.t.	b.t.	b.t.	b.t.	b.t.	b.t.	b.t.	5,50E+05	5,50E+05	8,25E+05	9,90E+06	1,10E+07	1,05E+07	1,60E+06	1,44E+06	1,15E+06
Q2TNB3	Cell migration-inducing protein 22	b.t.	b.t.	b.t.	6,84E+05	6,27E+05	5,70E+05	b.t.	b.t.	b.t.	2,60E+06	1,70E+06	3,50E+06	5,22E+06	5,80E+06	5,51E+06	9,30E+06	8,37E+06	6,70E+06
Q2XPP3	Type II 3a-hydroxysteroid dehydrogenase	b.t.	b.t.	b.t.	6,96E+06	6,38E+06	5,80E+06	2,25E+06	1,30E+06	1,60E+06	3,10E+06	4,65E+06	3,10E+06	2,16E+07	2,40E+07	2,28E+07	3,10E+07	2,79E+07	1,40E+06
Q32Q12	Nucleoside diphosphate kinase	b.t.	b.t.	b.t.	1,44E+07	1,32E+07	1,20E+07	4,05E+06	3,38E+06	2,70E+06	2,20E+06	3,30E+06	2,20E+06	2,07E+07	2,30E+07	2,19E+07	1,20E+07	1,08E+07	5,20E+05
Q32Q75	Eukaryotic translation initiation factor 4E	b.t.	b.t.	b.t.	b.t.	b.t.	b.t.	b.t.	b.t.	b.t.	b.t.	b.t.	b.t.	4,23E+06	4,70E+06	4,47E+06	1,20E+07	1,08E+07	8,64E+06
Q3B7A3	SEPT7 protein	b.t.	b.t.	b.t.	b.t.	b.t.	b.t.	b.t.	b.t.	b.t.	b.t.	b.t.	b.t.	9,00E+06	1,00E+07	9,50E+06	b.t.	b.t.	b.t.
Q3B7A7	Phosphoribosylglycinamide formyltransferase/phosphoribosylglycinar	b.t.	b.t.	b.t.	2,80E+06	2,80E+06	3,64E+06	b.t.	b.t.	b.t.	5,90E+05	8,85E+05	5,90E+05	2,79E+06	3,10E+06	2,95E+06	1,30E+06	5,60E+06	4,40E+06
Q3MIH3	Ubiquitin A-52 residue ribosomal protein fusion product 1	1,23E+06	7,60E+05	1,70E+06	2,80E+06	6,60E+06	2,80E+07	1,10E+07	9,13E+06	7,30E+06	3,00E+06	2,60E+06	5,90E+07	1,08E+08	1,20E+08	1,14E+08	1,30E+08	4,20E+07	1,80E+07
Q4VB24	Histone cluster 1. H1e	b.t.	b.t.	b.t.	b.t.	b.t.	b.t.	b.t.	b.t.	b.t.	1,40E+07	2,10E+07	1,40E+07	3,33E+07	3,70E+07	3,52E+07	3,10E+07	2,79E+07	2,23E+07
Q4W4Y1	Dopamine receptor interacting protein 4	b.t.	b.t.	b.t.	b.t.	b.t.	b.t.	b.t.	b.t.	b.t.	b.t.	b.t.	b.t.	b.t.	b.t.	b.t.	1,30E+06	1,17E+06	9,36E+05
Q53F20	Acidic leucine-rich nuclear phosphoprotein 32 family, member D	b.t.	b.t.	b.t.	5,52E+06	4,60E+06	4,60E+06	b.t.	b.t.	b.t.	4,90E+06	7,35E+06	4,90E+06	b.t.	b.t.	b.t.	9,30E+06	1,10E+06	8,80E+05
Q53F35	Acidic leucine-rich nuclear phosphoprotein 32 family, member C	b.t.	b.t.	b.t.	b.t.	b.t.	b.t.	b.t.	b.t.	b.t.	2,70E+06	4,05E+06	2,70E+06	9,90E+06	1,10E+07	1,05E+07	1,60E+07	1,44E+07	1,15E+07
Q53F48	Heterogeneous nuclear ribonucleoprotein H3	b.t.	b.t.	b.t.	b.t.	b.t.	b.t.	1,23E+06	1,03E+06	8,20E+05	b.t.	b.t.	b.t.	3,87E+06	4,30E+06	4,09E+06	5,40E+06	4,86E+06	3,89E+06
Q53F64	Heterogeneous nuclear ribonucleoprotein AB	2,90E+06	2,90E+06	4,35E+06	7,16E+06	6,20E+06	5,80E+06	5,10E+06	4,25E+06	3,40E+06	1,80E+06	2,70E+06	1,80E+06	2,34E+07	2,60E+07	2,47E+07	1,80E+07	1,62E+07	2,90E+06
Q53FC7	Heat shock 70kDa protein 6 (HSP70B')	3,40E+06	5,00E+06	1,80E+06	8,39E+06	7,40E+06	6,70E+06	1,73E+06	8,50E+05	1,30E+06	8,00E+06	8,50E+06	1,40E+07	4,59E+07	5,10E+07	4,85E+07	2,70E+07	3,80E+07	3,00E+07
Q53FN7	BZW1 protein variant	b.t.	b.t.	b.t.	2,88E+06	2,64E+06	2,40E+06	b.t.	b.t.	b.t.	1,00E+06	1,00E+06	1,50E+06	4,86E+06	5,40E+06	5,13E+06	7,60E+06	7,80E+06	3,10E+06
Q53FR4	Vacuolar protein sorting 35 variant	b.t.	b.t.	b.t.	4,33E+06	6,00E+06	1,90E+06	b.t.	b.t.	b.t.	b.t.	b.t.	b.t.	9,90E+06	1,10E+07	1,05E+07	4,30E+06	6,60E+06	8,00E+06
Q53FT8	Proteasome subunit beta type-6	3,30E+05	3,30E+05	4,95E+05	2,04E+06	1,87E+06	1,70E+06	b.t.	b.t.	b.t.	3,60E+06	5,40E+06	3,60E+06	8,46E+06	9,40E+06	8,93E+06	9,00E+06	8,10E+06	5,10E+05
Q53FW2	Phosphoribosyl pyrophosphate synthetase 1	b.t.	b.t.	b.t.	b.t.	b.t.	b.t.	1,65E+06	1,38E+06	1,10E+06	3,11E+06	7,20E+05	5,50E+06	1,62E+07	1,80E+07	1,71E+07	1,10E+07	9,90E+06	7,92E+06
Q53G35	Phosphoglycerate mutase 1	6,50E+05	6,50E+05	9,75E+05	1,39E+07	1,10E+07	1,20E+07	5,56E+06	9,20E+05	5,10E+06	1,15E+07	1,90E+06	2,10E+07	4,05E+07	4,50E+07	4,28E+07	6,60E+07	3,80E+06	2,90E+06
Q53G64	Anterior gradient 2	b.t.	b.t.	b.t.	1,13E+07	1,03E+07	9,40E+06	2,31E+07	2,20E+06	2,20E+07	5,30E+06	9,00E+06	7,40E+06	8,10E+07	9,00E+07	8,55E+07	1,60E+08	3,30E+06	2,80E+06
Q53G74	Ribosomal protein L4 variant	b.t.	b.t.	b.t.	b.t.	b.t.	b.t.	b.t.	b.t.	b.t.	b.t.	b.t.	b.t.	4,59E+07	5,10E+07	4,85E+07	4,90E+07	4,41E+07	3,53E+07
Q53G99	Beta actin	6,20E+06	4,80E+06	7,60E+06	8,20E+06	2,60E+08	3,40E+07	1,60E+07	1,20E+07	1,00E+07	5,80E+07	1,30E+08	2,20E+07	7,20E+08	8,00E+08	7,60E+08	3,80E+08	6,20E+06	6,00E+08
Q53GA7	Tubulin alpha 6	b.t.	b.t.	b.t.	3,33E+06	2,60E+06	2,90E+06	4,50E+06	3,75E+06	3,00E+06	7,70E+06	4,40E+06	1,10E+07	5,31E+07	5,90E+07	5,61E+07	3,40E+07	1,90E+05	1,40E+07
Q53GN4	WD repeat domain 1, isoform	b.t.	b.t.	b.t.	b.t.	b.t.	b.t.	b.t.	b.t.	b.t.	b.t.	b.t.	b.t.	4,32E+06	4,80E+06	4,56E+06	3,30E+06	3,70E+06	4,60E+06
Q53GN6	Proteasome 26S non-ATPase subunit 13 isoform 1	b.t.	b.t.	b.t.	4,09E+06	3,00E+06	3,70E+06	b.t.	b.t.	b.t.	b.t.	b.t.	b.t.	4,23E+06	4,70E+06	4,47E+06	4,10E+06	1,20E+07	4,70E+06
Q53GX7	Threonyl-tRNA synthetase	b.t.	b.t.	b.t.	4,52E+06	5,40E+06	2,60E+06	b.t.	b.t.	b.t.	4,70E+05	4,70E+05	7,05E+05	7,74E+06	8,60E+06	8,17E+06	4,10E+06	6,70E+06	6,30E+06
Q53H34	Ribosomal protein L13a	b.t.	b.t.	b.t.	b.t.	b.t.	b.t.	b.t.	b.t.	b.t.	1,80E+06	2,70E+06	1,80E+06	3,06E+06	3,40E+06	3,23E+06	4,00E+06	3,60E+06	2,88E+06
Q53HA4	Seryl-tRNA synthetase	b.t.	b.t.	b.t.	b.t.	b.t.	b.t.	b.t.	b.t.	b.t.	1,50E+06	2,25E+06	1,50E+06	b.t.	b.t.	b.t.	1,30E+06	1,20E+06	4,70E+06
Q53HB3	Proteasome 26S ATPase subunit 1	b.t.	b.t.	b.t.	4,40E+06	5,30E+06	2,50E+06	b.t.	b.t.	b.t.	1,30E+06	1,30E+06	1,95E+06	6,75E+06	7,50E+06	7,13E+06	3,00E+06	1,00E+07	7,70E+06
Q53HD3	Ribosomal protein L18a	b.t.	b.t.	b.t.	b.t.	b.t.	b.t.	b.t.	b.t.	b.t.	5,90E+06	8,85E+06	5,90E+06	4,41E+06	4,90E+06	4,66E+06	8,30E+06	7,47E+06	5,98E+06
Q53HE7	Small nuclear ribonucleoprotein polypeptide N	b.t.	b.t.	b.t.	6,60E+06	6,05E+06	5,50E+06	b.t.	b.t.	b.t.	7,90E+06	1,19E+07	7,90E+06	1,35E+07	1,50E+07	1,43E+07	2,50E+07	1,20E+06	1,20E+06
Q53HR2	Acyl-Coenzyme A dehydrogenase	b.t.	b.t.	b.t.	2,70E+06	2,70E+06	3,51E+06	b.t.	b.t.	b.t.	b.t.	b.t.	b.t.	6,03E+06	6,70E+06	6,37E+06	4,80E+06	4,32E+06	4,80E+06
Q53HS0	Glutaminyl-tRNA synthetase variant	b.t.	b.t.	b.t.	2,25E+06	2,40E+06	1,50E+06	b.t.	b.t.	b.t.	2,30E+06	3,45E+06	2,30E+06	b.t.	b.t.	b.t.	2,30E+06	2,07E+06	1,66E+06
Q53HU0	Chaperonin containing TCP1a	b.t.	b.t.	b.t.	1,32E+06	1,21E+06	1,10E+06	b.t.	b.t.	b.t.	9,20E+05	9,20E+05	1,38E+06	3,60E+06	4,00E+06	3,80E+06	8,70E+06	7,83E+06	5,30E+06
Q53HV1	Ribosomal protein S4	b.t.	b.t.	b.t.	3,12E+06	2,86E+06	2,60E+06	b.t.	b.t.	b.t.	2,95E+06	2,50E+06	3,40E+06	2,52E+07	2,80E+07	2,66E+07	3,90E+07	3,51E+07	2,81E+07
Q53HV2	Chaperonin containing TCP1b	b.t.	b.t.	b.t.	3,00E+06	2,75E+06	2,50E+06	b.t.	b.t.	b.t.	b.t.	b.t.	b.t.	b.t.	b.t.	b.t.	6,40E+06	5,50E+06	1,10E+07
Q53Y51	D-dopachrome tautomerase	b.t.	b.t.	b.t.	7,20E+06	6,60E+06	6,00E+06	3,00E+06	2,50E+06	2,00E+06	1,70E+06	2,55E+06	1,70E+06	b.t.	b.t.	b.t.	3,50E+06	3,15E+06	2,52E+06
Q562R1	Beta-actin-like protein 2	2,58E+06	7,50E+05	4,40E+06	1,56E+07	1,43E+07	1,30E+07	b.t.	b.t.	b.t.	2,10E+06	2,10E+06	3,15E+06	b.t.	b.t.	b.t.	2,88E+07	2,59E+07	2,40E+07
Q567R6	Single-stranded DNA-binding protein	b.t.	b.t.	b.t.	6,60E+06	6,05E+06	5,50E+06	b.t.	b.t.	b.t.	6,30E+06	9,45E+06	6,30E+06	1,80E+07	2,00E+07	1,90E+07	1,70E+07	1,53E+07	1,22E+07
Q59EF6	Calpain 2, large [catalytic] subunit	b.t.	b.t.	b.t.	2,64E+06	2,42E+06	2,20E+06	b.t.	b.t.	b.t.	b.t.	b.t.	b.t.	5,13E+06	5,70E+06	5,42E+06	3,20E+06	2,88E+06	3,90E+06
Q59EG8	Proteasome 26S non-ATPase subunit 2	b.t.	b.t.	b.t.	2,77E+06	3,30E+06	1,60E+06	b.t.	b.t.	b.t.	b.t.	b.t.	b.t.	4,95E+06	5,50E+06	5,23E+06	4,10E+06	3,69E+06	6,30E+06
Q59EI4	Copine I variant	b.t.	b.t.	b.t.	b.t.	b.t.	b.t.	b.t.	b.t.	b.t.	b.t.	b.t.	b.t.	b.t.	b.t.	b.t.	1,40E+06	1,26E+06	1,30E+06
Q59EJ5	Glutathione S-transferase M3	2,20E+06	2,20E+06	3,30E+06	3,96E+06	3,63E+06	3,30E+06	1,80E+06	1,50E+06	1,20E+06	8,40E+05	8,40E+05	1,26E+06	4,32E+06	4,80E+06	4,56E+06	6,00E+06	5,40E+06	1,10E+06
Q59ET0	Glucan	b.t.	b.t.	b.t.	b.t.	b.t.	b.t.	b.t.	b.t.	b.t.	b.t.	b.t.	b.t.	b.t.	b.t.	b.t.	3,40E+06	3,40E+06	2,72E+06
Q59ET3	Chaperonin containing TCP1, subunit 6A	b.t.	b.t.	b.t.	2,89E+06	4,10E+06	1,20E+06	2,10E+06	1,75E+06	1,40E+06	1,00E+06	1,00E+06	1,50E+06	9,90E+06	1,10E+07	1,05E+07	8,70E+06	5,10E+06	5,30E+06
Q59F66	DEAD box polypeptide 17 isoform p82	b.t.	b.t.	b.t.	b.t.	b.t.	b.t.	b.t.	b.t.	b.t.	b.t.	b.t.	b.t.	b.t.	b.t.	b.t.	1,90E+06	4,80E+06	3,90E+06
Q59FF0	EBNA-2 co-activator	b.t.	b.t.	b.t.	b.t.	b.t.	b.t.	b.t.	b.t.	b.t.	5,10E+05	5,10E+05	7,65E+05	7,47E+06	8,30E+06	7,89E+06	6,40E+06	1,10E+07	4,80E+06
Q59FI4	Importin 4	b.t.	b.t.	b.t.	3,20E+06	3,20E+06	4,16E+06	b.t.	b.t.	b.t.	b.t.	b.t.	b.t.	b.t.	b.t.	b.t.	2,20E+06	1,98E+06	1,20E+06
Q59FR8	Galectin-1a	1,10E+06	1,10E+06	1,65E+06	1,12E+07	1,10E+07	8,20E+06	6,75E+06	5,63E+06	4,50E+06	4,00E+07	6,00E+07	4,00E+07	1,53E+08	1,70E+08	1,62E+08	1,50E+08	7,40E+06	5,92E+06
Q59G24	Activated RNA polymerase II transcription cofactor 4	b.t.	b.t.	b.t.	1,68E+06	1,54E+06	1,40E+06	1,95E+06	1,63E+06	1,30E+06	7,40E+06	1,11E+07	7,40E+06	1,71E+07	1,90E+07	1,81E+07	1,70E+07	1,53E+07	1,22E+07
Q59G75	Isoleucyl-tRNA synthetase, cytoplasmic variant	b.t.	b.t.	b.t.	5,85E+06	6,10E+06	4,00E+06	b.t.	b.t.	b.t.	2,40E+06	3,70E+06	2,60E+06	9,90E+06	1,10E+07	1,05E+07	4,10E+06	9,30E+06	1,30E+07
Q59GA1	Splicing factor, arginine/serine-rich 10	b.t.	b.t.	b.t.	b.t.	b.t.	b.t.	b.t.	b.t.	b.t.	b.t.	b.t.	b.t.	b.t.	b.t.	b.t.	9,00E+06	8,10E+06	6,48E+06
Q59GB4	Dihydropyrimidinase-like 2	b.t.	b.t.	b.t.	b.t.	b.t.	b.t.	b.t.	b.t.	b.t.	2,35E+06	1,10E+06	3,60E+06	1,17E+07	1,30E+07	1,24E+07	5,30E+06	4,77E+06	7,70E+06
Q59GE4	Ribosomal protein S10	b.t.	b.t.	b.t.	9,48E+06	8,69E+06	7,90E+06	8,25E+06	6,88E+06	5,50E+06	1,20E+06	1,80E+06	2,40E+06	4,50E+06	5,00E+06	4,75E+06	7,90E+07	7,11E+07	5,69E+07
Q59GK9	Ribosomal protein L21	b.t.	b.t.	b.t.	b.t.	b.t.	b.t.	b.t.	b.t.	b.t.	3,70E+06	5,55E+06	3,70E+06	b.t.	b.t.	b.t.	b.t.	b.t.	b.t.
Q59GS3	Proteasome 26S ATPase subunit 5 variant	b.t.	b.t.	b.t.	1,32E+06	1,21E+06	1,10E+06	b.t.	b.t.	b.t.	b.t.	b.t.	b.t.	7,20E+06	8,00E+06	7,60E+06	1,90E+06	5,30E+06	5,40E+06
Q59GW1	High-mobility group box 1	b.t.	b.t.	b.t.	b.t.	b.t.	b.t.	b.t.	b.t.	b.t.	9,10E+06	1,37E+07	9,10E+06	1,71E+07	1,90E+07	1,81E+07	1,10E+07	9,90E+06	7,92E+06
Q59GX6	DEAD/H (Asp-Glu-Ala-Asp/His) box polypeptide 3	b.t.	b.t.	b.t.	3,96E+06	3,63E+06	3,30E+06	b.t.	b.t.	b.t.	1,40E+06	1,40E+06	2,10E+06	1,71E+07	1,90E+07	1,81E+07	2,90E+06	2,61E+06	1,30E+05
Q59GX9	Ribosomal protein L5 variant	b.t.	b.t.	b.t.	b.t.	b.t.	b.t.	1,95E+06	1,63E+06	1,30E+06	1,10E+07	1,65E+07	1,10E+07	7,20E+06	8,00E+06	7,60E+06	1,30E+07	1,17E+07	9,36E+06

Q59GY3	Arginine/serine-rich splicing factor 6 variant	b.t.	b.t.	b.t.	b.t.	b.t.	b.t.	b.t.	b.t.	b.t.	b.t.	b.t.	b.t.	1,08E+07	1,20E+07	1,14E+07	5,40E+06	4,86E+06	3,89E+06
Q59H77	T-complex protein 1 subunit gamma	b.t.	b.t.	b.t.	6,48E+06	5,94E+06	5,40E+06	b.t.	b.t.	b.t.	1,46E+06	6,20E+05	2,30E+06	1,17E+07	1,30E+07	1,24E+07	6,90E+06	4,50E+06	1,20E+07
Q59HH3	Phosphoribosylglycinamide formyltransferase/phosphoribosylglycinar	b.t.	b.t.	b.t.	2,80E+06	2,80E+06	3,64E+06	b.t.	b.t.	b.t.	5,90E+05	8,85E+05	5,90E+05	2,79E+06	3,10E+06	2,95E+06	1,30E+06	5,60E+06	4,40E+06
Q5CAQ5	Tumor rejection antigen (Gp96) 1	2,40E+06	2,40E+06	3,60E+06	2,00E+06	9,40E+06	2,20E+07	3,15E+06	1,70E+06	2,30E+06	6,00E+06	1,30E+07	1,00E+07	1,17E+08	1,30E+08	1,24E+08	6,70E+07	9,60E+07	1,10E+08
Q5JP53	Tubulin beta chain	b.t.	b.t.	b.t.	b.t.	b.t.	b.t.	b.t.	b.t.	b.t.	4,30E+06	3,70E+06	4,90E+06	3,33E+07	3,70E+07	3,52E+07	2,80E+07	2,52E+07	9,10E+05
Q5JR94	40S ribosomal protein S8	b.t.	b.t.	b.t.	b.t.	b.t.	b.t.	b.t.	b.t.	b.t.	5,60E+05	5,60E+05	8,40E+05	b.t.	b.t.	b.t.	b.t.	b.t.	b.t.
Q5JTH9	RRP12-like protein	b.t.	b.t.	b.t.	b.t.	b.t.	b.t.	b.t.	b.t.	b.t.	b.t.	b.t.	b.t.	b.t.	b.t.	b.t.	1,90E+06	6,40E+05	5,12E+05
Q5JYR7	Dolichyl-diphosphooligosaccharide-protein glycosyltransferase subuni	b.t.	b.t.	b.t.	2,52E+06	2,31E+06	2,10E+06	b.t.	b.t.	b.t.	b.t.	b.t.	b.t.	b.t.	b.t.	b.t.	3,10E+06	2,79E+06	1,50E+06
Q5M7Z5	GRHPR protein	b.t.	b.t.	b.t.	b.t.	b.t.	b.t.	b.t.	b.t.	b.t.	1,90E+06	2,85E+06	1,90E+06	1,53E+07	1,70E+07	1,62E+07	b.t.	b.t.	b.t.
Q5M7Z9	TARS protein	b.t.	b.t.	b.t.	4,52E+06	5,40E+06	2,60E+06	b.t.	b.t.	b.t.	4,70E+05	4,70E+05	7,05E+05	7,74E+06	8,60E+06	8,17E+06	4,10E+06	6,70E+06	6,30E+06
Q5PY61	Polyubiquitin-C	1,23E+06	7,60E+05	1,70E+06	2,80E+06	6,60E+06	2,80E+07	1,10E+07	9,13E+06	7,30E+06	3,00E+06	2,60E+06	5,90E+07	1,08E+08	1,20E+08	1,14E+08	1,30E+08	4,20E+07	1,80E+07
Q5QNW6	Histone H2B type 2-F	b.t.	b.t.	b.t.	b.t.	b.t.	b.t.	b.t.	b.t.	b.t.	2,80E+06	4,20E+06	2,80E+06	1,08E+07	1,20E+07	1,14E+07	b.t.	b.t.	b.t.
Q5R210	Carbamoylphosphate synthetase I	3,35E+06	5,00E+06	1,70E+06	4,64E+07	5,50E+07	2,70E+07	1,92E+06	8,40E+05	1,50E+06	5,00E+06	4,40E+06	1,90E+07	7,38E+07	8,20E+07	7,79E+07	2,70E+07	2,10E+07	4,90E+07
Q5RKT7	Ribosomal protein S27a	1,23E+06	7,60E+05	1,70E+06	2,80E+06	6,60E+06	2,80E+07	1,10E+07	9,13E+06	7,30E+06	3,00E+06	2,60E+06	5,90E+07	1,08E+08	1,20E+08	1,14E+08	1,30E+08	4,20E+07	1,80E+07
Q5SU16	Beta 5-tubulin	b.t.	b.t.	b.t.	b.t.	b.t.	b.t.	b.t.	b.t.	b.t.	4,30E+06	3,70E+06	4,90E+06	3,33E+07	3,70E+07	3,52E+07	2,80E+07	2,52E+07	9,10E+05
Q5T5C7	Serine-tRNA ligase, cytoplasmic	b.t.	b.t.	b.t.	b.t.	b.t.	b.t.	b.t.	b.t.	b.t.	1,50E+06	2,25E+06	1,50E+06	b.t.	b.t.	b.t.	1,30E+06	1,20E+06	4,70E+06
Q5T7C4	High mobility group protein B1	b.t.	b.t.	b.t.	b.t.	b.t.	b.t.	b.t.	b.t.	b.t.	9,10E+06	1,37E+07	9,10E+06	1,71E+07	1,90E+07	1,81E+07	1,10E+07	9,90E+06	7,92E+06
Q5TB52	3'-phosphoadenosine 5'-phosphosulfate synthase 2	b.t.	b.t.	b.t.	2,76E+06	2,53E+06	2,30E+06	b.t.	b.t.	b.t.	2,20E+06	1,00E+06	3,40E+06	2,97E+06	3,30E+06	3,14E+06	7,00E+06	6,30E+06	2,80E+06
Q5TCI8	Prelamin-A/C	b.t.	b.t.	b.t.	b.t.	b.t.	b.t.	b.t.	b.t.	b.t.	9,70E+06	1,46E+07	9,70E+06	2,16E+07	2,40E+07	2,28E+07	b.t.	b.t.	b.t.
Q5TEC6	Histone H3	6,65E+06	2,30E+06	1,10E+07	b.t.	b.t.	b.t.	1,80E+07	1,50E+07	1,20E+07	3,90E+06	1,94E+07	3,10E+07	b.t.	b.t.	b.t.	3,20E+07	2,88E+07	2,30E+07
Q5U077	L-lactate dehydrogenase	b.t.	b.t.	b.t.	8,22E+06	3,00E+06	9,60E+06	2,80E+06	1,40E+06	2,10E+06	3,90E+06	3,10E+06	3,20E+07	1,08E+08	1,20E+08	1,14E+08	6,10E+07	1,20E+07	4,00E+06
Q5U0C3	RAP1A, member of RAS oncogene family	b.t.	b.t.	b.t.	b.t.	b.t.	b.t.	b.t.	b.t.	b.t.	b.t.	b.t.	b.t.	5,67E+06	6,30E+06	5,99E+06	3,00E+06	2,70E+06	2,16E+06
Q5U8W9	Protein arginine methyltransferase 1	b.t.	b.t.	b.t.	b.t.	b.t.	b.t.	b.t.	b.t.	b.t.	b.t.	b.t.	b.t.	b.t.	b.t.	b.t.	1,20E+07	1,20E+07	9,60E+06
Q6FGB3	PCBD protein	b.t.	b.t.	b.t.	b.t.	b.t.	b.t.	b.t.	b.t.	b.t.	b.t.	b.t.	b.t.	b.t.	b.t.	b.t.	4,00E+06	3,60E+06	2,88E+06
Q6FGD7	TBCA protein	b.t.	b.t.	b.t.	b.t.	b.t.	b.t.	b.t.	b.t.	b.t.	2,20E+06	3,30E+06	2,20E+06	b.t.	b.t.	b.t.	b.t.	b.t.	b.t.
Q6FGH9	DNCL1 protein	b.t.	b.t.	b.t.	b.t.	b.t.	b.t.	b.t.	b.t.	b.t.	b.t.	b.t.	b.t.	b.t.	b.t.	b.t.	1,40E+07	1,26E+07	1,01E+07
Q6FHP5	PHB protein	b.t.	b.t.	b.t.	1,35E+06	4,50E+05	1,60E+06	1,07E+06	8,88E+05	7,10E+05	3,70E+06	5,55E+06	3,70E+06	1,44E+07	1,60E+07	1,52E+07	1,60E+07	1,44E+07	1,15E+07
Q6FHU3	PSME1 protein	b.t.	b.t.	b.t.	b.t.	b.t.	b.t.	b.t.	b.t.	b.t.	3,03E+06	4,50E+05	5,60E+06	7,92E+06	8,80E+06	8,36E+06	1,30E+07	1,17E+07	3,90E+05
Q6FHV6	ENO2 protein	b.t.	b.t.	b.t.	6,61E+06	7,20E+06	4,30E+06	b.t.	b.t.	b.t.	6,60E+06	9,90E+06	6,60E+06	6,66E+06	7,40E+06	7,03E+06	2,00E+06	1,80E+06	1,44E+06
Q6FHX6	FEN1 protein	b.t.	b.t.	b.t.	b.t.	b.t.	b.t.	b.t.	b.t.	b.t.	b.t.	b.t.	b.t.	9,90E+06	1,10E+07	1,05E+07	b.t.	b.t.	b.t.
Q6IAX2	RPL21 protein	b.t.	b.t.	b.t.	b.t.	b.t.	b.t.	b.t.	b.t.	b.t.	3,70E+06	5,55E+06	3,70E+06	b.t.	b.t.	b.t.	b.t.	b.t.	b.t.
Q6IAX5	Eukaryotic translation initiation factor 3 subunit E	b.t.	b.t.	b.t.	b.t.	b.t.	b.t.	b.t.	b.t.	b.t.	b.t.	b.t.	b.t.	b.t.	b.t.	b.t.	2,90E+06	3,50E+06	2,80E+06
Q6IBA2	PC4 protein	b.t.	b.t.	b.t.	1,68E+06	1,54E+06	1,40E+06	1,95E+06	1,63E+06	1,30E+06	7,40E+06	1,11E+07	7,40E+06	1,71E+07	1,90E+07	1,81E+07	1,70E+07	1,53E+07	1,22E+07
Q6IBG5	MYL6 protein	b.t.	b.t.	b.t.	b.t.	b.t.	b.t.	b.t.	b.t.	b.t.	3,50E+06	5,25E+06	3,50E+06	6,39E+06	7,10E+06	6,75E+06	1,40E+07	1,26E+07	1,01E+07
Q6IBH6	RPL26 protein	b.t.	b.t.	b.t.	b.t.	b.t.	b.t.	b.t.	b.t.	b.t.	3,80E+06	5,70E+06	3,80E+06	b.t.	b.t.	b.t.	7,60E+06	6,84E+06	5,47E+06
Q6IBM8	U5-116KD protein	b.t.	b.t.	b.t.	2,30E+06	2,30E+06	2,99E+06	b.t.	b.t.	b.t.	b.t.	b.t.	b.t.	b.t.	b.t.	b.t.	2,20E+06	1,98E+06	1,58E+06
Q6IBN0	PSMD3 protein	b.t.	b.t.	b.t.	7,44E+05	6,82E+05	6,20E+05	b.t.	b.t.	b.t.	b.t.	b.t.	b.t.	b.t.	b.t.	b.t.	2,30E+06	5,10E+06	5,20E+06
Q6IBN1	HNRPK protein	1,70E+06	1,70E+06	2,55E+06	2,16E+07	1,98E+07	1,80E+07	4,85E+06	1,10E+06	4,30E+06	1,00E+06	1,70E+07	9,30E+06	4,05E+07	4,50E+07	4,28E+07	7,90E+07	1,40E+06	1,30E+06
Q6IBQ5	FUS protein	b.t.	b.t.	b.t.	b.t.	b.t.	b.t.	b.t.	b.t.	b.t.	b.t.	b.t.	b.t.	1,17E+07	1,30E+07	1,24E+07	b.t.	b.t.	b.t.
Q6IBR0	Dolichyl-diphosphooligosaccharide-protein glycosyltransferase subuni	b.t.	b.t.	b.t.	4,54E+06	6,70E+06	1,70E+06	b.t.	b.t.	b.t.	b.t.	b.t.	b.t.	7,38E+06	8,20E+06	7,79E+06	1,08E+07	9,72E+06	9,00E+06
Q6IBR2	FARSLA protein	b.t.	b.t.	b.t.	b.t.	b.t.	b.t.	2,10E+06	1,75E+06	1,40E+06	b.t.	b.t.	b.t.	5,49E+06	6,10E+06	5,80E+06	3,90E+06	3,51E+06	2,81E+06
Q6IBS5	DLST protein	b.t.	b.t.	b.t.	b.t.	b.t.	b.t.	b.t.	b.t.	b.t.	1,60E+06	2,40E+06	1,60E+06	7,74E+06	8,60E+06	8,17E+06	9,60E+06	8,64E+06	6,91E+06
Q6IBU0	EIF5 protein	b.t.	b.t.	b.t.	2,33E+06	2,70E+06	1,40E+06	b.t.	b.t.	b.t.	b.t.	b.t.	b.t.	1,26E+07	1,40E+07	1,33E+07	7,90E+06	7,11E+06	5,69E+06
Q6ICN0	GRB2 protein	b.t.	b.t.	b.t.	b.t.	b.t.	b.t.	b.t.	b.t.	b.t.	b.t.	b.t.	b.t.	9,90E+06	1,10E+07	1,05E+07	b.t.	b.t.	b.t.
Q6LBS1	SmB /B' autoimmune antigene	b.t.	b.t.	b.t.	6,60E+06	6,05E+06	5,50E+06	b.t.	b.t.	b.t.	7,90E+06	1,19E+07	7,90E+06	1,35E+07	1,50E+07	1,43E+07	2,50E+07	1,20E+06	1,20E+06
Q6LET3	HPRT1 protein	b.t.	b.t.	b.t.	6,48E+06	5,94E+06	5,40E+06	2,10E+06	1,75E+06	1,40E+06	2,90E+06	4,35E+06	2,90E+06	b.t.	b.t.	b.t.	1,20E+07	1,08E+07	8,64E+06
Q6NTA2	HNRNPL protein	b.t.	b.t.	b.t.	3,84E+06	3,52E+06	3,20E+06	b.t.	b.t.	b.t.	3,60E+06	5,40E+06	3,60E+06	1,08E+07	1,20E+07	1,14E+07	2,00E+07	1,80E+07	1,44E+07
Q6NVW9	SNRPA1 protein	b.t.	b.t.	b.t.	4,56E+06	4,18E+06	3,80E+06	b.t.	b.t.	b.t.	b.t.	b.t.	b.t.	2,52E+06	2,80E+06	2,66E+06	b.t.	b.t.	b.t.
Q6NWZ1	CKAP4 protein	b.t.	b.t.	b.t.	b.t.	b.t.	b.t.	b.t.	b.t.	b.t.	b.t.	b.t.	b.t.	2,88E+06	3,20E+06	3,04E+06	4,40E+06	3,96E+06	1,00E+07
Q6NXR8	40S ribosomal protein S3a	b.t.	b.t.	b.t.	b.t.	b.t.	b.t.	b.t.	b.t.	b.t.	1,20E+07	1,80E+07	1,20E+07	1,17E+07	1,30E+07	1,24E+07	1,80E+07	1,62E+07	1,30E+07
Q6P2H8	Transmembrane protein 53 transport domain	b.t.	b.t.	b.t.	b.t.	b.t.	b.t.	b.t.	b.t.	b.t.	b.t.	b.t.	b.t.	b.t.	b.t.	b.t.	6,60E+06	5,94E+06	4,75E+06
Q6P2Q9	Pre-mRNA-processing-splicing factor 8	3,10E+05	4,65E+05	3,10E+05	4,20E+06	4,20E+06	5,46E+06	b.t.	b.t.	b.t.	b.t.	b.t.	b.t.	3,42E+06	3,80E+06	3,61E+06	6,84E+06	6,16E+06	5,70E+06
Q6PCE3	Glucose 1,6-bisphosphate synthase	b.t.	b.t.	b.t.	2,35E+06	2,60E+06	1,50E+06	b.t.	b.t.	b.t.	b.t.	b.t.	b.t.	3,69E+06	4,10E+06	3,90E+06	6,36E+06	5,72E+06	5,30E+06
Q6PIN5	PA2G4 protein B	b.t.	b.t.	b.t.	b.t.	b.t.	b.t.	b.t.	b.t.	b.t.	1,50E+06	1,50E+06	2,25E+06	1,17E+07	1,30E+07	1,24E+07	1,00E+07	9,00E+06	1,10E+07
Q6PJY1	FUBP1 protein	b.t.	b.t.	b.t.	b.t.	b.t.	b.t.	b.t.	b.t.	b.t.	b.t.	b.t.	b.t.	b.t.	b.t.	b.t.	1,70E+07	1,53E+07	1,22E+07
Q6PKA6	ALDH3A1 protein	2,15E+06	2,30E+06	2,00E+06	4,27E+07	6,30E+07	1,60E+07	3,90E+06	3,25E+06	2,60E+06	1,20E+07	5,80E+06	1,10E+07	1,53E+08	1,70E+08	1,62E+08	4,50E+07	3,40E+07	1,10E+08
Q6PKH8	ANP32A protein	b.t.	b.t.	b.t.	6,72E+06	6,16E+06	5,60E+06	2,85E+06	2,38E+06	1,90E+06	4,10E+06	6,15E+06	4,10E+06	4,50E+06	5,00E+06	4,75E+06	2,50E+07	2,25E+07	1,80E+07
Q71RH4	FP1047	b.t.	b.t.	b.t.	b.t.	b.t.	b.t.	b.t.	b.t.	b.t.	2,09E+06	2,80E+05	3,90E+06	b.t.	b.t.	b.t.	6,10E+06	5,49E+06	1,30E+06
Q71U36	Tubulin alpha-1A	b.t.	b.t.	b.t.	2,63E+06	2,60E+06	1,90E+06	4,50E+06	3,75E+06	3,00E+06	7,70E+06	4,40E+06	1,10E+07	5,31E+07	5,90E+07	5,61E+07	1,10E+07	1,90E+05	1,40E+07
Q76LA1	CSTB protein	b.t.	b.t.	b.t.	2,52E+06	2,31E+06	2,10E+06	b.t.	b.t.	b.t.	b.t.	b.t.	b.t.	1,35E+07	1,50E+07	1,43E+07	2,00E+07	1,80E+07	1,44E+07
Q7KZX8	G1 to S phase transition 1	b.t.	b.t.	b.t.	b.t.	b.t.	b.t.	b.t.	b.t.	b.t.	b.t.	b.t.	b.t.	b.t.	b.t.	b.t.	4,40E+06	1,20E+07	8,60E+06
Q7Z3X3	N-acetylglucosamine-6-sulfatase	b.t.	b.t.	b.t.	4,03E+06	4,00E+06	2,90E+06	b.t.	b.t.	b.t.	b.t.	b.t.	b.t.	b.t.	b.t.	b.t.	b.t.	b.t.	b.t.
Q7Z4X0	MO25-like protein	b.t.	b.t.	b.t.	2,08E+06	1,50E+06	1,90E+06	b.t.	b.t.	b.t.	b.t.	b.t.	b.t.	4,41E+06	4,90E+06	4,66E+06	b.t.	b.t.	b.t.
Q7Z726	Importin subunit alpha	b.t.	b.t.	b.t.	3,40E+06	3,40E+06	4,42E+06	b.t.	b.t.	b.t.	b.t.	b.t.	b.t.	b.t.	b.t.	b.t.	2,50E+06	3,50E+06	2,80E+06

Q86SZ7	Full-length cDNA clone CS0DJ015YJ12	b.t.	b.t.	b.t.	6,84E+05	6,27E+05	5,70E+05	b.t.	b.t.	b.t.	2,60E+06	1,70E+06	3,50E+06	5,22E+06	5,80E+06	5,51E+06	9,30E+06	8,37E+06	6,70E+06
Q86UY0	TXNDC5 protein	b.t.	b.t.	b.t.	2,70E+06	2,60E+06	2,00E+06	b.t.	b.t.	b.t.	1,40E+06	3,10E+06	4,70E+06	1,98E+07	2,20E+07	2,09E+07	5,90E+06	1,10E+07	5,70E+06
Q86VG2	Splicing factor proline/glutamine-rich	b.t.	b.t.	b.t.	2,52E+06	2,31E+06	2,10E+06	2,40E+06	2,00E+06	1,60E+06	1,33E+06	1,70E+06	9,60E+05	4,86E+06	5,40E+06	5,13E+06	7,90E+06	7,11E+06	5,69E+06
Q86VP6	Cullin-associated NEDD8-dissociated protein 1	b.t.	b.t.	b.t.	5,45E+06	9,80E+06	7,90E+05	b.t.	b.t.	b.t.	b.t.	b.t.	b.t.	5,85E+06	6,50E+06	6,18E+06	3,70E+06	4,10E+06	7,00E+06
Q8IWP6	Class IVb beta tubulin	b.t.	b.t.	b.t.	b.t.	b.t.	b.t.	b.t.	b.t.	b.t.	1,80E+06	1,80E+06	2,70E+06	b.t.	b.t.	b.t.	b.t.	b.t.	b.t.
Q8IZ29	Tubulin, beta 2C	b.t.	b.t.	b.t.	7,80E+05	7,15E+05	6,50E+05	b.t.	b.t.	b.t.	2,20E+06	1,10E+06	3,30E+06	2,88E+07	3,20E+07	3,04E+07	2,88E+06	2,59E+06	2,40E+06
Q8N163	Cell cycle and apoptosis regulator protein 2	b.t.	b.t.	b.t.	1,68E+06	1,54E+06	1,40E+06	1,10E+07	5,31E+07	5,90E+07	b.t.	b.t.	b.t.	b.t.	b.t.	b.t.	5,04E+06	5,60E+06	5,32E+06
Q8NBS9	Thioredoxin domain-containing protein 5	b.t.	b.t.	b.t.	2,70E+06	2,60E+06	2,00E+06	b.t.	b.t.	b.t.	1,40E+06	3,10E+06	4,70E+06	1,98E+07	2,20E+07	2,09E+07	5,90E+06	1,10E+07	5,70E+06
Q8NCW5	NAD(P)H-hydrate epimerase	b.t.	b.t.	b.t.	5,16E+06	4,73E+06	4,30E+06	2,25E+06	1,88E+06	1,50E+06	6,60E+06	9,90E+06	6,60E+06	1,35E+07	1,50E+07	1,43E+07	1,10E+07	9,90E+06	7,92E+06
Q8TB01	Similar to cytoskeleton-associated protein 4	b.t.	b.t.	b.t.	b.t.	b.t.	b.t.	b.t.	b.t.	b.t.	b.t.	b.t.	b.t.	2,88E+06	3,20E+06	3,04E+06	4,40E+06	3,96E+06	1,00E+07
Q8TC62	Septin 7	b.t.	b.t.	b.t.	b.t.	b.t.	b.t.	b.t.	b.t.	b.t.	b.t.	b.t.	b.t.	9,00E+06	1,00E+07	9,50E+06	b.t.	b.t.	b.t.
Q8TDN6	Ribosome biogenesis protein BRX1	b.t.	b.t.	b.t.	1,20E+06	1,20E+06	1,56E+06	b.t.	b.t.	b.t.	b.t.	b.t.	b.t.	6,66E+06	7,40E+06	7,03E+06	7,20E+06	6,48E+06	5,18E+06
Q8WUI6	COPG protein	b.t.	b.t.	b.t.	1,01E+06	9,24E+05	8,40E+05	2,55E+06	2,13E+06	1,70E+06	b.t.	b.t.	b.t.	3,78E+06	4,20E+06	3,99E+06	7,40E+06	7,70E+06	6,16E+06
Q8WUM4	Programmed cell death 6-interacting protein	b.t.	b.t.	b.t.	b.t.	b.t.	b.t.	b.t.	b.t.	b.t.	b.t.	b.t.	b.t.	b.t.	b.t.	b.t.	1,30E+06	1,17E+06	9,36E+05
Q92499	ATP-dependent RNA helicase DDX1	b.t.	b.t.	b.t.	b.t.	b.t.	b.t.	b.t.	b.t.	b.t.	b.t.	b.t.	b.t.	b.t.	b.t.	b.t.	4,80E+06	1,20E+06	9,60E+05
Q92616	eIF-2-alpha kinase activator GCN1	b.t.	b.t.	b.t.	b.t.	b.t.	b.t.	b.t.	b.t.	b.t.	b.t.	b.t.	b.t.	b.t.	b.t.	b.t.	2,90E+06	2,61E+06	2,09E+06
Q92688	Acidic leucine-rich nuclear phosphoprotein 32 family member B	b.t.	b.t.	b.t.	b.t.	b.t.	b.t.	b.t.	b.t.	b.t.	2,70E+06	4,05E+06	2,70E+06	9,90E+06	1,10E+07	1,05E+07	1,60E+07	1,44E+07	1,15E+07
Q92945	Far upstream element-binding protein 2	b.t.	b.t.	b.t.	b.t.	b.t.	b.t.	b.t.	b.t.	b.t.	7,90E+05	7,90E+05	1,19E+06	9,00E+06	1,00E+07	9,50E+06	3,60E+06	3,24E+06	4,30E+06
Q93009	Ubiquitin carboxyl-terminal hydrolase 7	b.t.	b.t.	b.t.	3,90E+06	3,90E+06	5,07E+06	b.t.	b.t.	b.t.	b.t.	b.t.	b.t.	b.t.	b.t.	b.t.	3,90E+06	3,51E+06	2,81E+06
Q93079	Histone H2B type 1-H	b.t.	b.t.	b.t.	b.t.	b.t.	b.t.	b.t.	b.t.	b.t.	2,80E+06	4,20E+06	2,80E+06	1,08E+07	1,20E+07	1,14E+07	b.t.	b.t.	b.t.
Q96BA7	HNRPU protein	b.t.	b.t.	b.t.	8,70E+06	2,00E+06	1,10E+07	8,70E+06	7,25E+06	5,80E+06	3,40E+06	8,90E+06	1,10E+07	4,32E+07	4,80E+07	4,56E+07	2,70E+07	4,10E+06	6,30E+06
Q96BS4	FBL protein	b.t.	b.t.	b.t.	b.t.	b.t.	b.t.	b.t.	b.t.	b.t.	b.t.	b.t.	b.t.	9,90E+06	1,10E+07	1,05E+07	1,20E+07	1,08E+07	8,64E+06
Q96C19	EF-hand domain-containing protein D2	b.t.	b.t.	b.t.	b.t.	b.t.	b.t.	b.t.	b.t.	b.t.	3,70E+06	5,55E+06	3,70E+06	8,46E+06	9,40E+06	8,93E+06	8,80E+06	7,92E+06	6,34E+06
Q96DG6	Carboxymethylenebutenolidase	b.t.	b.t.	b.t.	2,76E+06	2,53E+06	2,30E+06	b.t.	b.t.	b.t.	b.t.	b.t.	b.t.	8,01E+06	8,90E+06	8,46E+06	2,37E+07	3,20E+07	1,10E+07
Q96FW1	Ubiquitin thioesterase OTUB1	b.t.	b.t.	b.t.	3,48E+06	3,19E+06	2,90E+06	b.t.	b.t.	b.t.	b.t.	b.t.	b.t.	b.t.	b.t.	b.t.	2,90E+06	2,61E+06	2,09E+06
Q96H31	UBC protein	1,23E+06	7,60E+05	1,70E+06	2,80E+06	6,60E+06	2,80E+07	1,10E+07	9,13E+06	7,30E+06	3,00E+06	2,60E+06	5,90E+07	1,08E+08	1,20E+08	1,14E+08	1,30E+08	4,20E+07	1,80E+07
Q96HE7	ERO1-like protein	3,50E+06	3,50E+06	5,25E+06	9,30E+06	1,30E+07	4,00E+06	b.t.	b.t.	b.t.	3,10E+06	2,90E+06	3,30E+06	2,16E+07	2,40E+07	2,28E+07	9,20E+06	1,60E+07	1,70E+07
Q96IR1	RPS4X protein	b.t.	b.t.	b.t.	3,12E+06	2,86E+06	2,60E+06	b.t.	b.t.	b.t.	2,95E+06	2,50E+06	3,40E+06	2,52E+07	2,80E+07	2,66E+07	3,90E+07	3,51E+07	2,81E+07
Q96KP4	Cytosolic non-specific dipeptidase	b.t.	b.t.	b.t.	1,87E+06	2,40E+06	9,50E+05	b.t.	b.t.	b.t.	1,35E+06	7,00E+05	2,00E+06	8,82E+06	9,80E+06	9,31E+06	3,10E+06	2,79E+06	5,90E+06
Q96P70	Importin-9	b.t.	b.t.	b.t.	4,60E+06	4,60E+06	5,98E+06	b.t.	b.t.	b.t.	b.t.	b.t.	b.t.	b.t.	b.t.	b.t.	2,52E+06	2,27E+06	2,10E+06
Q96QK1	Vacuolar protein sorting-associated protein 35	b.t.	b.t.	b.t.	4,33E+06	6,00E+06	1,90E+06	b.t.	b.t.	b.t.	b.t.	b.t.	b.t.	9,90E+06	1,10E+07	1,05E+07	4,30E+06	6,60E+06	8,00E+06
Q96QL0	Ribosomal protein L3	b.t.	b.t.	b.t.	b.t.	b.t.	b.t.	b.t.	b.t.	b.t.	7,00E+06	1,05E+07	7,00E+06	7,11E+06	7,90E+06	7,51E+06	2,10E+07	1,89E+07	1,51E+07
Q99460	26S proteasome non-ATPase regulatory subunit 1	b.t.	b.t.	b.t.	5,50E+06	5,50E+06	7,15E+06	b.t.	b.t.	b.t.	b.t.	b.t.	b.t.	b.t.	b.t.	b.t.	4,60E+06	9,30E+06	7,00E+06
Q99497	Protein deglycase DJ-1	2,00E+06	2,00E+06	3,00E+06	1,87E+06	1,50E+06	1,60E+06	b.t.	b.t.	b.t.	1,10E+07	1,65E+07	1,10E+07	2,07E+07	2,30E+07	2,19E+07	3,90E+07	3,51E+07	7,60E+05
Q99536	Synaptic vesicle membrane protein VAT-1 homolog	2,20E+06	3,30E+06	2,20E+06	b.t.	b.t.	b.t.	b.t.	b.t.	b.t.	b.t.	b.t.	b.t.	b.t.	b.t.	b.t.	1,00E+07	9,00E+06	7,20E+06
Q99829	Copine-1	b.t.	b.t.	b.t.	b.t.	b.t.	b.t.	b.t.	b.t.	b.t.	b.t.	b.t.	b.t.	b.t.	b.t.	b.t.	1,40E+06	1,26E+06	1,30E+06
Q99832	T-complex protein 1 subunit eta	b.t.	b.t.	b.t.	3,00E+06	2,75E+06	2,50E+06	b.t.	b.t.	b.t.	b.t.	b.t.	b.t.	b.t.	b.t.	b.t.	6,40E+06	5,50E+06	1,10E+07
Q99873	Protein arginine N-methyltransferase 1	b.t.	b.t.	b.t.	b.t.	b.t.	b.t.	b.t.	b.t.	b.t.	b.t.	b.t.	b.t.	b.t.	b.t.	b.t.	1,20E+07	1,20E+07	9,60E+06
Q99877	Histone H2B type 1-N	b.t.	b.t.	b.t.	b.t.	b.t.	b.t.	b.t.	b.t.	b.t.	2,80E+06	4,20E+06	2,80E+06	1,08E+07	1,20E+07	1,14E+07	b.t.	b.t.	b.t.
Q99879	Histone H2B type 1-M	b.t.	b.t.	b.t.	b.t.	b.t.	b.t.	b.t.	b.t.	b.t.	2,80E+06	4,20E+06	2,80E+06	1,08E+07	1,20E+07	1,14E+07	b.t.	b.t.	b.t.
Q99880	Histone H2B type 1-L	b.t.	b.t.	b.t.	b.t.	b.t.	b.t.	b.t.	b.t.	b.t.	2,80E+06	4,20E+06	2,80E+06	1,08E+07	1,20E+07	1,14E+07	b.t.	b.t.	b.t.
Q9BQE3	Tubulin alpha-1C	b.t.	b.t.	b.t.	3,33E+06	2,60E+06	2,90E+06	4,50E+06	3,75E+06	3,00E+06	7,70E+06	4,40E+06	1,10E+07	5,31E+07	5,90E+07	5,61E+07	3,40E+07	1,90E+05	1,40E+07
Q9BQG0	Myb-binding protein 1A	b.t.	b.t.	b.t.	1,80E+06	1,65E+06	1,50E+06	b.t.	b.t.	b.t.	b.t.	b.t.	b.t.	b.t.	b.t.	b.t.	b.t.	b.t.	b.t.
Q9BR63	FARSB protein	b.t.	b.t.	b.t.	b.t.	b.t.	b.t.	b.t.	b.t.	b.t.	6,00E+05	6,00E+05	9,00E+05	6,48E+06	7,20E+06	6,84E+06	2,60E+06	2,34E+06	4,00E+06
Q9BRA2	Thioredoxin domain-containing protein 17	b.t.	b.t.	b.t.	5,52E+06	5,06E+06	4,60E+06	b.t.	b.t.	b.t.	b.t.	b.t.	b.t.	b.t.	b.t.	b.t.	1,20E+07	1,08E+07	8,64E+06
Q9BS26	Endoplasmic reticulum resident protein 44	b.t.	b.t.	b.t.	b.t.	b.t.	b.t.	b.t.	b.t.	b.t.	b.t.	b.t.	b.t.	6,66E+06	7,40E+06	7,03E+06	2,20E+06	9,30E+06	4,90E+06
Q9BSQ6	RPL13A protein	b.t.	b.t.	b.t.	b.t.	b.t.	b.t.	b.t.	b.t.	b.t.	1,80E+06	2,70E+06	1,80E+06	3,06E+06	3,40E+06	3,23E+06	4,00E+06	3,60E+06	2,88E+06
Q9BSV4	SFPQ protein	b.t.	b.t.	b.t.	2,52E+06	2,31E+06	2,10E+06	2,40E+06	2,00E+06	1,60E+06	1,33E+06	1,70E+06	9,60E+05	4,86E+06	5,40E+06	5,13E+06	7,90E+06	7,11E+06	5,69E+06
Q9BTT0	Acidic leucine-rich nuclear phosphoprotein 32 family member E	b.t.	b.t.	b.t.	5,52E+06	4,60E+06	4,60E+06	b.t.	b.t.	b.t.	4,90E+06	7,35E+06	4,90E+06	b.t.	b.t.	b.t.	9,30E+06	1,10E+06	8,80E+05
Q9BUF5	Tubulin beta-6	b.t.	b.t.	b.t.	b.t.	b.t.	b.t.	b.t.	b.t.	b.t.	b.t.	b.t.	b.t.	1,08E+07	1,20E+07	1,14E+07	2,30E+06	2,07E+06	1,66E+06
Q9BV28	TUBB3 protein	b.t.	b.t.	b.t.	b.t.	b.t.	b.t.	b.t.	b.t.	b.t.	5,50E+05	5,50E+05	8,25E+05	9,90E+06	1,10E+07	1,05E+07	1,60E+06	1,44E+06	1,15E+06
Q9BV61	TRAP1 protein	b.t.	b.t.	b.t.	3,60E+06	3,20E+06	1,70E+07	4,15E+06	3,10E+06	2,60E+06	7,00E+06	9,80E+06	6,10E+06	1,62E+07	1,80E+07	1,71E+07	5,80E+07	6,80E+06	2,60E+07
Q9BVA1	Tubulin beta-2B	b.t.	b.t.	b.t.	7,80E+05	7,15E+05	6,50E+05	b.t.	b.t.	b.t.	2,20E+06	1,10E+06	3,30E+06	2,88E+07	3,20E+07	3,04E+07	2,88E+06	2,59E+06	2,40E+06
Q9BXP5	Serrate RNA effector molecule homolog	b.t.	b.t.	b.t.	2,57E+06	2,20E+06	2,10E+06	b.t.	b.t.	b.t.	b.t.	b.t.	b.t.	5,67E+06	6,30E+06	5,99E+06	5,76E+06	5,18E+06	4,80E+06
Q9BYN0	Sulfiredoxin-1	b.t.	b.t.	b.t.	6,00E+06	6,00E+06	7,80E+06	2,55E+06	2,13E+06	1,70E+06	8,90E+06	1,34E+07	8,90E+06	2,16E+07	2,40E+07	2,28E+07	1,30E+07	1,17E+07	9,36E+06
Q9H4A4	Aminopeptidase B	b.t.	b.t.	b.t.	5,09E+06	5,00E+06	3,70E+06	b.t.	b.t.	b.t.	1,20E+06	1,20E+06	1,80E+06	1,35E+07	1,50E+07	1,43E+07	4,80E+06	1,40E+07	1,40E+07
Q9H9T3	Elongator complex protein 3	b.t.	b.t.	b.t.	b.t.	b.t.	b.t.	b.t.	b.t.	b.t.	7,50E+06	1,13E+07	7,50E+06	1,62E+07	1,80E+07	1,71E+07	1,50E+07	1,35E+07	1,20E+06
Q9HAV7	GrpE protein homolog 1, mitochondrial	b.t.	b.t.	b.t.	b.t.	b.t.	b.t.	b.t.	b.t.	b.t.	b.t.	b.t.	b.t.	5,76E+06	6,40E+06	6,08E+06	7,70E+06	6,93E+06	5,54E+06
Q9HC38	Glyoxalase domain-containing protein 4	b.t.	b.t.	b.t.	b.t.	b.t.	b.t.	b.t.	b.t.	b.t.	3,00E+06	4,50E+06	3,00E+06	2,61E+06	2,90E+06	2,76E+06	3,10E+06	2,79E+06	2,23E+06
Q9HDC9	Adipocyte plasma membrane-associated protein	b.t.	b.t.	b.t.	b.t.	b.t.	b.t.	b.t.	b.t.	b.t.	b.t.	b.t.	b.t.	7,92E+06	8,80E+06	8,36E+06	1,40E+07	1,50E+07	9,30E+06
Q9NR30	Nucleolar RNA helicase 2	b.t.	b.t.	b.t.	b.t.	b.t.	b.t.	b.t.	b.t.	b.t.	b.t.	b.t.	b.t.	6,21E+06	6,90E+06	6,56E+06	6,50E+06	2,50E+07	4,40E+06
Q9NR45	Sialic acid synthase	b.t.	b.t.	b.t.	5,04E+06	4,62E+06	4,20E+06	b.t.	b.t.	b.t.	b.t.	b.t.	b.t.	8,10E+06	9,00E+06	8,55E+06	b.t.	b.t.	b.t.
Q9NSD9	Phenylalanine--tRNA ligase beta subunit	b.t.	b.t.	b.t.	b.t.	b.t.	b.t.	b.t.	b.t.	b.t.	6,00E+05	6,00E+05	9,00E+05	6,48E+06	7,20E+06	6,84E+06	2,60E+06	2,34E+06	4,00E+06
Q9NSE4	Isoleucine--tRNA ligase, mitochondrial	b.t.	b.t.	b.t.	b.t.	b.t.	b.t.	b.t.	b.t.	b.t.	6,10E+05	6,10E+05	9,15E+05	6,12E+06	6,80E+06	6,46E+06	5,30E+06	4,77E+06	3,82E+06

Q9NTK5	Obg-like ATPase 1b	6,90E+05	1,04E+06	6,90E+05	7,63E+06	7,70E+06	5,40E+06	b.t.	b.t.	b.t.	1,70E+06	1,70E+06	2,55E+06	1,80E+07	2,00E+07	1,90E+07	5,80E+06	5,80E+06	7,60E+06
Q9NYU2	UDP-glucose:glycoprotein glucosyltransferase 1	b.t.	b.t.	b.t.	b.t.	b.t.	b.t.	b.t.	b.t.	b.t.	b.t.	b.t.	b.t.	b.t.	b.t.	b.t.	2,50E+06	2,25E+06	7,20E+05
Q9NZ23	Drug-sensitive protein 1	b.t.	b.t.	b.t.	6,24E+06	5,72E+06	5,20E+06	1,80E+06	1,50E+06	1,20E+06	9,30E+05	9,00E+05	9,60E+05	8,01E+06	8,90E+06	8,46E+06	4,70E+06	4,23E+06	9,50E+06
Q9NZM1	Myoferlin	b.t.	b.t.	b.t.	1,20E+06	1,20E+06	1,56E+06	b.t.	b.t.	b.t.	b.t.	b.t.	b.t.	b.t.	b.t.	b.t.	1,20E+06	1,08E+06	5,50E+06
Q9P2J5	Leucine-tRNA ligase, cytoplasmic	b.t.	b.t.	b.t.	b.t.	b.t.	b.t.	b.t.	b.t.	b.t.	3,70E+06	5,55E+06	3,70E+06	5,04E+06	5,60E+06	5,32E+06	6,12E+06	5,51E+06	5,10E+06
Q9UBQ7	Glyoxylate reductase/hydroxypyruvate reductase	b.t.	b.t.	b.t.	b.t.	b.t.	b.t.	b.t.	b.t.	b.t.	1,90E+06	2,85E+06	1,90E+06	1,53E+07	1,70E+07	1,62E+07	b.t.	b.t.	b.t.
Q9UKK9	ADP-sugar pyrophosphatase	b.t.	b.t.	b.t.	b.t.	b.t.	b.t.	b.t.	b.t.	b.t.	8,90E+05	1,34E+06	8,90E+05	3,60E+06	4,00E+06	3,80E+06	b.t.	b.t.	b.t.
Q9ULV4	Coronin-1C	b.t.	b.t.	b.t.	b.t.	b.t.	b.t.	b.t.	b.t.	b.t.	5,30E+05	5,30E+05	7,95E+05	b.t.	b.t.	b.t.	7,97E+06	5,30E+06	7,60E+06
Q9UMS4	Pre-mRNA-processing factor 19	b.t.	b.t.	b.t.	1,60E+06	1,60E+06	2,08E+06	b.t.	b.t.	b.t.	b.t.	b.t.	b.t.	b.t.	b.t.	b.t.	5,60E+06	5,04E+06	4,03E+06
Q9UNM6	26S proteasome non-ATPase regulatory subunit 13	b.t.	b.t.	b.t.	4,09E+06	3,00E+06	3,70E+06	b.t.	b.t.	b.t.	b.t.	b.t.	b.t.	4,23E+06	4,70E+06	4,47E+06	4,10E+06	1,20E+07	4,70E+06
Q9UNN8	Endothelial protein C receptor	b.t.	b.t.	b.t.	b.t.	b.t.	b.t.	1,02E+06	8,50E+05	6,80E+05	b.t.	b.t.	b.t.	6,30E+06	7,00E+06	6,65E+06	6,90E+06	6,21E+06	4,97E+06
Q9UQM3	Alpha-tubulin	b.t.	b.t.	b.t.	1,13E+06	1,03E+06	9,40E+05	b.t.	b.t.	b.t.	2,50E+05	2,50E+05	3,75E+05	2,79E+07	3,10E+07	2,95E+07	4,90E+06	4,41E+06	1,50E+06
Q9Y265	RuvB-like 1	b.t.	b.t.	b.t.	5,04E+06	3,50E+06	4,70E+06	b.t.	b.t.	b.t.	3,20E+06	1,70E+06	3,60E+06	1,53E+07	1,70E+07	1,62E+07	6,20E+06	6,50E+06	1,70E+07
Q9Y376	Calcium-binding protein 39	b.t.	b.t.	b.t.	2,08E+06	1,50E+06	1,90E+06	b.t.	b.t.	b.t.	b.t.	b.t.	b.t.	4,41E+06	4,90E+06	4,66E+06	b.t.	b.t.	b.t.
Q9Y3E8	CGI-150 protein	b.t.	b.t.	b.t.	b.t.	b.t.	b.t.	b.t.	b.t.	b.t.	3,00E+06	4,50E+06	3,00E+06	2,61E+06	2,90E+06	2,76E+06	3,10E+06	2,79E+06	2,23E+06
Q9Y3U8	60S ribosomal protein L36	b.t.	b.t.	b.t.	b.t.	b.t.	b.t.	b.t.	b.t.	b.t.	b.t.	b.t.	b.t.	1,44E+07	1,60E+07	1,52E+07	b.t.	b.t.	b.t.
Q9Y490	Talin-1	b.t.	b.t.	b.t.	b.t.	b.t.	b.t.	b.t.	b.t.	b.t.	b.t.	b.t.	b.t.	9,00E+06	1,00E+07	9,50E+06	3,84E+06	3,46E+06	3,20E+06
Q9Y4L1	Hypoxia up-regulated protein 1	b.t.	b.t.	b.t.	b.t.	b.t.	b.t.	b.t.	b.t.	b.t.	b.t.	b.t.	b.t.	b.t.	b.t.	b.t.	2,70E+06	2,43E+06	3,40E+06
Q9Y5B9	FACT complex subunit SPT16	b.t.	b.t.	b.t.	2,20E+06	2,20E+06	2,86E+06	b.t.	b.t.	b.t.	b.t.	b.t.	b.t.	b.t.	b.t.	b.t.	4,78E+06	3,40E+06	4,40E+06
Q9Y678	Coatomer subunit gamma-1	b.t.	b.t.	b.t.	1,01E+06	9,24E+05	8,40E+05	2,55E+06	2,13E+06	1,70E+06	b.t.	b.t.	b.t.	3,78E+06	4,20E+06	3,99E+06	7,40E+06	7,70E+06	6,16E+06
Q9Y6E2	Basic leucine zipper and W2 domain-containing protein 2	b.t.	b.t.	b.t.	b.t.	b.t.	b.t.	b.t.	b.t.	b.t.	b.t.	b.t.	b.t.	7,20E+06	8,00E+06	7,60E+06	2,90E+06	2,61E+06	5,20E+06
Q9Y6E3	HSPC027	b.t.	b.t.	b.t.	4,09E+06	3,00E+06	3,70E+06	b.t.	b.t.	b.t.	b.t.	b.t.	b.t.	4,23E+06	4,70E+06	4,47E+06	4,10E+06	1,20E+07	4,70E+06
R4GMR5	26S proteasome non-ATPase regulatory subunit 8	b.t.	b.t.	b.t.	9,96E+05	9,13E+05	8,30E+05	b.t.	b.t.	b.t.	b.t.	b.t.	b.t.	6,66E+06	7,40E+06	7,03E+06	6,20E+06	5,58E+06	4,46E+06
R4GNA8	P55 protein	b.t.	b.t.	b.t.	2,20E+06	2,20E+06	2,86E+06	b.t.	b.t.	b.t.	b.t.	b.t.	b.t.	b.t.	b.t.	b.t.	4,78E+06	3,40E+06	4,40E+06
R4SBI6	EPHX1	1,04E+06	8,80E+05	1,20E+06	1,63E+07	2,20E+07	7,60E+06	b.t.	b.t.	b.t.	3,00E+06	2,10E+06	3,90E+06	1,89E+07	2,10E+07	2,00E+07	9,40E+06	3,00E+07	2,10E+07
S4R435	Protein RPS10-NUDT3	b.t.	b.t.	b.t.	9,48E+06	8,69E+06	7,90E+06	8,25E+06	6,88E+06	5,50E+06	1,20E+06	1,80E+06	2,40E+06	4,50E+06	5,00E+06	4,75E+06	7,90E+07	7,11E+07	5,69E+07
V9HW26	ATP synthase subunit alpha	b.t.	b.t.	b.t.	3,25E+06	3,70E+06	2,00E+06	b.t.	b.t.	b.t.	3,25E+06	1,90E+06	4,60E+06	2,16E+07	2,40E+07	2,28E+07	1,00E+07	1,40E+07	1,80E+07
V9HW31	ATP synthase subunit beta	b.t.	b.t.	b.t.	3,24E+06	2,97E+06	2,70E+06	b.t.	b.t.	b.t.	6,20E+06	3,80E+06	8,60E+06	2,61E+07	2,90E+07	2,76E+07	2,20E+07	1,80E+07	2,70E+07
V9HW62	Lactoylglutathione lyase	b.t.	b.t.	b.t.	b.t.	b.t.	b.t.	b.t.	b.t.	b.t.	1,80E+06	2,70E+06	1,80E+06	2,43E+06	2,70E+06	2,57E+06	7,30E+06	6,57E+06	5,26E+06
V9HWB8	Pyruvate kinase	b.t.	b.t.	b.t.	7,47E+06	5,00E+06	7,10E+06	6,60E+06	5,50E+06	4,40E+06	2,00E+06	2,00E+06	3,00E+06	2,16E+07	2,40E+07	2,28E+07	9,80E+06	3,50E+06	1,40E+07
V9HWC9	Superoxide dismutase [Cu-Zn]	b.t.	b.t.	b.t.	b.t.	b.t.	b.t.	b.t.	b.t.	b.t.	b.t.	b.t.	b.t.	1,80E+07	2,00E+07	1,90E+07	1,30E+07	1,17E+07	9,36E+06
V9HWH2	Creatine kinase brain isoform 1	b.t.	b.t.	b.t.	b.t.	b.t.	b.t.	b.t.	b.t.	b.t.	1,80E+06	2,70E+06	1,80E+06	7,92E+06	8,80E+06	8,36E+06	2,40E+06	2,16E+06	5,20E+06
V9HWI3	Cathepsin D	b.t.	b.t.	b.t.	3,74E+06	4,40E+06	2,20E+06	1,29E+06	1,08E+06	8,60E+05	b.t.	b.t.	b.t.	1,08E+07	1,20E+07	1,14E+07	2,80E+07	2,52E+07	1,20E+06
V9HWJ1	Glutathione synthetase	b.t.	b.t.	b.t.	8,88E+06	1,30E+07	3,40E+06	b.t.	b.t.	b.t.	1,91E+06	8,20E+05	3,00E+06	7,92E+06	8,80E+06	8,36E+06	5,70E+06	9,20E+06	9,10E+06
V9HWJ2	Isocitrate dehydrogenase [NADP]	2,60E+06	2,60E+06	3,90E+06	2,36E+07	2,20E+07	1,80E+07	3,30E+06	2,75E+06	2,20E+06	8,30E+06	6,30E+06	8,20E+06	5,58E+07	6,20E+07	5,89E+07	2,00E+07	2,00E+07	3,50E+07
X5D2T3	Ribosomal protein L10 isoform A	b.t.	b.t.	b.t.	b.t.	b.t.	b.t.	b.t.	b.t.	b.t.	5,10E+06	7,65E+06	5,10E+06	4,68E+06	5,20E+06	4,94E+06	6,10E+06	5,49E+06	4,39E+06
X5D7E3	Eukaryotic translation initiation factor 4E isoform A	b.t.	b.t.	b.t.	b.t.	b.t.	b.t.	b.t.	b.t.	b.t.	b.t.	b.t.	b.t.	4,23E+06	4,70E+06	4,47E+06	1,20E+07	1,08E+07	8,64E+06
X5DR03	Glutathione S-transferase isoform B	b.t.	b.t.	b.t.	b.t.	b.t.	b.t.	b.t.	b.t.	b.t.	b.t.	b.t.	b.t.	b.t.	b.t.	b.t.	1,30E+07	1,17E+07	9,36E+06
X6RA14	S-formylglutathione hydrolase	b.t.	b.t.	b.t.	b.t.	b.t.	b.t.	b.t.	b.t.	b.t.	b.t.	b.t.	b.t.	1,08E+07	1,20E+07	1,14E+07	3,60E+06	3,24E+06	2,59E+06
